# Novel Molecular Signatures in the PIP4K/PIP5K Family of Proteins Specific for Different Isozymes and Subfamilies Provide Important Insights into the Evolutionary Divergence of this Protein Family

**DOI:** 10.3390/genes10040312

**Published:** 2019-04-21

**Authors:** Bijendra Khadka, Radhey S. Gupta

**Affiliations:** Department of Biochemistry and Biomedical Sciences McMaster University, Hamilton, ON L8N 3Z5, Canada; khadkab@mcmaster.ca

**Keywords:** phosphatidylinositol phosphate kinases, conserved signature indels, molecular signatures for the pip4k/pip5k isozymes and isoforms, phylogenetic analysis, species distribution of pip4k/pip5k proteins, holozoa clade of eukaryotic organisms, evolution of the PIP4K/PIP5K family of proteins, protein evolution

## Abstract

Members of the PIP4K/PIP5K family of proteins, which generate the highly important secondary messenger phosphatidylinositol-4,5-bisphosphate, play central roles in regulating diverse signaling pathways. In eukaryotic organisms, multiple isozymes and subfamilies of PIP4K/PIP5K proteins are found and it is of much interest to understand their evolution and species distribution and what unique molecular and biochemical characteristics distinguish specific isozymes and subfamilies of proteins. We report here the species distribution of different PIP4K/PIP5K family of proteins in eukaryotic organisms and phylogenetic analysis based on their protein sequences. Our results indicate that the distinct homologs of both PIP4K and PIP5K are found in different organisms belonging to the Holozoa clade of eukaryotes, which comprises of various metazoan phyla as well as their close unicellular relatives Choanoflagellates and Filasterea. In contrast, the deeper-branching eukaryotic lineages, as well as plants and fungi, contain only a single homolog of the PIP4K/PIP5K proteins. In parallel, our comparative analyses of PIP4K/PIP5K protein sequences have identified six highly-specific molecular markers consisting of conserved signature indels (CSIs) that are uniquely shared by either the PIP4K or PIP5K proteins, or both, or specific subfamilies of these proteins. Of these molecular markers, 2 CSIs are distinctive characteristics of all PIP4K homologs, 1 CSI distinguishes the PIP4K and PIP5K homologs from the Holozoa clade of species from the ancestral form of PIP4K/PIP5K found in deeper-branching eukaryotic lineages. The remaining three CSIs are specific for the PIP5Kα, PIP5Kβ, and PIP4Kγ subfamilies of proteins from vertebrate species. These molecular markers provide important means for distinguishing different PIP4K/PIP5K isozymes as well as some of their subfamilies. In addition, the distribution patterns of these markers in different isozymes provide important insights into the evolutionary divergence of PIP4K/PIP5K proteins. Our results support the view that the Holozoa clade of eukaryotic organisms shared a common ancestor exclusive of the other eukaryotic lineages and that the initial gene duplication event leading to the divergence of distinct types of PIP4K and PIP5K homologs occurred in a common ancestor of this clade. Based on the results gleaned from different studies presented here, a model for the evolutionary divergence of the PIP4K/PIP5K family of proteins is presented.

## 1. Introduction

The members of the PIP4K/PIP5K family of proteins play key roles in the synthesis and regulation of various phosphoinositides (PIs), which act as a secondary messenger for controlling diverse cellular processes in eukaryotes [1,2,3]. These proteins (or isozymes) are classified into three distantly related groups of proteins viz. (i) Phosphatidylinositol-4-phosphate 5-kinase Type I (PIP5K), (ii) phosphatidylinositol-5-phosphate 4-kinase Type II (PIP4K), and (iii) phosphatidylinositol-3-phosphate 5-kinase Type III (or PIKFYVE) [4,5]. The PIP5K family of isozymes, which catalyze the conversion of phosphatidylinositol-4-phosphate (PI4P) to phosphatidylinositol-4,5-bisphosphate (PI(4,5)P_2_), are mainly localized to the plasma membrane, Golgi complex, and nucleus in mammalian cells [6,7]. In vertebrates, three subfamilies or isoforms (encoded by separate genes) of PIP5K (viz. PIP5Kα, PIP5Kβ, and PIP5Kγ) are present along with multiple splice variants of these isoforms generated as a result of alternative splicing [8]. For instance, in humans, three splice variants of PIP5Kα, four splice variants of PIP5Kβ, and three splice variants of PIP5Kγ have been reported [5,8,9]. The PIP4K on the other hand, which catalyzes the phosphorylation of the 4-hydroxyl group of phosphatidylinositol-5-phosphate (PI5P), are diffusively localized in the cytoplasm, endoplasmic reticulum, actin cytoskeleton, and nucleus [10]. As with the PIP5K, three subfamilies of PIP4K (viz. PIP4Kα, PIP4Kβ, and PIP4Kγ encoded by separate genes) are also found in vertebrates [4,11]. The presence of multiple distinct isoforms of PIP4K/PIP5K in vertebrate species is predicted to enable these organisms to coordinate the regulation of PI(4,5)P_2_ production for specific processes, either by differential regulation or selective subcellular localization of the individual copies of these enzymes and their isoforms [3,5,8,10,12]. In both PIP5K and PIP4K families, the core kinase domain is highly conserved whereas the regions outside this domain show limited sequence similarity [4,13].

Unlike vertebrates which contain multiples subfamilies of both PIP4K and PIP5K, all invertebrates including Coelomates (Deuterostomes, Protostomata), and Pseudocoelomate have been reported to contain only a single homolog of both these proteins [4]. In fungi, a single homolog of PIP4K/PIP5K showing similarity to both PIP4K and PIP5K is found (referred to as a multiple-copy suppressor of stt4 mutation, MSS4 protein) [14,15]. Plants also contain multiple copies of a protein showing similarity to both PIP4K/PIP5K [16,17,18]. However, the plants and fungi proteins contain distinct structural features/domains, which are not found in the PIP4K/PIP5K homologs from vertebrates and invertebrates species, suggesting that they perform additional novel functions which are specific for these organisms [17,18,19]. The PYKFYVE, which catalyzes the phosphorylation of PI3P to PI(3,5)P_2_ [20], is considerably larger than the PIP4K and PIP5K homologs and it shows very limited similarity to the PIP4K/PIP5K proteins, restricted to the kinase domain.

Due to their important cellular roles, the PIP4K/PIP5K family of isozymes have been studied extensively [21,22,23]. However, a detailed understanding of the species distribution of these proteins, how the different isozymes and subfamilies of these proteins have evolved and diversified, and novel sequence features that are specific for these two isozymes or their different forms which are present in vertebrate species, remain largely enigmatic and unexplored. In recent years, genome sequences have become available from diverse eukaryotic organisms providing a valuable resource for identifying novel molecular markers/characteristics that are uniquely found in specific proteins from an evolutionarily related group of organisms, or those that are commonly shared by members from a related group/family of proteins. One important class of molecular markers discovered by genome sequence analysis, which has proven very useful for evolutionary, genetic and biochemical studies, is comprised of conserved signature indels (insertions/deletions) (CSIs) in gene/protein sequences [24,25,26,27,28,29]. The CSIs in gene/protein sequences generally result from rare genetic changes and based upon their presence or absence in different species or homologs, important inferences regarding evolutionary relationships can be derived. In our recent work, multiple CSIs were identified within the catalytic domain of the diacylglycerol kinase (DGK) family of isozymes which were either specific for a particular class of isozyme or commonly shared by two or more classes of DGK isozymes, thereby providing important insights into the evolutionary history of this protein family [30].

In the present study, we have used a combination of phylogenetic approaches and the CSI identification strategy to understand the evolutionary relationships and the origin/distribution of the PIP4K/PIP5K family of proteins. Our analyses of this protein family have identified six CSIs that are specific characteristics of either all or particular types and subfamilies of the PIP4K/PIP5K proteins revealing their novel sequence features and providing important insights into their evolutionary history. The identified CSIs provide novel tools for functional studies on these proteins and we discuss here the implications of the results presented here for the origin and evolutionary diversification of the PIP4K/PIP5K family of proteins.

## 2. Materials and Methods

### 2.1. Identification of Conserved Signature Indels and Phylogenetic Analysis

Protein sequences for the PIP4K/PIP5K family were obtained from the NCBI database [31]. Identification of CSIs (insertions/deletions) in the sequence alignments of these proteins was carried out as described in our earlier work [30,32,33]. In brief, homologs of the PIP4K/PIP5K family proteins from [4,9,13] representative species from major eukaryotic groups were retrieved from the NCBI database and multiple sequence alignments of these proteins were created separately and in combination using the Clustal X program [34]. The α, β, and γ isoforms or subfamilies of the PIP4K and PIP5K proteins present in vertebrates that were analyzed in the present work are encoded for by distinct genes. Although the term isoform is commonly used to refer to these proteins, these proteins are products of separate genes and not derived using alternative splicing. Sequence alignments of the proteins were visually inspected for the presence of CSIs that were flanked on both sides by at least 3–4 conserved amino acids in the neighboring 40–50 amino acids. Indels which were not flanked by conserved regions were not further studied. For all conserved indels thus identified, detailed BLASTp searches were carried out on short sequence segments containing the indel and its flanking conserved regions (60–100 amino acids long) to determine the specificity and species distribution of the indels in PIP4K/PIP5K homologs from different organisms. The CSIs figures shown here were generated using SIG_CREATE and SIG_STYLE programs (from www.GLEANS.net) as described in our earlier work [32,35]. Due to space constraints, sequence information for the PIP4K/PIP5K homologs is shown in the presented figures for only representative species from different groups of organisms. However, unless otherwise indicated, the identified CSIs are specific for the indicated PIP4K/PIP5K isozymes/isoforms for the indicated groups of eukaryotic organisms and detailed information regarding the species distribution of different described CSIs is provided in Figures S2–S7.

For the construction of the phylogenetic tree, sequences for the PIP4K, PIP5K, and PIP4K/PIP5K proteins from different organisms were trimmed to correspond to the core catalytic kinase domain, which is conserved among these homologs. Multiple sequence alignment of the core catalytic kinase domain which consisted of 217 aligned amino acid positions was used for phylogenetic analysis. A maximum-likelihood phylogenetic tree based on 100 bootstrap replicates of this sequence alignment was constructed using MEGA 6 [36] based on the Jones–Taylor–Thornton (JTT) model [37], as detailed in our earlier studies [30,33,38].

### 2.2. Homology Modelling and Structural Analyses

Homology modeling of the CSI-containing and CSI-lacking PIP5K family of proteins was carried out as described in earlier work using an in-house pipeline, “GlabModeller,” for comparative protein structure modeling [33,38,39]. Initially, to identify appropriate templates for homology modeling, PSI-BLAST [40] searches were carried out against the Protein Data Bank (PDB) [41] using protein sequences from the PIP4K/PIP5K homologs. The suitable templates identified by blast searches include, PIP5K-α (*Danio rerio*) (PDB ID: 4TZ7, chain “A”), PIP4K-β (*Homo sapiens*) (PDB ID: 1BO1, chain “B”), PIP4K-γ (*H. sapiens*) (PDB ID: 2GK9, chain “A”), PIP4K-α (*H. sapiens*) (PDB ID: 2YBX, chain “A”) [42,43] and exhibits sequence identities of 71%, 33%, 32%, 30% with the whole sequence of PIP5Kβ (*H. sapiens*) (Accession number: AAH30587.1). For homology modeling, the sequence alignments between target and template proteins were carried out using the align 2D module from the Modeller, which is integrated and streamlined in GlabModeller tool. The resulting alignments obtained were then carefully analyzed and modified manually to ensure the reliability of the location of insertion and deletions. For each target protein, 500 models were generated initially and ranked and selected using the discrete optimized potential energy (DOPE) score [44] as implemented in Modeller v9.15 [45]. The models with high DOPE scores are then submitted to the ModRefiner program to obtain atomic-level energy minimization and to obtain a model with reliable stereochemistry quality [46]. The illustrative approach was utilized to improve the overall quality of the final model. Loop regions are refined using the ModLoop server [47]. The qualities of the final models were analyzed using a number of different independent protein model validation servers/tools which are integrated into the GlabModeller. The servers/tool for validation utilized include RAMPAGE [48], ProSA [49,50], and QMEAN [51]. Visualization and structural analysis of structural models of PIP4K/PIP5K proteins were carried out using the molecular visualization program PyMOL (www.pymol.org).

## 3. Results

### 3.1. Species Distribution and Phylogenetic Analysis of PIP4K/PIP5K Protein Family

As noted in the introduction, in contrast to all vertebrates and some invertebrate species (viz. belonging to the superphyla Deutrostomia, Protostomia and Pseudocoelomates), which contain distinct homologs of both PIP4K and PIP5K proteins, plants and fungi contain only a single homolog of these proteins showing similarity to both PIP4K and PIP5K homologs [4]. However, the distribution of these two proteins in other early branching metazoan lineages such as Placozoa, Porifera, Cnidaria, and Ctenophora, or their known sister groups which includes Choanoflagellates and Filasterea, remains undetermined [52,53,54,55]. Hence, the distribution of PIP4K and PIP5K isozymes in these early branching metazoan/eukaryotic lineages is of much importance for understanding the evolutionary diversification of PIP4K and PIP5K isozymes within the eukaryotes.

BLASTp searches were carried out with the sequences for PIP4K and PIP5K proteins against the NCBI nr database as well as genome sequences from different eukaryotic organisms. The results from these studies, which are summarized in Table 1, show that distinct homologs of both PIP4K and PIP5K are found in all major metazoan groups (i.e., Bilateria, Cnidaria, Placozoa, and Porifera) as well as in their closest-known unicellular ancestor, Choanoflagellates. Homologs for PIP4K and PIP5K were also found in a Filasterea species (*Capsaspora owczarzaki*), however, no sequence sharing similarity to PIP4K or PIP5K was detected in Ichthyosporea. Outside of the metazoans, Choanoflagellates and Filasterea, only a single homolog of PIP4K/PIP5K was detected in other available genomes from eukaryotic organisms including those from the phyla Apicompelxa, Amebozoa, Percolzoa, and Apusozoa. Further, as known from earlier work [4,11,33], single orthologs exhibiting similarity to both PIP4K and PIP5K were detected in different plants and fungi.

**Figure 1:**
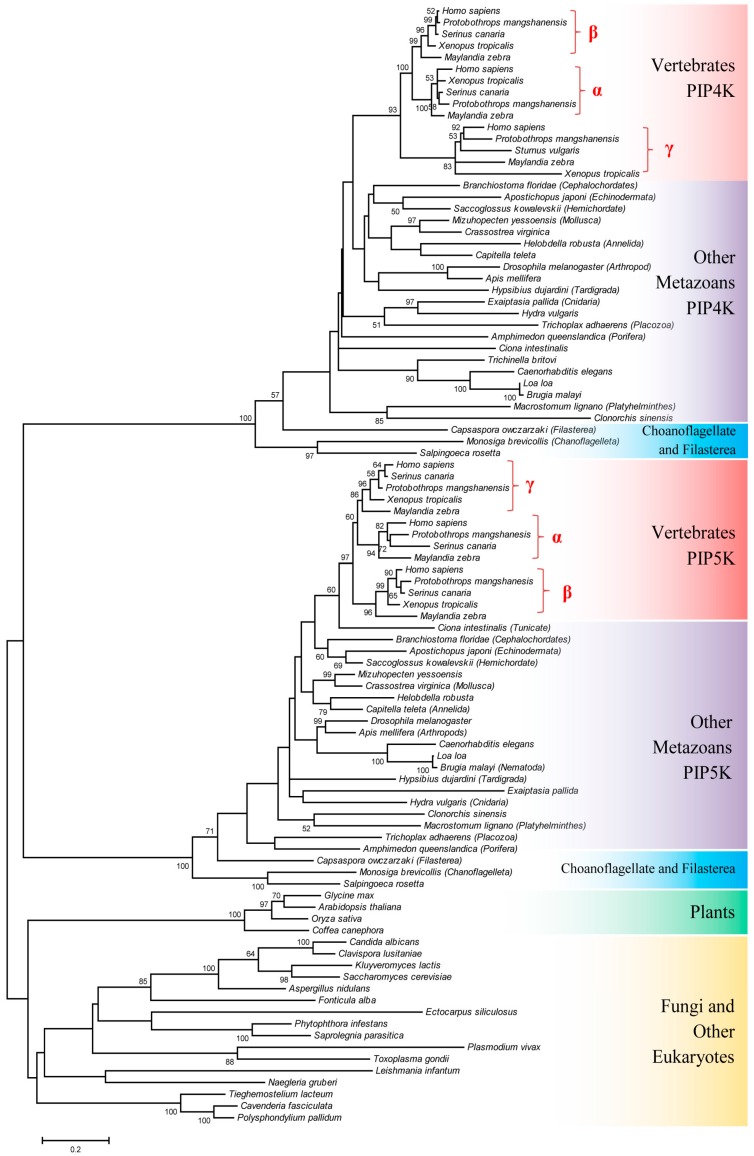
A maximum-likelihood phylogenetic tree of the PIP4K/PIP5K family of proteins based on the core conserved kinase domain region of the protein sequences from representative species. The accession numbers of the protein sequences that were utilized are provided in Table S1. Bootstrap support values > 50% are shown at the nodes.

The evolutionary relationship among the PIP4K/PIP5K homologs from different eukaryotic organisms was further investigated by constructing a maximum-likelihood phylogenetic tree based on sequence alignments for the core catalytic domain which is conserved in all members of this protein family. This tree encompasses 96 protein sequences and it includes different PIP4K/PIP5K homologs from representative species of all major taxonomic groups within eukaryotes (Figure 1).

As seen from Figure 1, the PIP4K and PIP5K homologs from different metazoan species form two strongly supported clusters in the tree and they are separated by a long branch from a cluster comprising of the single homologs of PIP4K/PIP5K found in the deeper branching eukaryotic lineages as well as in plants and fungi. However, the overall support for this latter cluster, as well as the interrelationships of different species within it, is generally weak and not reliably resolved. Nonetheless, based on this phylogenetic tree (Figure 1), several inferences can be drawn: (i) Within the clusters corresponding to PIP4K and PIP5K homologs, although the interrelationships of different metazoans species are not resolved, in both cases the Choanoflagellates and Filasterea species, which are unicellular organisms most closely related to the multicellular metazoans, form the deepest branching lineages in the clusters. (ii) The vertebrate species form strongly supported clades in both PIP4K and PIP5K clusters. The three different families (or types) of PIP4K and PIP5K proteins (viz. α, β and γ) which are found in vertebrate species also formed distinct clades, consistent with earlier studies [4,11]. (iii) Based on their branching in the phylogenetic tree, for PIP4K homologs, the γ-subfamily of the protein exhibited the deepest branching and it formed a sister group of a clade consisting of the PIP4Kα and PIP4Kβ proteins. On the other hand, for the PIP5K protein, the β subfamily showed the earliest divergence followed by the emergence of α and γ paralogs of the proteins. (iv) In the phylogenetic tree, the cluster consisting of the PIP5K homologs exhibited a closer relationship to the cluster comprising of the single copy homologs of PIP4K/PIP5K found in the deeper branching eukaryotic lineages.

### 3.2. Conserved Signature Indels that are Distinctive Features of the PIP4K and PIP5K Family of Proteins and the Insights Provided by Them into the Evolutionary Relationships

Based on the phylogenetic tree in Figure 1 although some inferences regarding evolutionary relationships amongst PIP4K and PIP5K can be drawn, due to poor statistical support and separation by long branches of many significant nodes, it is important to confirm these inferences by other independent approaches. CSIs represent an important class of molecular markers that have been used in the past to resolve a number of important evolutionary questions [24,26,27,29,56]. The CSIs in gene/protein sequences generally result from rare genetic changes. Due to the discrete nature of genetic changes represented by CSIs and their presence in conserved regions, the presence or absence of CSIs in different lineages (or proteins) is generally not affected by factors that can confound branching in phylogenetic trees [26,27,29,56]. In view of the usefulness of CSIs for evolutionary studies, sequence alignments of the PIP4K/PIP5K proteins were examined for the presence of any useful/informative CSIs.

**Figure 2:**
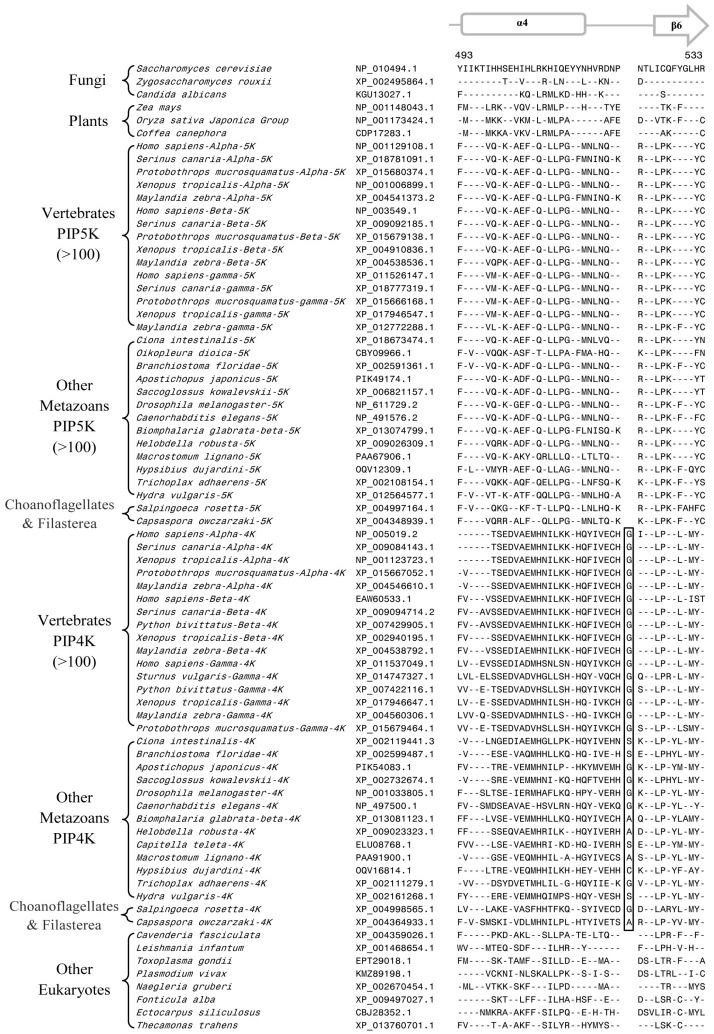
Excerpts from the sequence alignment of PIP4K and PIP5K homologs showing a 1 aa insert (boxed) in a conserved region that is uniquely shared by all PIP4K homologs. This insert is commonly shared by all PIP4K homologs from metazoan phyla including the Choanoflagellates and Filasterea but it is not found in any PIP5K homologs or the single orthologs of PIP4K/PIP5K found in the deeper-branching eukaryotic organisms. Detailed information regarding the species distribution of this conserved signature indels provided in Figure S2. The dashes (-) in the alignment indicates identity with the amino acids on the top line. Numbers on the top indicate the location of this sequence region in the protein from *Saccharomyces cerevisiae*. The accession numbers of various sequences are given in the second column. Secondary structure information for this protein region is presented on top of the sequence.

**Figure 3:**
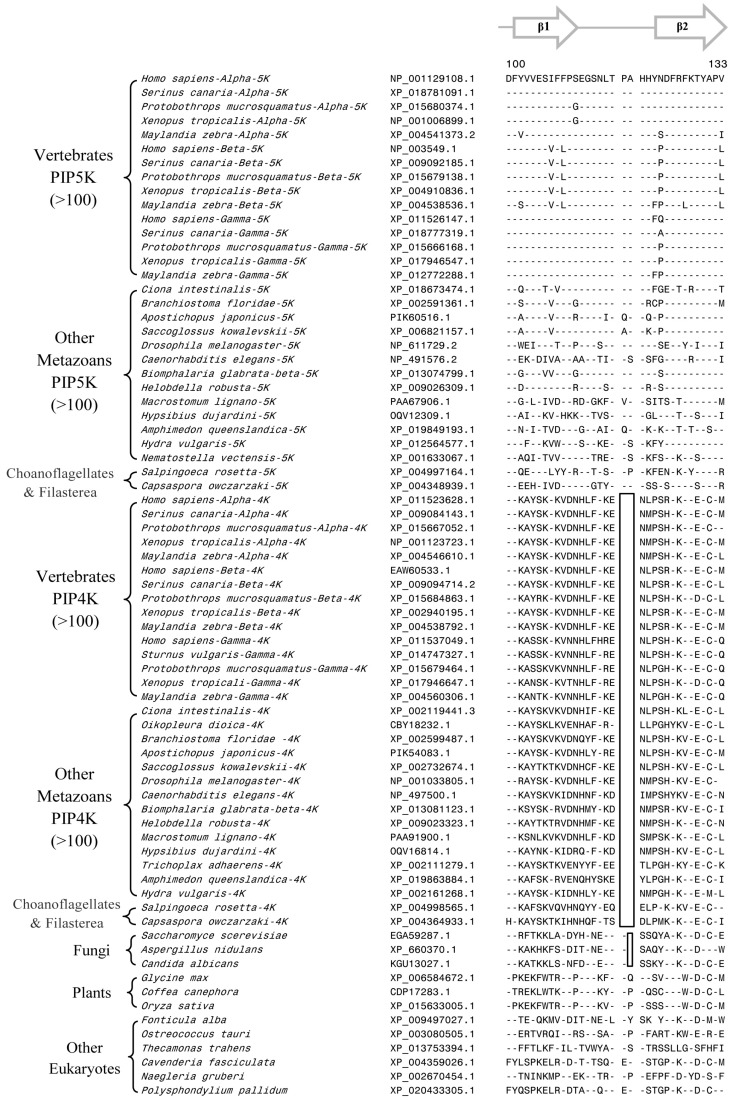
Partial sequence alignment of the PIP4K/PIP5K family of proteins showing a 2 aa deletion in a conserved region (boxed) that is uniquely shared by all PIP4K homologs. The boxed CSI is not present in any of the PIP5K homologs as well as the PIP4K/PIP5K orthologs from plants and other deep-branching eukaryotic lineages. The PIP4K/PIP5Korthologs from fungi contain a shorter 1 aa deletion in this position. More detailed sequence information for this CSI is provided in Figure S3. Other details are the same as in Figure 2 legend. Numbers on the top indicate the position of this sequence in the human PIP5Kα.

Our analysis of PIP4K/PIP5K protein sequences has identified several useful CSIs, which provide important insights into the evolution of this family of proteins. Of these CSIs, two CSIs are shared by all PIP4K homologs, but they are lacking in all PIP5K homologs as well as the single homolog of the PIP4K/PIP5K found in the deeper branching eukaryotic organisms and plants and fungi (Figure 2 and Figure 3). The first of these CSIs consists of 1 aa insert in the kinase homology domain (Figure 2), whereas the second CSI is a 2 aa deletion in the N-terminal domain of the PIP4K protein (Figure 3). Sequence information for these CSIs for PIP4K/PIP5K homologs for representative species from the major groups of eukaryotes are shown in Figure 2 and Figure 3. More detailed information regarding the species distribution of these CSIs is provided in Figures S2 and S3.

Sequence alignments of the PIP4K/PIP5K proteins shown in Figure 2 and Figure 3 illustrate that the distinct homologs of both PIP4K and PIP5K are present in different Deutrostomia and Protostomia species as well in other deeper branching phyla of Animalia such as Placozoa (*Trichoplax adhaerens*), Porifera (*Amphimedon queenslandica*), and Cnidaria (*Exaiptasia pallidia* and *Hydra vulgaris*). Further, in addition to the Animalia species, distinct homologs of PIP4K and PIP5K are also present in the Choanoflagellates (*Salpinogoeca rosetta* and *Monosiga brevicollis*) and Filasterea (*C. owczarzaki*) species whose members are indicated to be the closest unicellular relatives of multicellular Animalia [57,58]. As seen from Figure 2 and Figure 3, the two CSIs in the PIP4K homologs are shared by all orthologs of this protein including those from the unicellular metazoan phyla viz. Choanoflagellate and Filasterea, but they are not present in any of the PIP5K homologs from corresponding phyla. Further, the single homolog of PIP4K/PIP5K proteins found in the deeper branching eukaryotic lineages, such as Apicompelxa, Amebozoa, Percolozoa, and Apusozoa also lack the indicated CSIs. The absence of these CSIs in the deeper branching eukaryotic phyla indicates that the 1 aa CSI constitutes an insert in the PIP4K family of proteins, whereas the 2 aa CSI is a deletion in this protein. Based on the unique shared presence of these CSIs in all PIP4K homologs, the genetic changes responsible for these CSIs are postulated to have occurred in a common ancestor of the PIP4K family of protein at the time when the PIP4K and PIP5K family of proteins diverged from a common ancestor by a gene duplication event.

**Figure 4:**
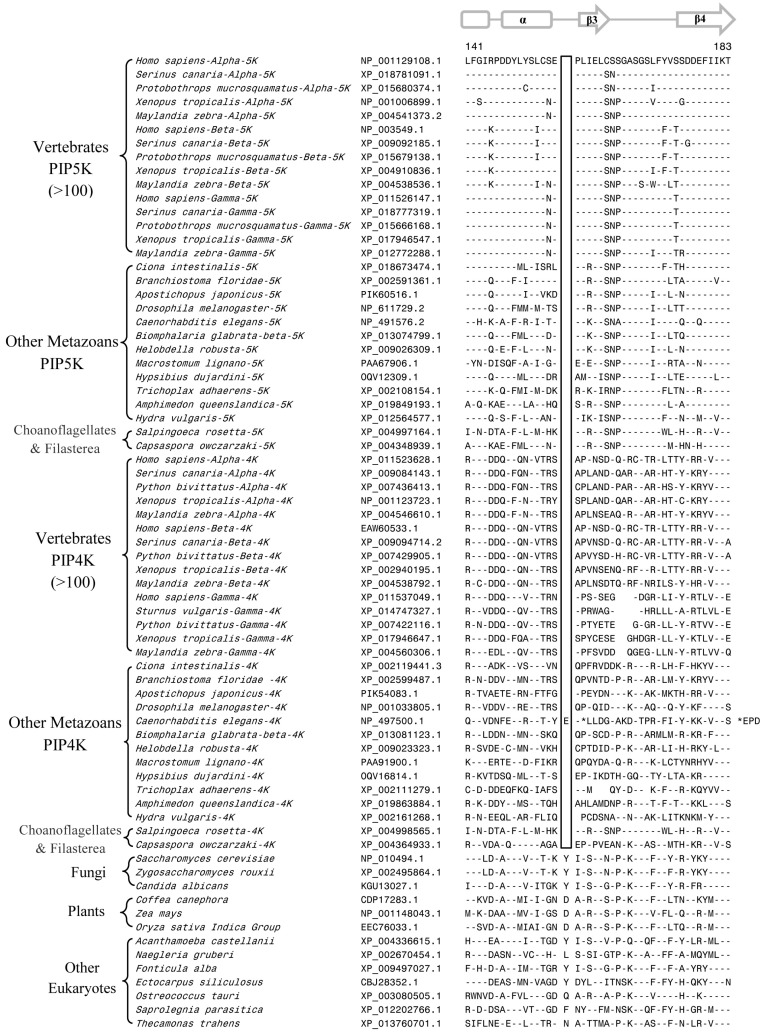
Partial sequence alignment of PIP4K/PIP5K family of proteins showing 1 aa deletion (boxed) in a conserved region that is commonly shared by different PIP4K and PIP5K homologs but lacking in the single copy PIP4K/PIP5K orthologs from deeper branching eukaryotic phyla including plants and fungi. This 1 aa CSI distinguishes the distinct PIP4K and PIP5K homologs found in Holozoa species (i.e., all multicellular metazoan species and their unicellular relatives Choanoflagellates and Filasterea) from early branching eukaryotic organisms harboring only a single ortholog of the PIP4K/PIP5K protein. The PIP4K from some nematode species lack this deletion or contain a longer insertion (indicated by *) in this position and its possible significance is unclear. Numbers on the top indicate the position in human PIP5Kα. More detailed information for this CSI is provided in Figure S4.

Another important CSI identified by our analysis consists of a 1aa deletion within the highly conserved kinase homology domain that is commonly shared by all PIP4K and PIP5K homologs (and their distinct isoforms), but which is not found in the single homolog of these proteins present in fungi, plants, and the deep branching eukaryotic lineages (Figure 4).

The shared presence of this CSI in different PIP4K and PIP5K homologs from all metazoan species well as in the Choanoflagellates and Filasterea phyla, which together comprise the Holozoa clade of organisms [59,60], strongly suggests that the genetic change responsible for this CSI occurred in a common ancestor of the Holozoa. Further, due to the shared presence of this CSI in both PIP4K and PIP5K homologs, the genetic change leading to this CSIs is postulated to have occurred before gene duplication leading to distinct forms of the PIP4K and PIP5K proteins, which are present in different Holozoa species.

**Figure 5:**
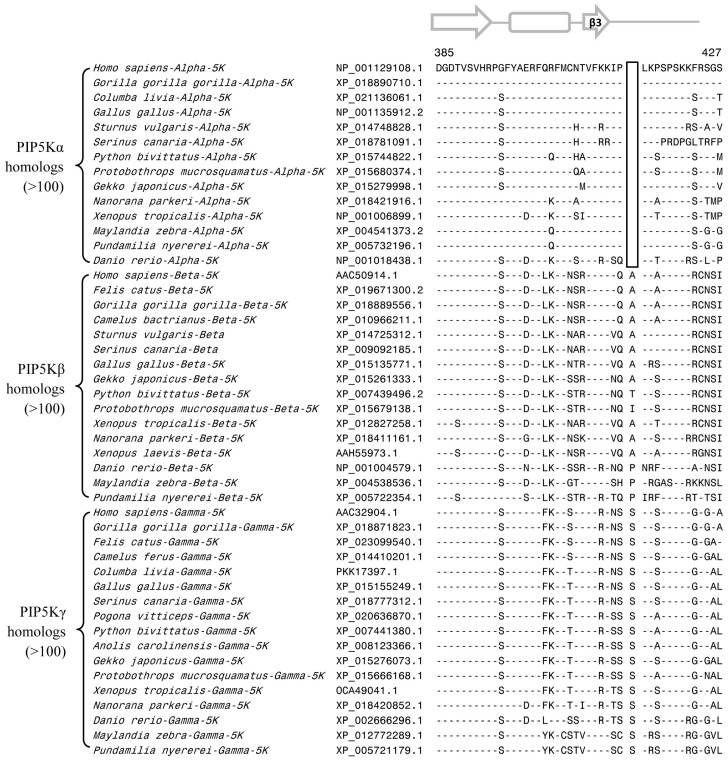
Partial sequence alignment of different subfamilies of the PIP5K protein showing 1 aa CSI (boxed) that is uniquely shared by the PIP5Kα subfamily of proteins. More detailed information regarding species distribution of this CSI is provided in Fig. S5. The predicted secondary structure of this sequence region is shown on top of the sequence alignment. Other details are the same as in the Figure 2 legend.

In addition to the CSIs that are specific for the PIP4K or PIP5K proteins, or both, our analysis has identified three other CSIs that are uniquely shared by members of specific subfamilies of the PIP4K and PIP5K proteins, distinguishing them from other related proteins and providing useful insights concerning their evolution. The first of these subfamily-specific CSIs consists of a 1 aa deletion that is uniquely present in all PIP5Kα homologs but not found in the PIP5Kβ and PIP5Kγ homologs (Figure 5). The observed CSI (1aa deletion) is located within the C-terminal region of the conserved core kinase homology domain, as shown in Figure 5. The region where this CSI is found is conserved in the PIP5K family of proteins, but it is lacking in the PIP4K homologs. Due to the specificity of this CSI for PIP5Kα, the genetic change responsible for this CSI is postulated to have occurred in a common ancestor of the PIP5Kα proteins, and it provides a reliable molecular characteristic distinguishing the PIP5Kα from PIP5Kβ and PIP5Kγ subfamily of proteins.

**Figure 6:**
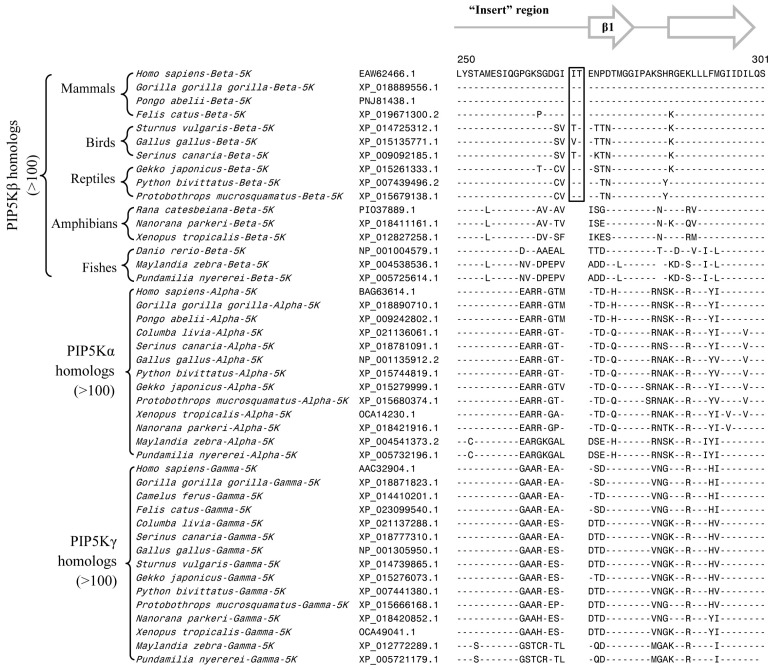
Excerpts from the multiple sequence alignment of PIP5K homologs showing a 2 aa insert in a conserved region (boxed) that is uniquely shared by the PIP5Kβ homologs of mammals, birds, and reptiles, but absent from all other PIP4K and PIP5K homologs. Sequence information is shown here for only representative species from different vertebrate classes. More detailed information for this CSI is provided in Figure S6. Other details are as in Figure 2 legend. Numbers on the top indicate the position in the human PIP5Kβ.

Another subfamily-specific CSI identified in our work consists of a 2 aa conserved insert that is commonly shared by the PIP5Kβ family of proteins from mammals, birds, and reptile species, but not found in the PIP5Kβ homologs from amphibians and fish (Figure 6). This CSI is absent in the PIP5Kα and PIP5Kγ family members and also in different PIP5K homologs. Based on its distribution pattern, the genetic change leading to this CSI is postulated to have occurred in a common ancestor of the PIP5Kβ from mammals, birds, and reptiles and it supports the latter divergence of these vertebrates classes in comparison to the fishes and amphibians [61].

**Figure 7:**
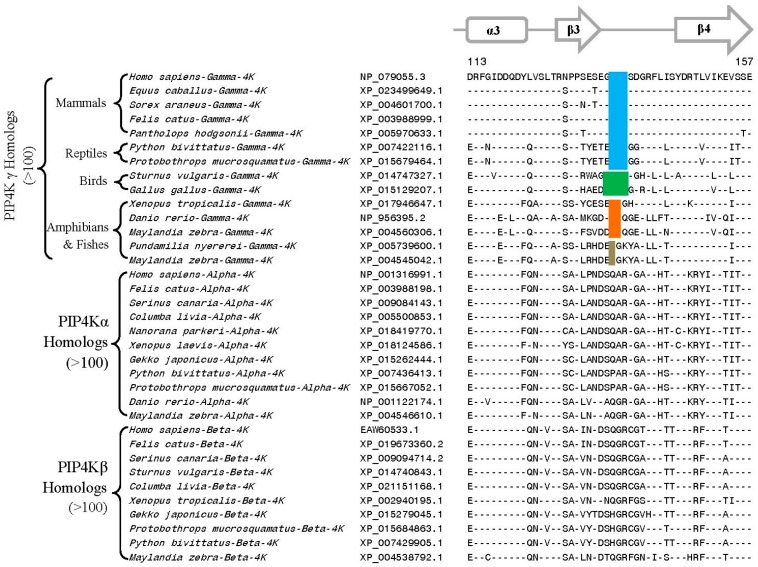
Partial sequence alignment of different isoforms of the PIP4K proteins showing a conserved region where 1–4 aa deletions (boxed) are uniquely found in the PIP4Kγ subfamily of proteins from different classes of vertebrates. The PIP4Kγ protein mammals and reptiles have a 3 aa deletion in this position, whereas those from the birds contain a 4 aa deletion in the same position. The fish and amphibians are found to contain multiple copies of PIP4Kγ with 1–2 aa deletion in this position. More detailed information for this CSI is provided in Figure S7. Other details are the same as in Figure 2 legend. Numbers on the top indicate the position of the sequence in the human PIP4Kγ.

The last of the subfamily-specific CSIs is present in a highly conserved region of the PIP4Kγ, where deletions ranging from 1 aa to 4 aa are present in different groups/classes of the vertebrates (Figure 7). As seen from Figure 7, all PIP4Kγ homologs from mammals and reptiles contain a 3 aa deletion in this region. In the same place, the available homologs of PIP4Kγ from birds harbor a 4 aa deletion while those from the amphibians and fish contain shorter deletions (viz. 1-2 aa) (Figure 7). In contrast, no deletion was present in this region in the PIP4K α and β homologs from vertebrates as well as the PIP4K homologs from other metazoan species. The genetic characteristics of this CSI suggest that the observed variation in the length of this CSI in the vertebrate classes is likely the result of successive genetic changes occurring within the same region at different stages in the evolution of vertebrates. However, the possibility that the observed differences in the lengths of this CSI in the vertebrate lineages are due to independent genetic changes cannot be ruled out. Nonetheless, the observance of these lineage-specific genetic changes within this conserved region of PIP4Kγ suggests that this region plays an important role in determining functional characteristics which are specific for the different classes of vertebrates. Indeed, the functional significance of this region has been reported in a number of recent studies [11,43]. Clarke and Irvine [11] have examined the functional significance of this region with respect to the catalytic activities and substrate interactions of PIP4K isoforms by creating a series of mutations in the PIP4Kγ by replacing the residues of G-loop from the PIP4Kα sequence. The mutant forms of PIP4Kγ containing these changes showed a significant increase in lipid kinase activity [11], suggesting that the evolutionarily conserved indels in this region play an important role in determining the functional differences between different isoforms of PIP4K.

### 3.3. Locations of the Identified CSIs in the Structures of the PIP4K/PIP5K Proteins

**Figure 8:**
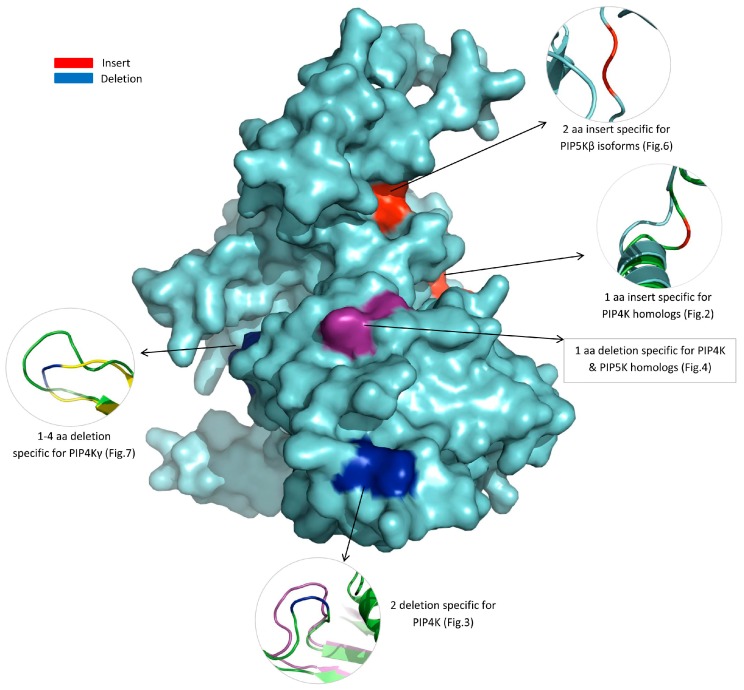
Surface representation of the identified CSIs in a structural model of the human PIP5Kβ protein. For mapping of the CSIs in protein structures, structural information for a number of solved/modeled structures for the PIP4K/PIP5K family of proteins (see Methods section) was utilized. The CSIs which constitute inserts are marked in red on the surface, while for the CSIs that are deletions, the protein regions where these deletions are found are marked in blue on the surface. The location of the 1 aa deletion (Figure 4) that is commonly shared by different homologs of PIP4K and PIP5K is shown in magenta based on structural comparison with the *Saccharomyces cerevisiae* PIP4K/PIP5K homolog. The close-up views of the locations in the protein structure for different identified CSIs are shown in cartoon representation. The structure model of PIP4Kγ isoform from *H. sapiens* is shown in yellow and crystal structure of PIP4Kβ isoform is shown in green.

Earlier work on CSIs in different proteins show that most, if not all, of the previously studied CSIs are located on the surface exposed loops of different proteins, which are important in mediating novel protein-protein or protein-ligands interaction [38,62,63,64,65]. An 8 aa CSI in the PIP5K protein identified in our earlier work, which was specific for the *Saccharomycetaceae* family of fungi, was also located in a surface exposed loop and it was indicated to play an important role in the binding of this protein with membrane surface [33]. In view of these earlier studies, it was of interest to determine the locations in protein structures of the different CSIs identified in the present work. For these studies, structural information that was currently available for different solved structures for the PIP4K/PIP5K family of proteins listed in the Methods section was utilized. In addition, a homology model was also created for the human PIP5Kβ using the solved structure of PIP5Kα from *Danio rerio* (PDB ID: 4TZ7, chain “A”) as a template. In Figure 8, we show a composite diagram, wherein we have mapped the locations of most of the identified CSIs in a structural model of the human PIP5Kβ protein. The CSIs which represent inserts are marked in red whereas CSIs representing deletions are shown in blue. In the zoomed regions in this figure, the structural models showing the locations of different CSIs are shown in cartoon representation. There was no structural information available for the region where the CSI shown in Figure 5 in PIP5Kα is found. Hence, its location in the protein structure was not mapped. As shown in this figure, all identified CSIs in the PIP4K/PIP5K family of proteins are found to be located on the surface exposed loop region and thus they should be able to interact with other proteins/ligands.

## 4. Discussion

The PIP4K/PIP5K family of proteins constitutes crucial players in the regulation of the metabolism of phosphatidylinositides in eukaryotes [21,22,23]. Both PIP4K and PIP5K are involved in generating a key signaling molecule, PI(4,5)P_2_, which resides at the core of the phosphatidylinositol signaling pathway controlling a wide range of fundamental cellular processes [1,3,5]. Further, due to the important roles played by these proteins in many critical processes involved in pathological conditions [66,67,68,69,70,71] these proteins are becoming an increasingly interesting class of molecular targets for cancer [72,73], chronic pain [74], diabetes [75], and autoimmune diseases [76]. However, despite the important roles played by these proteins in the regulation of many cellular processes, our understanding of the overall evolutionary relationships between different members of the PIP4K/PIP5K families and subfamilies of proteins and what specific genetic/biochemical characteristics distinguish different members of this protein family remains limited.

**Figure 9:**
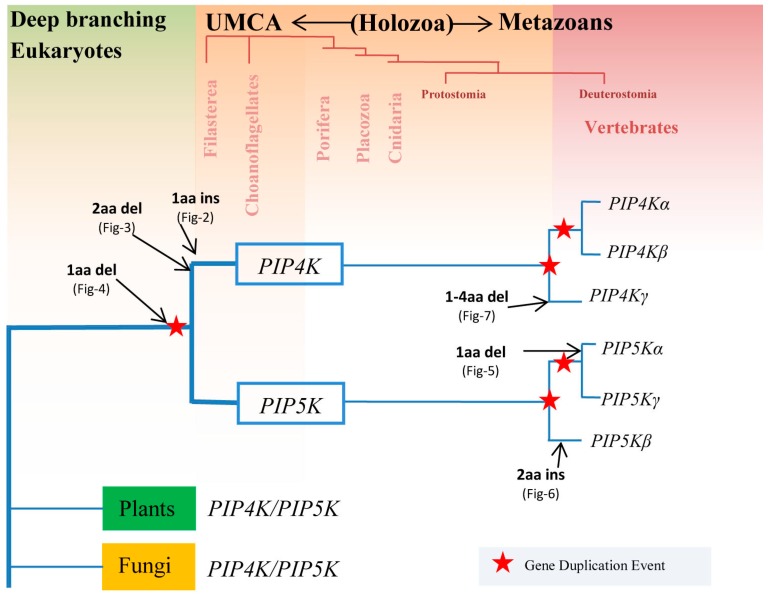
A summary diagram showing the evolutionary divergence of different members of the PIP4K/PIP5K family of proteins in eukaryotic organisms. The model presented here is based on the species distribution of different proteins as well as the species/isozyme specificities of different CSIs in these proteins that were identified in the present work. The arrows mark the evolutionary stages where the rare genetic changes leading to the specific CSI(s) are postulated to have occurred. The red stars mark the evolutionary stages where gene duplication events have occurred during the divergence of this protein family. Holozoa clade comprises of the multicellular metazoan phyla as well as phyla consisting of their unicellular metazoan common ancestor (UMCA).

The present study was undertaken with the aims of advancing our understanding of the evolutionary divergence of different members of the PIP4K/PIP5K protein families from a common ancestor and also to identify novel molecular features in these proteins that are distinctive characteristics of a particular family or subfamily of these proteins. Our analyses of protein sequences from the PIP4K/PIP5K reported here have identified six highly-specific molecular signatures in the forms of CSIs that are distinctive characteristics of specific isozymes and subfamilies of these proteins. Based on the results obtained from the species distributions of PIP4K/PIP5K isozymes/homologs in different eukaryotic lineages, phylogenetic analysis based on the sequences of these proteins, and the inferences derived from the species and isozyme specificities of different identified CSIs in these proteins, several novel and important insights regarding the evolutionary divergence of this protein family can be gleaned. An overall summary of the results obtained from different approaches regarding the evolutionary divergence of the PIP4K/PIP5K family of proteins is presented in Figure 9. In addition to showing the distribution of different members of the PIP4K/PIP5K family of proteins in eukaryotic lineages, this figure also marks the evolutionary stages where the genetic changes responsible for different identified CSIs likely occurred during the evolution of this protein family.

As shown here, the distinct homologs of both PIP4K and PIP5K, in addition to the Bilateria species [4], are also found in other multicellular metazoan phyla (viz Cnidaria, Placozoa, and Porifera) as well as in the Choanoflagellates and Filasterea, which are the closest-known unicellular ancestor of the multicellular animals. These groups together form the Holozoa clade of species [59,60]. In contrast, all other deeper branching eukaryotic lineages including Apicompelxa, Amebozoa, Percolzoa, and Apusozoa, as well as plants and fungi, contain only a single homolog of these proteins showing similarity to both PIP4K/PIP5K. Our work has identified a conserved indel that is commonly shared by all PIP4K and PIP5K homologs from Holozoa species (except some PIP4K homologs from nematodes, whose significance is unclear) but absent in the single copy homologs of these proteins found in the deeper branching lineages (Figure 4). Based on the species and isozyme distribution of this CSI, the genetic change leading to this CSI has occurred in a common ancestor of the Holozoa clade prior to the first gene duplication event leading to the divergence of distinct types of PIP5K and PIP4K homologs. These results indicate that this genetic change preceded the evolution of all animals (i.e., both unicellular and multicellular). Two other CSIs identified in this study are uniquely found in all PIP4K homologs (Figure 2 and Figure 3) but they not present in any PIP5K homolog. The genetic changes giving rise to these CSIs are postulated to have occurred in a common ancestor of the PIP4K homologs soon after the gene duplication event leading to the formation of distinct forms of the PIP4K and PIP5K proteins. Due to the specificities of these CSIs for either the PIP4K/PIP5K proteins or only the PIP4K isozymes, these molecular markers provide useful means for genetic and biochemical studies leading to the discovery of novel and distinctive properties of these isozymes.

The vertebrate species contain three different subfamilies of PIP4K and PIP5K proteins. In phylogenetic trees, all three subfamilies of the PIP4K are part of one distinct clade whereas the different members of the PIP5K subfamilies form a separate clade. As noted in earlier work [4], the branching patterns of these proteins strongly suggest that the members of these subfamilies have originated from two independent and successive duplications of the genes for PIP4K and PIP5K proteins within vertebrates [4]. As all three forms (or isoforms) of PIP4K and PIP5K proteins are present in different (or most) vertebrate species, the gene duplication events leading to the divergence of these protein families have occurred in a vertebrates’ common ancestor within an evolutionarily short period. Due to this, the relative branching orders of these protein families can be inferred only tentatively. Work on the evolution of the phosphatidylinositol-3-kinases family of protein kinases indicates that the gene for the catalytic subunit of this protein has also undergone two major duplication events at different stages in the evolution of eukaryotic organisms to account for its species distribution [77].

It is important to understand how different isoforms of the PIP4K and PIP5K proteins differ from each other and what unique features distinguish these isoforms. In this context, our identification of several CSIs that are distinctive characteristics of the specific subfamilies of either PIP4K or PIP5K proteins, or both, is of much interest. Of the three subfamily-specific CSIs identified in this work, one is specific for all PIP5Kα homologs. The genetic change responsible for this CSI likely occurred in a common ancestor of the PIP5Kα subfamily, when it diverged from the PIP5Kβ-PIP5Kγ by a gene duplication event. Another CSI described here is specific for the PIP5Kβ homologs from mammals, birds, and reptiles (Figure 6). The genetic change leading to this CSI likely occurred in a common ancestor of the PIP5Kβ protein from mammals, birds, and reptiles and it supports the latter divergence of these vertebrate classes in comparison to the fish and amphibians [61]. In addition to these CSIs, within a highly conserved region of the PIP4Kγ subfamily of proteins, deletions of specific lengths are present in different groups/classes of vertebrate species (Figure 7). The observed variation in the length of this CSI in vertebrate groups suggests that successive genetic changes have occurred in this position during the evolution of vertebrates, indicating that this region should be of particular importance in the functioning of this protein. The genetic changes leading to these isoforms or subfamilies-specific CSIs have occurred at important stages in the evolution of vertebrate species and the identified molecular signatures provide important means for distinguishing some of these distinct isoforms and understanding their unique functional characteristics.

As has been noted earlier, the CSIs in genes/proteins sequences result from rare genetic changes and these changes have been found to be important/essential for the proper functioning of the proteins in the CSI-containing organisms [33,38,78]. Further, most studied CSIs, including those identified in this study, are located in surface-exposed loops of the proteins which, due to their ability to interact with other proteins and ligands, perform important roles in mediating novel functional interactions [33,38,62,63,64,79]. Previously, we have reported the identification of an 8 aa CSI in the PIP5K homologs which was specific for the *Saccharomycetaceae* family of fungi. This CSI formed a positively-charged patch on the surface of the protein and it was predicted to play a role in the binding of the yeast PIP5K protein with the membrane. This prediction was strongly supported by molecular dynamics simulation studies examining the binding interaction of the yeast PIP5K protein, with and without the CSI, with the membrane lipid bilayers. Clarke and Irvine [11] have examined the functional significance of the region where the 1–4 aa deletions are present in the PIP4Kγ isoforms in different vertebrate species. Their study also showed that the changes in the residues corresponding to the identified CSIs significantly affected the functional activity of the protein and could account for the differences in the functional activity of the different isoforms [11]. Thus, it is strongly expected that the other CSIs identified in this study in the PIP4K/PIP5K family of proteins should also be playing important roles in the cellular functions of these proteins and the described CSIs provide novel genetic and biochemical means to investigate such differences. Lastly, due to the important roles played by the PIP4K/PIP5K family of proteins in different cellular processes associated with a variety of pathological conditions (e.g., cancer, diabetes, chronic pain, autoimmune diseases) [66,67,68,69,70,71], the identified CSIs in these proteins, which are surface exposed and predicted to play important cellular functions, also provide potential means for development of novel therapeutics targeting specific diseases [72,73,74,75,76].

## Figures and Tables

**Table 1:** Distribution of PIP4K/PIP5K family of proteins in the major groups of eukaryotes.

		Taxa/Phylum	PIP4K	PIP5K	PIP4K/PIP5K
DEUTEROSTOMIA	Vertebrates	Mammals	>100	>100	-
		Birds	>100	>100	-
		Amphibians	4	4	-
		Reptiles	10	10	-
		Fishes	>50	>50	-
		Tunicata	2	2	-
		Cephalochordata	2	2	-
		Hemichordata	1	1	-
		Echinodermata	1	1	-
PROTOSTOMIA		Arthropoda	>100	>100	-
		Nematoda	>50	>50	-
		Mollusca	4	4	-
		Annelida	2	2	-
		Platyhelminthes	2	2	-
		Tardigrada	2	2	-
Early Metazoans		PLACOZOA	1	1	-
		PORIFERA	1	1	-
		CNIDARIA	3	3	-
UMCA		CHOANOFLAGELLATEA	2	2	-
		FILASTEREA	1	1	-
		ICHTHYOSPOREA	1 *	1 *	-
		FUNGI	-	-	>100
		PLANTS	-	-	>100
OTHERS EUKARYOTES		APICOMPLEXA	-	-	>10
		AMOEBOZOA	-	-	
		PERCOLOZOA (*Naegleria gruberi*)	-	-	
		APUSOZOA (*Thecamonas trahens*)	-	-	

* 1 hit showing low sequence conservation was observed. The numbers in different columns indicate the number of species from the indicated groups for which sequence information was available.

## Supplementary Materials

The following are available online at https://www.mdpi.com/2073-4425/10/4/312/s1, Table S1: Sequence information for different PIP4K/PIP5K family of protein sequences used in phylogenetic studies, Figure S2: detailed species distribution information for the 1 aa CSI in PIP4K shown in Figure 2, Figure S3: detailed species distribution information for the 2 aa deletion in PIP4K in Figure 3, Figure S4:; detailed species distribution information for the 1 aa CSI shown in Figure 4, Figure S5: detailed species distribution information for the 1 aa conserved deletion in PIP5Kα shown in Figure 5, Figure S6: detailed species distribution information for the 2 aa conserved insert in PIP5Kβ isoform shown in Figure 6, Figure S7: detailed species distribution information for the 1–4 aa conserved deletions in PIP4Kγ isoforms shown in Figure 7.
