# Supplementary material for: Novel Molecular Signatures in the PIP4K/PIP5K Family of Proteins Specific for Different Isozymes and Subfamilies Provide Important Insights into the Evolutionary Divergence of this Protein Family
Source: Genes (Basel). 2019 Apr 21;10(4):312. doi: 10.3390/genes10040312 (PMC6523245; doi:10.3390/genes10040312)
Supplement: Supplementary file 1 [file genes-10-00312-s001.pdf]

Table S1. Sequence information for different PIP4K/PIP5K family of protein sequences used in phylogenetic studies.

Organism Name	Accession Number	Protein Name	Protein Length
Homo sapiens	NP_003550.1	PIP4Kβ	416
Serinus canaria	XP_009094714.2	PIP4Kβ	442
Xenopus tropicalis	XP_002940195.1	PIP4Kβ	418
Protobothrops mucrosquamatus	XP_015670248.1	PIP4Kβ	422
Maylandia zebra	XP_004538792.1	PIP4Kβ	412
Homo sapiens	XP_011523628.1	PIP4Kα	443
Serinus canaria	XP_009084143.1	PIP4Kα	347
Protobothrops mucrosquamatus	XP_015667052.1	PIP4Kα	404
Maylandia zebra	XP_004546610.1	PIP4Kα	404
Xenopus tropicalis	NP_001123723.1	PIP4Kα	405
Homo sapiens	AAC32904.1	PIP4Kγ	461
Protobothrops mucrosquamatus	XP_015679464.1	PIP4Kγ	402
Sturnus vulgaris	XP_014747327.1	PIP4Kγ	344
Maylandia zebra	XP_004560306.1	PIP4Kγ	416
Xenopus tropicalis	XP_017946647.1	PIP4Kγ	450
Branchiostoma floridae	XP_002599487.1	PIP4K	408
Apostichopus japonicus	PIK54083.1	PIP4K	398
Saccoglossus kowalevskii	XP_002732674.1	PIP4K	406
Mizuhopecten yessoensis	XP_021377781.1	PIP4K	407
Crassostrea virginica	XP_022305822.1	PIP4K	412
Capitella teleta	ELU08768.1	PIP4K	399
Helobdella robusta	XP_009023323.1	PIP4K	403
Drosophila melanogaster	NP_001033805.1	PIP4K	404
Apis mellifera	XP_392797.3	PIP4K	430
Hypsibius dujardini	OQV16814.1	PIP4K	396
Exaiptasia pallida	XP_020903371.1	PIP4K	437
Hydra vulgaris	XP_002161268.1	PIP4K	405
Trichoplax adhaerens	XP_002111279.1	PIP4K	393
Amphimedon queenslandica	XP_019863884.1	PIP4K	469
Ciona intestinalis	XP_002119441.3	PIP4K	424
Trichinella britovi	KRY60612.1	PIP4K	445
Caenorhabditis elegans	NP_497500.1	PIP4K	401
Loa loa	XP_020305842.1	PIP4K	412
Brugia malayi	XP_001902899.1	PIP4K	412
Macrostomum lignano	PAA91900.1	PIP4K	440
Clonorchis sinensis	GAA55841.1	PIP4K	499
Capsaspora owczarzaki	XP_004364933.1	PIP4K	349
Monosiga brevicollis	EDQ89588.1	PIP4K	353
Salpingoeca rosetta	XP_004998565.1	PIP4K	357
Homo sapiens	XP_011526147.1	PIP5Kγ	731
Xenopus tropicalis	XP_017946547.1	PIP5Kγ	675
Protobothrops mucrosquamatus	XP_015666168.1	PIP5Kγ	708
Serinus canaria	XP_018777319.1	PIP5Kγ	664
Maylandia zebra	XP_012772285.1	PIP4Kγ	819
Maylandia zebra	XP_004541373.2	PIP5Kα	610

Protobothrops mucrosquamatus	XP_015680374.1	PIP5Kα	989
Serinus canaria	XP_018781091.1	PIP5Kα	639
Homo sapiens	NP_001129108.1	PIP5Kα	522
Maylandia zebra	XP_004538536.1	PIP5Kβ	498
Xenopus tropicalis	XP_004910836.1	PIP5Kβ	539
Protobothrops mucrosquamatus	XP_015679138.1	PIP5Kβ	540
Serinus canaria	XP_009092185.1	PIP5Kβ	540
Homo sapiens	AAH30587.1	PIP5Kβ	549
Ciona intestinalis	XP_018673474.1	PIP5K	641
Branchiostoma floridae	XP_002591361.1	PIP5K	385
Apostichopus japonicus	PIK60516.1	PIP5K	542
Saccoglossus kowalevskii	XP_006821157.1	PIP5K	588
Mizuhopecten yessoensis	XP_021350909.1	PIP5K	804
Crassostrea virginica	XP_022306935.1	PIP5K	583
Helobdella robusta	XP_009026309.1	PIP5K	453
Capitella teleta	ELT87243.1	PIP5K	997
Drosophila melanogaster	NP_611729.2	PIP5K	729
Apis mellifera	XP_006571145.1	PIP5K	747
Caenorhabditis elegans	NP_491576.2	PIP5K	611
Loa loa	XP_020303847.1	PIP5K	568
Brugia malayi	CDP97235.1	PIP5K	530
Hypsibius dujardini	OQV12309.1	PIP5K	656
Exaiptasia pallida	XP_020899131.1	PIP5K	632
Hydra vulgaris	XP_012564577.1	PIP5K	336
Clonorchis sinensis	GAA56497.1	PIP5K	1636
Macrostomum lignano	PAA67906.1	PIP5K	577
Trichoplax adhaerens	XP_002108154.1	PIP5K	369
Amphimedon queenslandica	XP_019849193.1	PIP5K	618
Capsaspora owczarzaki	XP_004348939.1	PIP5K	733
Monosiga brevicollis	EDQ89244.1	PIP5K	1081
Salpingoeca rosetta	XP_004997164.1	PIP5K	1006
Glycine max	XP_006584672.1	PIP4K/PIP5K	719
Arabidopsis thaliana	AAB65487.1	PIP4K/PIP5K	859
Oryza sativa	EEC76033.1	PIP4K/PIP5K	731
Coffea canephora	CDP17283.1	PIP4K/PIP5K	772
Candida albicans	KGQ92052.1	PIP4K/PIP5K	721
Clavispora lusitaniae	XP_002615347.1	PIP4K/PIP5K	655
Kluyveromyces lactis	XP_451188.1	PIP4K/PIP5K	719
Saccharomyces cerevisiae	NP_010494.1	PIP4K/PIP5K	779
Aspergillus nidulans	XP_660370.1	PIP4K/PIP5K	841
Fonticula alba	XP_009497027.1	PIP4K/PIP5K	934
Ectocarpus siliculosus	CBJ28352.1	PIP4K/PIP5K	481
Phytophthora infestans	XP_002897706.1	PIP4K/PIP5K	651
Saprolegnia parasitica	XP_012202766.1	PIP4K/PIP5K	539
Plasmodium vivax	KMZ89198.1	PIP4K/PIP5K	1505
Toxoplasma gondii	EPT29018.1	PIP4K/PIP5K	1293
Leishmania infantum	XP_001468654.1	PIP4K/PIP5K	546

Naegleria gruberi	XP_002670454.1	PIP4K/PIP5K	650
Tieghemostelium lacteum	KYQ96762.1	PIP4K/PIP5K	636
Cavenderia fasciculata	XP_004359026.1	PIP4K/PIP5K	661
Polysphondylium pallidum	XP_020433305.1	PIP4K/PIP5K	674

Figure S2. Detailed species distribution information for the 1 aa CSI in PIP4K shown in Figure 2.

Fungi		493	533
Saccharomyces cerevisiae	NP_010494.1	YIIKTIHHSEHIHLRKHIQEYYNHVRDNP	NTLICQFYGLHR
Candida glabrata	XP_446202.1	-------------------------KT--	D-----------
Zygosaccharomyces rouxii	XP_002495864.1	--------T--V---R-LN---L--KN--	D-----------
Kluyveromyces lactis	XP_451188.1	-----------K----ILKR--E--KQ--	D---S-------
Candida albicans	KGU13027.1	F----------KQ-LRMLKD-HH--K---	----S-------
Pleurotus ostreatus	KDQ31819.1	F-----RKE-ATF--NVLKQ--D--KT--	H--VSR------
Lichtheimia corymbifera	CDH54623.1	F-------T--RF---VLKD-----CQ--	---LSR------
Schizosaccharomyces pombe	BAA87265.1	F----------KF--EILYD--E--KN--	----S-------
Pneumocystis jirovecii	CCJ29873.1	-----------KF---ILKQ--E--KK--	----S-------
Taphrina deformans	CCG81999.1	F----L--A--KF--RILPD-HD-TKKY-	----S-------
Sporidiobolus salmonicolor	CEQ39140.1	F-----R-A--KF--SILK--HEY-MK--	H---SR------
Malassezia globosa	XP_001732363.1	F-----RPT--KLFI-FLPA--E---A--	H--LS-------
Rhizophagus irregularis	ESA19204.1	F-------T--KF---ILK---E-I-N--	D--LSR------
Mortierella verticillata	KFH71086.1	F-------A--KFM--ILKD-F---KQ--	H--L-R-F----
Batrachochytrium dendrobatidis	XP_006675062.1	F----------KFIL-ILEY----I-S--	H--LSRIF----
Kazachstania naganishii	XP_022462372.1	---------------R-V--------E--	------------
Naumovozyma castellii	XP_003674750.1	-----------R---R-L----K--KA--	------------
Tetrapisispora blattae	XP_004182131.1	--------A------R-LK---L-IKN--	---V--------
Vanderwaltozyma polyspora	XP_001643104.1	-----------L-----LND--K--KE--	D--L--------
Torulaspora delbrueckii	XP_003682564.1	--------A--V---R-LHA--T-IKE--	D-M---------
Lachancea nothofagi	SCV03977.1	--------A--Q---RTLK---S--KE--	----S-------
Leptosphaeria maculans	XP_003841630.1	--------A--KF---ILKD-----QE--	---LS-------
Capronia epimyces	XP_007737551.1	--------A--KL---ILRD--R--V---	----S-------
Fonsecaea multimorphosa	XP_016637750.1	--------A--KL---ILRD--R--L---	----S-------
Cladophialophora bantiana	XP_016624332.1	--------A--KL---ILRD--R--L---	----S-------
Aureobasidium melanogenum	KEQ67430.1	--------A--KF---ILRD--S--Q---	---LS-------
Tuber aestivum	CUS11234.1	--------G--LF---ILRD-WK--E---	D---S-------
Exophiala oligosperma	XP_016268534.1	--------A--KL--RILRD--R--V---	----S-------
Pyrenophora teres	EFQ90510.1	--------A--KF---ILKD-----QE--	---LS-------
Endocarpon pusillum	XP_007803590.1	--------A--KL---ILR---K--I---	D---S-------
Coniosporium apollinis	XP_007784276.1	--------A--KF---ILRD--D---A--	D--LS-------
Paraphaeosphaeria sporulosa	XP_018032922.1	--------G--KF---ILKD-----QE--	---LS-------
Phialophora attae	XP_018004907.1	--------A--KL--RILR---H--K---	---MS-I-----
Cyphellophora europaea	XP_008713371.1	--------A--KL--RILRD-H---LE--	----S-I-----
Wickerhamomyces ciferrii	XP_011276550.1	F------PA--R---RMLKD-H---KN--	---VS-------
Pyronema omphalodes	CCX06330.1	--------G--RF--HILRD--Q-IQE--	D---S-------
Cyberlindnera jadinii	CEP23152.1	F------YA--RQ--RILKR--E--KS--	----S-------
Plants			
Zea mays	NP_001148043.1	FM---LRK--VQV-LRMLP---H---TYE	----TK-F----
Solanum tuberosum	XP_006349377.1	FV---LKK--LKV-L-MLPD--K--KEHD	----TKVF-V--
Glycine max	XP_006597317.1	FV---VNK--LKV-LSMLPK--R--G-HE	----TK-F----
Oryza sativa Japonica Group	NP_001173424.1	-M---MKK--VKM-L-MLPA------AFE	D--VTK-F---C
Coffea canephora	CDP17283.1	-M---MKKA-VKV-LRMLPA------AFE	----AK-----C
Musa acuminata	XP_018675993.1	-M---MKK--VKV-LRMLPA------AFE	---VTK-F---C
Elaeis guineensis	XP_019706081.1	-M---MKK--VKV-LRMLPA------AFE	---VTK-F---C
Aquilegia coerulea	PIA60189.1	-M---MKK--VKV-LRMLPA------AFE	---VTK-F---C
Apostasia shenzhenica	PKA53367.1	-M---MKK--VKV-LRMLPA------AFE	---VTK-F---C
Nelumbo nucifera	XP_010276643.1	-M---MKK--VKV-IRMLPA------AFE	---VTK-F---C
Fragaria vesca subsp. vesca	XP_004307106.1	-M---MKKA-VKV-LRMLPA------AFE	---VTK-F---C
Macleaya cordata	OVA18495.1	-M---MKK--VKV-LRMLPA------AFE	---VTK-----C
Ipomoea nil	XP_019151708.1	-M---MKK--VKVFIRMLPA------SFE	---VTK-F---C
Malus domestica	XP_008375376.1	-M---MKKA-VKV-LRMLPA------SFE	---VTK-----C
Juglans regia	XP_018849425.1	-M---MKKA-VKV-LRMLPA--K---AFE	---VTK-----C
Dorcoceras hygrometricum	KZV47292.1	-M---MKK--VKV-KRMLPA--K---AFE	---VTK-F---C
Morus notabilis	EXB62690.1	-M---MKKA-VKV-LRMLPA--K---AFE	---VTK-----C
Cajanus cajan	XP_020240276.1	-M---MKK--VKVFLRMLPG--K---AFE	---VTR-F---C
Parasponia andersonii	PON53305.1	-M---MKKA-VKV-LRMLPA--K---AFE	---VTK-----C
Solanum lycopersicum	XP_004230026.1	-M---LKKA-VKV-LGMLPA------AFE	---VTK-F---C
Capsicum annuum	XP_016557282.1	-M---LKKA-VKV-LGMLPA------AFE	---VTK-F---C
Trema orientalis	PON98771.1	-M---MKKA-VKV-LRMLPA--K---AFE	---VTK-----C
Vitis vinifera	XP_002267007.3	-M---MKK--VKV-LRMLSA------AFE	---VTK-----C
Trifolium pratense	PNY15056.1	-M---MKKA-VKVFI-MLPA--K---AFE	---VTK-F---C
Ricinus communis	XP_002526916.1	-M---MKKA-VKV-IRMLAA------AFE	---VTK-F---C
Hevea brasiliensis	XP_021677612.1	-M---MKKA-VKV-IRMLAA------VFE	---VTK-F---C
Panicum hallii	PAN49615.1	-M---MKKA-VKL-L-MLPA------AFE	D--VTK-F---C

Cephalotus follicularis	GAV61641.1	-M---MKK--VKV-IRMLSA------AFE	---VTK-----C
Pyrus x bretschneideri	XP_009372761.1	-M---MKKA-VKV-LRMLRA------SFE	---VTK-----C
Ziziphus jujuba	XP_015902998.1	-M---MKKA-VKV-LRMLSA------AFE	---VTK-----C
Carica papaya	XP_021908574.1	-M---MKK--VKV-LRMLSA------AFE	---VIK-----C
Manihot esculenta	XP_021624297.1	-M---VKKA-VKM-IRMLAA------AFE	---VTK-F---C
Sorghum bicolor	OQU92574.1	-M---MKKA-VKM-LRML-A------AFE	D--VTK-F---C
Eucalyptus grandis	KCW90045.1	-M----KKA-VKV-LRMLSA----F-AFE	---VTK-----C
Daucus carota subsp. sativus	XP_017254749.1	-M----KKA-VKV-KRMLPA--S---ACE	---VTT-F---C
Brassica napus	XP_013721154.2	-M---MKK--TKV-LRMLAA------AFE	---VIR-----C
Camelina sativa	XP_010427550.1	-M---MKK--TKV-LRMLAA------AFE	---VIR-----C
Capsella rubella	XP_023638231.1	-M---MKK--TKV-LRMLAA------AFE	---VIR-----C
Eutrema salsugineum	ESQ44381.1	-M---MKK--TKV-LRMLAA------AFE	---VIR-----C
Raphanus sativus	XP_018489538.1	-M---MKK--TKVFLRMLAA------AFE	---VIR-----C
Genlisea aurea	EPS66188.1	-M---MKKV-TKV-LRMLNA------AFE	C--VTKYF---C

Vertebrates PIP5K (>100)

Homo sapiens-Alpha-5K	NP_001129108.1	F----VQ-K-AEF-Q-LLPG--MNLNQ--	R--LPK----YC
Serinus canaria-Alpha-5K	XP_018781091.1	F----VQ-K-AEF-Q-LLPG-FMNINQ-K	R--LPK----YC
Python bivittatus-Alpha-5K	XP_015744820.1	F----VQ-K-AEF-Q-LLPG--MNLNQ--	R--LPK----YC
Protobothrops mucrosquamatus-Alpha-5K	XP_015680374.1	F----VQ-K-AEF-Q-LLPG--MNLNQ--	R--LPK----YC
Xenopus tropicalis-Alpha-5K	NP_001006899.1	F----VQ-K-AEF-Q-LLPG--MNLNQ--	R--LPK----YC
Maylandia zebra-Alpha-5K	XP_004541373.2	F----VQ-K-AEF-Q-LLPG-FMNINQ-K	R--LPK----YC
Pundamilia nyererei-Alpha-5K	XP_005740686.1	-----VQ-K-AEF-Q-LLPG-FMNLNQ-K	R--LPK----YC
Homo sapiens-Beta-5K	NP_003549.1	F----VQ-K-AEF-Q-LLPG--MNLNQ--	R--LPK----YC
Serinus canaria-Beta-5K	XP_009092185.1	F----VQ-K-AEF-Q-LLPG--MNLNQ--	R--LPK----YC
Protobothrops mucrosquamatus-Beta-5K	XP_015679138.1	F----VQ-K-AEF-Q-LLPG--MNLNQ--	R--LPK----YC
Xenopus tropicalis-Beta-5K	XP_004910836.1	F----VQ-K-AEF-Q-LLPG--MNLNQ--	R--LPK----YC
Maylandia zebra-Beta-5K	XP_004538536.1	F----VQPK-AEF-Q-LLPG--MNLNQ--	R--LPK----YC
Pundamilia nyererei-Beta-5K	XP_005725614.1	F----VQPK-AEF-Q-LLPG--MNLNQ--	R--LPK----YC
Homo sapiens-gamma-5K	XP_011526147.1	F----VM-K-AEF-Q-LLPG--MNLNQ--	R--LPK----YC
Sturnus vulgaris-gamma-5K	XP_014739865.1	F----VM-K-AEF-Q-LLPG--MNLNQ--	R--LPK----YC
Serinus canaria-gamma-5K	XP_018777319.1	F----VM-K-AEF-Q-LLPG--MNLNQ--	R--LPK----YC
Protobothrops mucrosquamatus-gamma-5K	XP_015666168.1	F----VM-K-AEF-Q-LLPG--MNLNQ--	R--LPK----YC
Xenopus tropicalis-gamma-5K	XP_017946547.1	F----VM-K-AEF-Q-LLPG--MNLNQ--	R--LPK----YC
Pundamilia nyererei-gamma-5K	XP_005721178.1	F----VL-K-AEF-Q-LLPG--MNLNQ--	R--LPK-F--YC
Maylandia zebra-gamma-5K	XP_012772288.1	F----VL-K-AEF-Q-LLPG--MNLNQ--	R--LPK-F--YC

Tunicata

Ciona intestinalis-5K	XP_018673474.1	F----VQ-K-AEF-Q-LLPG--MNLVQ--	R--LPK----YN
Oikopleura dioica-5K	CBY09966.1	F-V--VQQK-ASF-T-LLPA-FMA-HQ--	K--LPK----FN

Cephalochordata/ Amphioxus

Branchiostoma floridae-5K	XP_002591361.1	F-V--VQ-K-ADF-Q-LLPG--MNLNQ--	R--LPK-F--YC

Echinodermata

Apostichopus japonicus-5K	PIK49174.1	F----VQ-K-ADF-Q-LLPG--MNLNQ--	R--LPK----YT

Hemichordata

Saccoglossus kowalevskii-5K	XP_006821157.1	F----VQ-K-ADF-Q-LLPG--MNLNQ--	R--LPK----YT

PROTEOSOME

Arthropoda

Apis mellifera-5K	XP_006571145.1	F----VQ-K-GEF-QTLLPG--MNLNQ--	R--LPK-F--YC
Drosophila melanogaster-5K	NP_611729.2	F----VQ-K-GEF-Q-LLPG--MNLNQ--	R--LPK-F--YC

Nematoda

Trichinella britovi-5K	KRY55394.1	F-V--VQ-R-AEF-Q-LLPG--MNLNQ--	K--LPK-F-FYC
Trichinella spiralis-5K	KRY35256.1	F-V--VQ-R-AEF-Q-LLPG--MNLNQ--	K--LPK-F-FYC
Trichinella papuae-5K	KRZ69085.1	F-V--VQ-K-AEF-Q-LLPG--MNLNQ--	K--LPK-F-FYC
Caenorhabditis brenneri-5K	EGT37979.1	F----VQ-K-ADF-Q-LLPG--MNLNQ--	R--LPK-F--FC
Caenorhabditis elegans-5K	NP_491576.2	F----VQ-K-ADF-Q-LLPG--MNLNQ--	R--LPK-F--FC
Loa loa-5K	XP_020303847.1	F----VQSK-AEF-K-LLPG--MNFNQ--	H--LPK-F--FC
Wuchereria bancrofti-5K	EJW88844.1	F----VQSK-AEF-K-LLPG--MNFNQ--	H--LPK-F--FC
Brugia malayi-5K	CDP97235.1	F----VQSK-AEF-K-LLPG--MNFNQ--	H--LPK-F--FC
Strongyloides ratti-5K	CEF65455.1	F----VQYK-AEF--ELLPG--MNICQ--	K-FLPK-F--FC

Mollusca

Biomphalaria glabrata-beta-5K	XP_013074799.1	F----VQ-K-AEF-Q-LLPG-FLNISQ-K	R--LPK----YC
Octopus bimaculoides-alpha-5K	XP_014789196.1	F----VQ-K-AEF-Q-LLPG--LNLNQ--	R--LPK----YC
Crassostrea virginica-5K	XP_022306935.1	F----VQ-K-AEF-Q-LLPG--MNLNQ--	R--LPK----YC
Mizuhopecten yessoensis-5K	XP_021350909.1	F----VQ-K-ADF-Q-LLPG--MNLNQ--	R--LPK----YC

Annelida

Helobdella robusta-5K	XP_009026309.1	F----VQRK-ADF-Q-LLPG--MNLNQ--	R--LPK----YC
Capitella teleta-5K	ELT87243.1	F----VQ-K-AEF-Q-LLPG--MNLNQ--	R--LPK----YC

Platyhelminthes

Macrostomum lignano-5K	PAA67906.1	F----VQ-K-AKY-QRLLLQ--LTLTQ--	R--LPK----YC
Clonorchis sinensis-5K	GAA56497.1	F----VQ-R-GEF--ALLPS-FMNLWQH-	P--LPK-H-FYC

**Tardigrada**				
Hypsibius dujardini-5K	OQV12309.1	F-L--VMYR-AEF-Q-LLAG--MNLNQ--		R--LPK-F-QYC
Ramazzottius varieornatus-5K	GAU91082.1	F----VD---AGF-L-LLPQ--MNIVQ--		R--LPK-F--YC
**PLACOZOA**				
Trichoplax adhaerens-5K	XP_002108154.1	F----VQKK-AQF-QELLPG--LNFSQ-K		K--LPK-F--YS
**PORIFERA**				
Amphimedon queenslandica-5K	XP_019849193.1	F----VQKK-AVF-Q-LLPG--LNLTQ-K		R--LPK-F--YQ
**CNIDARIA**				
Hydra vulgaris-5K	XP_012564577.1	F-V--VT-K-ATF-QQLLPG--MNLHQ-A		R--LPK-F--YC
Exaiptasia pallida-5K	XP_020899131.1	F----VD-K-AEF-Q-LLPG--LNLNQ-K		K--LPK-F-QFC
Nematostella vectensis-5K	XP_001633067.1	F----VQ-K-AEF-Q-LLPG--LNLNQ-K		R--LPK-F--FC
**CHAENOFLAGELLATA**				
Salpingoeca rosetta-5K	XP_004997164.1	F-V---QKG--KF-T-LLPQ--LNLHQ-K		R--LPK-FAHFC
Monosiga brevicollis-5K	EDQ89244.1	F-V--VQKG-SKF---LLPA--LNITQ-Q		R--LPK-F-HFC
**FILASTEREA**				
Capsaspora owczarzaki-5K	XP_004348939.1	F----VQRR-ALF--QLLPG--MNLTQ-K		K--LPK-F--YC
**Vertebrates PIP4K (>100)**				
Homo sapiens-Alpha-4K	NP_005019.2	------TSEDVAEMHNILKK-HQYIVECH	G	I--LP--L-MY-
Serinus canaria-Alpha-4K	XP_009084143.1	------TSEDVAEMHNILKK-HQFIVECH	G	---LP--L-MY-
Python bivittatus-Alpha-4K	XP_007436413.1	-V----TSEDVAEMHNILKK-HQYIVECH	G	---LP--L-MY-
Xenopus tropicalis-Alpha-4K	NP_001123723.1	------TSEDVAEMHNILKK-HQFIVECH	G	---LP--L-MY-
Protobothrops mucrosquamatus-Alpha-4K	XP_015667052.1	-V----TSEDVAEMHNILKK-HQFIVECH	G	---LP--L-MY-
Maylandia zebra-Alpha-4K	XP_004546610.1	-V----SSEDVAEMHNILKK-HQFIVECH	G	---LP--L-MY-
Homo sapiens-Beta-4K	EAW60533.1	FV---VSSEDVAEMHNILKK-HQFIVECH	G	---LP--L-IST
Serinus canaria-Beta-4K	XP_009094714.2	FV--AVSSEDVAEMHNILKK-HQFIVECH	G	---LP--L-MY-
Python bivittatus-Beta-4K	XP_007429905.1	FV--AVSSEDVAEMHNILKK-HQFIVECH	G	---LP--L-MY-
Xenopus tropicalis-Beta-4K	XP_002940195.1	FV----SSEDVAEMHNILKK-HQFIVECH	G	---LP--L-MY-
Maylandia zebra-Beta-4K	XP_004538792.1	FV---VSSEDIAEMHNILKK-HQFIVECH	G	---LP--L-MY-
Homo sapiens-Gamma-4K	XP_011537049.1	LV--EVSSEDIADMHSNLSN-HQYIVKCH	G	---LP--L-MY-
Sturnus vulgaris-Gamma-4K	XP_014747327.1	LVL-ELSSEDVADVHGLLSH-HQY-VQCH	G	Q--LPR-L-MY-
Python bivittatus-Gamma-4K	XP_007422116.1	VV--E-TSEDVADVHSLLSH-HQYIVKCH	G	S--LP--L-MY-
Xenopus tropicalis-Gamma-4K	XP_017946647.1	LV--E-SSEDVADMHNILSH-HQ-IVKCH	G	---LP--L-MY-
Maylandia zebra-Gamma-4K	XP_004560306.1	LVV-Q-SSEDVADMHNILS--HQ-IVKCH	G	---LP--L-MY-
Protobothrops mucrosquamatus-Gamma-4K	XP_015679464.1	VV--E-TSEDVADVHSLLSH-HQYIVKCH	G	S--LP--LSMY-
Pundamilia nyererei-Gamma-4K	XP_005739600.1	LV--E-SSE-VAEMHNALSA-HQ-IVTCH	G	S--LP--LAMY-
**Tunicata**				
Ciona intestinalis-4K	XP_002119441.3	-V---LNGEDIAEMHGLLPK-HQYIVEHN	S	K--LP-YL-MY-
Oikopleura dioica-4K	CBY18232.1	F-VSL-STEDVEQ-HNVLPS--A--VEQK	G	E--LPNWL--Y-
**Cephalochordates/ Amphioxus**				
Branchiostoma floridae-4K	XP_002599487.1	-V----ESE-VAQMHHLLKQ-HQ-IVE-H	S	E--LPHYL-MY-
**Echinoderms**				
Apostichopus japonicus-4K	PIK54083.1	FV----TRE-VEMMHNILP--HKYMVEMH	G	K--LP-YM-MY-
**Hemichordates**				
Saccoglossus kowalevskii-4K	XP_002732674.1	-V----SRE-VEMMHNI-KQ-HQFTVEHH	G	K--LPHYL-MY-
**PROTEOSOME**				
**Arthropods**				
Apis mellifera-4K	XP_392797.3	F----LTGE-VERMNSFLKH-HPYIVERH	G	K--LP-YL-MY-
Drosophila melanogaster-4K	NP_001033805.1	F---SLTSE-IERMHAFLKQ-HPY-VERH	G	K--LP-YL-MY-
**Nematoda**				
Trichinella papuae-4K	KRZ73132.1	FL---LCAE-VAEVHSIL-K-H-YIVEHH	S	K--LP--L-MY-
Trichinella spiralis-4K	XP_003378394.1	FL---LCAE-VAEVHSIL-K-H-YIVEHH	S	K--LP--L-MY-
Trichinella britovi-4K	KRY60612.1	FL---LCAE-VAEVHSIL-K-H-YIVEHH	S	K--LP--L-MY-
Toxocara canis-4K	KHN80062.1	FV---MDSEAVAEIHSILRF-HEY-VEKH	G	K--LP-YL--Y-
Brugia malayi-4K	XP_001902899.1	FV--SMDSEAVAE-HSVLRS-HEY-VEKH	G	K--LP--L--Y-
Loa loa-4K	XP_020305842.1	FV--SMDSEAVAE-HSVLRS-HEY-VEKH	G	K--LP--L--Y-
Caenorhabditis brenneri-4K	EGT42751.1	FV--SMDSEAVAE-HSVLRN-HQY-VEKQ	G	K--LP-YL--Y-
Caenorhabditis elegans-4K	NP_497500.1	FV--SMDSEAVAE-HSVLRN-HQY-VEKQ	G	K--LP-YL--Y-
Strongyloides ratti-4K	CEF67371.1	FV---LDSDNVGE-LSILPK-HEY-VSKR	G	K--LP-NL--Y-
**Mollusca**				
Crassostrea virginica-4K	XP_022305822.1	-F---LVSE-VEQ-HLILKQ-HQFIVE-H	A	K--LP-YL-MY-
Mizuhopecten yessoensis-4K	XP_021377781.1	FFV--LVSE-VEQMHHLLKQ-HQYIVETH	A	Q--LP-YL-MY-
Octopus bimaculoides-alpha-4K	XP_014773204.1	-F---LVSE-VEQMHHILKQ-HSYIVEIH	A	Q--LP-YL-MY-
Biomphalaria glabrata-beta-4K	XP_013081123.1	FF---LVSE-VEMMHHLLKQ-HQYIVECH	A	Q--LP-YLAMY-
**Annelida**				
Helobdella robusta-4K	XP_009023323.1	FF----SSEQVAEMHRILK--HQYIVERH	A	D--LP-YL-MY-
Capitella teleta-4K	ELU08768.1	FVV---LSE-VAEMHRI-KD-HQ-IVERH	S	E--LP-YM-MY-

**Platyhelminthes**				
Macrostomum lignano-4K	PAA91900.1	-V----GSE-VEQMHHIL-A-HGYIVECS	A	S--LP-YL-MY-
Clonorchis sinensis-4K	GAA55841.1	FVA-A-SSE-VEQMHNLLED--AYIVHCH	G	Q--LP-YL--Y-
**Tardigrada**				
Hypsibius dujardini-4K	OQV16814.1	F----LTRE-VEQMHHILKH-HEY-VEHH	C	K--LP-YF-AY-
Ramazzottius varieornatus-4K	GAV02130.1	F----LTRE-VEQMHHILKH-HEY-VEHH	C	K--LP-YF-IY-
**PLACOZOA**				
Trichoplax adhaerens-4K	XP_002111279.1	-VV---DSYDVETMHLIL-G-HQYIIE-K	G	V--LP-YL-MY-
**PORIFERA**				
Amphimedon queenslandica-4K	XP_019863884.1	L---SLSSE-VAL-HQIL-P-HA-IVTQE	G	Q--LP-YL-MY-
**CNIDARIA**				
Hydra vulgaris-4K	XP_002161268.1	FY----ERE-VEMMHQIMPS-HQY-VESH	S	---LP-YL-MY-
Exaiptasia pallida-4K	XP_020903371.1	FY----ESE-VER-HTFL---HHY-VKQR	A	---LP-YLAMY-
Nematostella vectensis-4K	XP_001647531.1	FYV-V-ESE-VER-HNIL-P-HH-IVEQH	A	---LP-YLAMY-
**CHAENOFLAGELLATA**				
Monosiga brevicollis-4K	EDQ89588.1	LVL-SLSKE-VLQFHHTF-A-HS-IVELG	C	R--LPRYL-MY-
Salpingoeca rosetta-4K	XP_004998565.1	LV---LAKE-VASFHHTFKQ--SYIVECD	G	D--LARYL-MY-
**FILASTEREA**				
Capsaspora owczarzaki-4K	XP_004364933.1	F-V-SMSKI-VDLMHNILPL-HTYIVETS	A	R--LP-YV-MY-
**AMOEBOZOA**				
Cavenderia fasciculata	XP_004359026.1	F-----PKD-AKL--SLLPA-TE-LTQ--		---LPR-F--F-
Tieghemostelium lacteum	KYQ96762.1	F-L---PKD-AKI--SLLPS-LD-IQS--		-S-LPR-F--Y
Acanthamoeba castellanii	XP_004336615.1	FML---TRG-AKF-KAILPK----IIQ--		S---AR-F-F--
**APICOMPLEXA**				
Leishmania infantum	XP_001468654.1	WV---MTEQ-SDF---ILHR--Y------		F--LPH-V-H--
Toxoplasma gondii	EPT29018.1	FM----SK-TAMF--SILLD--E--MA--		DS-LTR-F---A
Plasmodium vivax	KMZ89198.1	-----VCKNI-NLSKALLPK--S-I-S--		DS-LTRL--I-C
**OTHERS EUKARYOTES**				
Naegleria gruberi	XP_002670454.1	-ML--VTKK-SKF---ILPD-----MA--		----TR---MYS
Fonticula alba	XP_009497027.1	------SKT--LFF--ILHA-HSF--E--		D--LSR-C--Y-
Ectocarpus siliculosus	CBJ28352.1	----NMKRA-AKFF-SILPQ--E-H-TH-		DSVLIR-C-MYL
Ostreococcus tauri	XP_003080505.1	F----MRK--MLN-KSWLHL--K--HEY-		ES-LPK-F-IYS
Saprolegnia parasitica	XP_012202766.1	FM---QTQG-SKF--RILPH--KF-ME--		---VTR---M--
Phytophthora infestans	XP_002897706.1	FM---QTKD-SKF--RILPH--KF-ME--		----TR---M--
Dictyostelium discoideum	XP_629858.1	-VL---PKR-AKL--SLLP---E-MKR--		-S-LTK-F----
Thecamonas trahens	XP_013760701.1	FV----T-A-AKF--SILYR--HYMYS--		---LSK-C----
Emiliania huxleyi CCMP1516	XP_005786295.1	-LA-SMTRA--HA-LQLLPQ-CR-MQSQ-		RS-LSRLS-CYI
Trichomonas vaginalis G3	XP_001579530.1	-V---QTKD-MKV-Q-ILPQ-FD--TNH-		D--VNH---AY-

Figure S3. Detailed species distribution information for the 2 aa deletion in PIP4K in Figure 3.

**Vertebrates PIP5K (>100)**		100 133	
Homo sapiens-Alpha-5K	NP_001129108.1	DFYVVESIFFPSEGSNLT PA HHYNDFRFKTYAPV	
Serinus canaria-Alpha-5K	XP_018781091.1	------------------ -- --------------	
Python bivittatus-Alpha-5K	XP_015744820.1	---M-------G------ -- --------------	
Protobothrops mucrosquamatus-Alpha-5K	XP_015680374.1	-----------G------ -- --------------	
Xenopus tropicalis-Alpha-5K	NP_001006899.1	-----------G------ -- --------------	
Pundamilia nyererei-Alpha-5K	XP_005740686.1	--E--------C------ -- ---G---------M	
Maylandia zebra-Alpha-5K	XP_004541373.2	--V--------------- -- ---S---------I	
Homo sapiens-Beta-5K	NP_003549.1	-------V-L-------- -- ---P---------L	
Serinus canaria-Beta-5K	XP_009092185.1	-------V-L-------- -- ---P---------L	
Protobothrops mucrosquamatus-Beta-5K	XP_015679138.1	-------V-L-------- -- ---P---------L	
Xenopus tropicalis-Beta-5K	XP_004910836.1	-------V-L-------- -- ---P---------L	
Maylandia zebra-Beta-5K	XP_004538536.1	--S----V-L-------- -- --FP---L-----L	
Pundamilia nyererei-Beta-5K	XP_005725614.1	--S----V-L-------- -- --FP---L-----L	
Homo sapiens-Gamma-5K	XP_011526147.1	------------------ -- --FQ----------	
Serinus canaria-Gamma-5K	XP_018777319.1	------------------ -- ---A----------	
Sturnus vulgaris-Gamma-5K	XP_014739865.1	------------------ -- ---A----------	
Protobothrops mucrosquamatus-Gamma-5K	XP_015666168.1	------------------ -- ---P----------	
Xenopus tropicalis-Gamma-5K	XP_017946547.1	------------------ -- ---P----------	
Maylandia zebra-Gamma-5K	XP_012772288.1	------------------ -- --FP----------	
Pundamilia nyererei-Gamma-5K	XP_005721178.1	------------------ -- --FP----------	
**Tunicata**			
Ciona intestinalis-5K	XP_018673474.1	--Q---T-V--------- -- --FGE-T-R----T	
Oikopleura dioica-5K	CBY41353.1	--E-I-T-V--KA-TT-- -H -TFD-----S---L	
**Cephalochordata/ Amphioxus**			
Branchiostoma floridae-5K	XP_002591361.1	--S----V---G------ -- -RCP---------M	
**Echinodermata**			
Apostichopus japonicus-5K	PIK60516.1	--A----V---R----I- Q- -Q-P----------	
**Hemichordata**			
Saccoglossus kowalevskii-5K	XP_006821157.1	--A----V---------- A- -K-P----------	
**PROTEOSOME**			
**Arthropods**			
Apis mellifera-5K	XP_006571145.1	--MT--TTN-------H- -- --FSE-K--N---I	
Drosophila melanogaster-5K	NP_611729.2	--WEI---T--P---S-- -- ---SE--Y-I---I	
**Nematoda**			
Trichinella britovi-5K	KRY55394.1	--EY--QVY---A-THV- -S -P-S---------I	
Trichinella spiralis-5K	KRY35256.1	--EY--QVY---A-THV- -S -P-S---------I	
Trichinella papuae-5K	KRZ69085.1	--EY--QVY--*A-THV- -S -P-S---------I	*RQSYFLNLFIPIWKFACYFS
Caenorhabditis elegans-5K	NP_491576.2	--EK-DIVA--AA--TI- -S -SFG----R----I	
Wuchereria bancrofti-5K	EJW88844.1	--D--HTVS---S--QS- -S -S-G----Q----I	
Loa loa-5K	XP_020303847.1	--D--HTVS---S--QS- -S -N-G----Q----I	
Brugia malayi-5K	CDP97235.1	--D--HTVS---N--QS- -S -S-G----Q----I	
Caenorhabditis brenneri-5K	EGT37979.1	--EK-DIVA--*A--SI- -S -SFG----R----I	*AIILCFYS
Strongyloides ratti-5K	CEF65455.1	--QEIL-VA------IQ- -S -Q-----------I	
**Mollusca**			
Biomphalaria glabrata-beta-5K	XP_013074799.1	--G---VV---G------ -- ---S----------	
Octopus bimaculoides-alpha-5K	XP_014789196.1	--A----V---G------ -- ---S----------	
Crassostrea virginica-5K	XP_022306935.1	--A-I--V-Y-A------ -- --CS---------I	
Mizuhopecten yessoensis-5K	XP_021350909.1	--Q--D-V---A------ -- --CS---------I	
**Annelida**			
Helobdella robusta-5K	XP_009026309.1	--D--------R----S- -- -R-S----------	
Capitella teleta-5K	ELT87243.1	--TI-D-V-H-R------ -- ---S--L------F	
**Platyhelminthes**			
Macrostomum lignano-5K	PAA67906.1	--G-L-IVD--RD-GKF- V- -SITS-T------M	
Clonorchis sinensis-5K	GAA56497.1	--QTIDTVQ--AC-TKT- -- -SLS---------I	
**Tardigrada**			
Hypsibius dujardini-5K	OQV12309.1	--AI--KV-HKK--TVS- -- -GL---T--S---I	
Ramazzottius varieornatus-5K	GAU91082.1	--ENT-IV-H-EA-TKF- -- -NFG--H------I	

**PLACOZOA**				
(the phylum Placozoa comprises only one species)				
Trichoplax adhaerens-5K	XP_002108154.1	--QDIDVV---RD--TS-	--	--HA--T------I
**PORIFERA**				
Amphimedon queenslandica-5K	XP_019849193.1	--N-I-TVD---G--AI-	Q-	-K-K--T-T--S--
**CNIDARIA**				
Hydra vulgaris-5K	XP_012564577.1	---F--KVW---S--KE-	-S	-KFY---------
Exaiptasia pallida-5K	XP_020899131.1	--AEI-TVW-----TKG-	-S	-KFS--K--S----
Nematostella vectensis-5K	XP_001633067.1	--AQI-TVV-----TRE-	-S	-KFS--K--S----
**CHAENOFLAGELLATA**				
Salpingoeca rosetta-5K	XP_004997164.1	--QE---LYY-R--T-S-	-P	-KFEN-K-Y----R
Monosiga brevicollis-5K	EDQ89244.1	--DE---QNY-R--TST-	--	--FES-K-S-----
**FILASTEREA** (only two species known)				
(share fig 2 and fig 3 CSIs)				
Capsaspora owczarzaki-5K	XP_004348939.1	--EEH-IVD-----GTY-	--	-SS-S----S---R
**Vertebrates PIP4K (>100)**				
Homo sapiens-Alpha-4K	XP_011523628.1	--KAYSK-KVDNHLF-KE		NLPSR-K--E-C-M
Serinus canaria-Alpha-4K	XP_009084143.1	--KAYSK-KVDNHLF-KE		NMPSH-K--E-C-M
Python bivittatus-Alpha-4K	XP_007436413.1	--KAYSK-KVDNHLF-KE		NMPSH-K--E-C-M
Protobothrops mucrosquamatus-Alpha-4K	XP_015667052.1	--KAYSK-KVDNHLF-KE		NMPSH-K--E-C--
Xenopus tropicalis-Alpha-4K	NP_001123723.1	--KAYSK-KVDNHLF-KE		NMPSH-K--E-C-M
Maylandia zebra-Alpha-4K	XP_004546610.1	--KAYSK-KVDNHLF-KE		NMPSH-K--E-C-L
Homo sapiens-Beta-4K	EAW60533.1	--KAYSK-KVDNHLF-KE		NLPSR-K--E-C-M
Serinus canaria-Beta-4K	XP_009094714.2	--KAYSK-KVDNHLF-KE		NLPSR-K--E-C-L
Python bivittatus-Beta-4K	XP_007429905.1	--KAYSK-KVDNHLF-KE		NLPSR-K--E-C-L
Protobothrops mucrosquamatus-Beta-4K	XP_015684863.1	--KAYRK-KVDNHLF-KE		NLPSH-K--D-C-L
Xenopus tropicalis-Beta-4K	XP_002940195.1	--KAYSK-KVDNHLF-KE		NLPSR-K--E-C-M
Maylandia zebra-Beta-4K	XP_004538792.1	--KAYSK-KVDNHLF-KE		NLPSR-K--E-C-M
Homo sapiens-Gamma-4K	XP_011537049.1	--KASSK-KVNNHLFHRE		NLPSH-K--E-C-Q
Sturnus vulgaris-Gamma-4K	XP_014747327.1	--KASSK-KVNNHLF-RE		NLPSH-K--E-C-Q
Python bivittatus-Gamma-4K	XP_007422116.1	--KASSK-KVNNHLF-RE		NLPGH-K--E-C-Q
Protobothrops mucrosquamatus-Gamma-4K	XP_015679464.1	--KASSKVKVNNHLF-RE		NLPGH-K--E-C-Q
Xenopus tropicali-Gamma-4K	XP_017946647.1	--KANSK-KVTNHLF-RE		NLPSH-K--D-C-Q
Maylandia zebra-Gamma-4K	XP_004560306.1	--KANTK-KVNNHLF-KE		NLPGH-K--E-C-Q
Pundamilia nyererei-Gamma-4K	XP_005741105.1	--KANTK-KVNNHLF-KE		NLPGH-K--E-C-Q
**Tunicata**				
Ciona intestinalis-4K	XP_002119441.3	--KAYSKVKVDNHIF-KE		NLPSH-KL-E-C-L
Oikopleura dioica-4K	CBY18232.1	--KAYSKLKVENHAF-R-		LLPGHYKV-E-C-L
**Cephalochordata/ Amphioxus**				
Branchiostoma floridae -4K	XP_002599487.1	--KAYSKVKVDNQYF-KE		NLPSH-KV-E-C-L
**Echinodermata**				
Apostichopus japonicus-4K	PIK54083.1	--KAYSK-KVDNHLY-RE		NLPSH-KV-E-C-M
**Hemichordata**				
Saccoglossus kowalevskii-4K	XP_002732674.1	--KAYTKTKVDNHCF-KE		NLPSH-KV-E-C-L
**PROTEOSOME**				
**Arthropods**				
Apis mellifera-4K	XP_392797.3	--RAYSKLKVDYHFF-KE		NMPSH-T--E-C-L
Drosophila melanogaster-4K	NP_001033805.1	--RAYSK-KVDNHLF-KE		NMPSH-KV-E-C-
**Nematoda**				
Trichinella spiralis-4K	XP_003378394.1	--KAFSK-RIDNHLF-KE		SMPSH-KV-E-C-N
Trichinella britovi-4K	KRY60612.1	--KAFSK-RIDNHLF-KE		SMPSH-KV-E-C-N
Trichinella papuae-4K	KRZ73132.1	--KAFSK-RIDNHLF-KE		SMPSH-KV-E-C-N
Caenorhabditis elegans-4K	NP_497500.1	--KAYSKVKIDNHNF-KD		IMPSHYKV-E-C-N
Caenorhabditis brenneri-4K	EGT42751.1	--KAYSKVKIDNHNF-KD		IMPSHYKV-E-C-N
Strongyloides ratti-4K	CEF67371.1	--KASSK-KVDNHFF-KE		-MPSH-KI-Q-C-N
Wuchereria bancrofti-4K	EJW74307.1	--KASIK-KIDNHFF-KD		NMPSH-KI-D-C-N
Loa loa-4K	XP_020305842.1	--KASIK-KIDNHFF-KD		NMPSH-KI-D-C-N
Brugia malayi-4K	XP_001902899.1	--KASIK-KIDNHFF-KD		NMPSH-KI-D-C-N
Toxocara canis-4K	KHN80062.1	--RASTK-KVDYHLF-KD		NMPSH-KV-D-C-N
**Mollusca**				
Crassostrea virginica-4K	XP_022305822.1	--KAFSKVHVDYHKFQKE		SMPSH-K--E-C-I
Mizuhopecten yessoensis-4K	XP_021377781.1	--KAFSK-RVDNHSY-KE		SMPSH-KV-E-C--

Octopus bimaculoides-alpha-4K	XP_014773204.1	--KAFTKVRKDNHLY-KE		NMPSH-KV-E-C-M
Biomphalaria glabrata-beta-4K	XP_013081123.1	--KSYSK-RVDNHMY-KD		NMPSR-KV-E-C-I
**Annelida**				
Helobdella robusta-4K	XP_009023323.1	--KAYTKTRVDNHMF-KE		NMPSH-K--E-C-N
Capitella teleta-4K	ELU08768.1	--KSHSKVRVDNHLF-KD		NMPSH-KV-E-C-L
**Platyhelminthes**				
Macrostomum lignano-4K	PAA91900.1	--KSNLKVKVDNHLF-KD		SMPSK-K--E-C-L
Clonorchis sinensis-4K	GAA55841.1	--KAYMKVKINNHLF-KE		NMPSR-K--E-C-I
**Tardigrada**				
Hypsibius dujardini-4K	OQV16814.1	--KAYNK-KIDRQ-F-KD		NMPSH-KV-E-C-L
Ramazzottius varieornatus-4K	GAV02130.1	--KAYNK-KIDRQYF-KD		NMPSH-KV-E-C-L
**PLACOZOA**				
(this phylum Placozoa comprises only one species)				
Trichoplax adhaerens-4K	XP_002111279.1	--KAYSKTKVENYYF-EE		TLPGH-KY-E-C-K
**PORIFERA**				
Amphimedon queenslandica-4K	XP_019863884.11	--KAFSK-RVENQHYSKE		YLPGH-K--E-C-I
**CNIDARIA**				
Exaiptasia pallida-4K	XP_020903371.18	--KAFSK-RVDNHSY-KE		NLPSH-K--E-C-M
Hydra vulgaris-4K	XP_002161268.1	--KAYSK-KIDNHLY-KE		NMPGH-K--E-M-L
Nematostella vectensis-4K	XP_001647531.1	--KAYSKVRVDNHLF-KE		NLPSH-K--E-C-M
**CHAENOFLAGELLATA**				
Monosiga brevicollis-4K	EDQ89588.1	--KAHSK-AVANHLY-DH		ELPSK-KV-E-C-L
Salpingoeca rosetta-4K	XP_004998565.1	--KAFSKVQVHNQYY-EQ		ELP-K-KV-E-C--
**FILASTEREA** (only two species known)				
(share fig 2 and fig 3 CSIs)				
Capsaspora owczarzaki-4K	XP_004364933.1	H-KAYSKTKIHNHQF-TS		DLPMK-K--E-C-I
**ICHTHYOSPOREA**				
(smaller size missing this region)				
Sphaeroforma arctica-5K	XP_014156830.1			
Sphaeroforma arctica-4K	XP_014150314.1			
**Fungi**				
Saccharomyce scerevisiae	EGA59287.1	--RFTKKLA-DYH-NE--	-	SSQYA-K--D-C-E
Aspergillus nidulans	XP_660370.1	--KAKHKFS-DIT-NE--	-	SAQY--K--D---W
Kluyveromyces lactis	XP_451188.1	--KLTKKLA-DYH-NE--	-	SSQYA-K--D-C-E
Candida albicans	KGU13027.1	--KATKKLS-NFD--E--	-	SSKY--K--D-C-E
Clavispora lusitaniae	XP_002615347.1	--SATKKLT-SMD--E--	-	SSKY--K--D-S-E
Lachancea nothofagi CBS 11611	SCV03977.1	--RLTKKLA-DFH-NE--	-	SSQYA-K--D-C-E
Kazachstania naganishii CBS 87	XP_022462372.1	--KCSKKLA-DYH-NE--	-	SSQYA-K--D-C-E
Naumovozyma castellii CBS 4309	XP_003674750.1	--KFNKKLA-DYH-NE--	-	SSEYA-K--D-C-E
Torulaspora delbrueckii	XP_003682564.1	--KFTKKLA-DYH-NE--	-	SSQYA-K--D-S-E
Vanderwaltozyma polyspora DSM	XP_001643104.1	--SFTKKLA-DYH-NE--	-	SSQYA-K--D---E
Tetrapisispora blattae CBS 628	XP_004182131.1	--TYTKKLA-DYH-NE--	-	SSQYA-K--D-C-E
Wickerhamomyces ciferrii	XP_011276550.1	--RMNKKLV-DVS-NE--	-	SSKY--K--D-C-T
Cyberlindnera jadinii	CEP23152.1	--KLIQKLR-D-S-NE--	-	SSKY--K--D-Y-T
Tuber aestivum	CUS11234.1	--DAKHKFS-DIT-NE--	-	SAKY--K--D-S-W
Coniosporium apollinis CBS 100	XP_007784276.1	--KARHKFS-DIT-NE--	-	SAKY--K--D---W
Cyphellophora europaea CBS 101	XP_008713371.1	--DAKHKFS-DVT-NE--	-	SAKY--K--D---W
Pyrenophora teres f. teres 0-1	EFQ90510.1	--DAKHKFS-DIT-NE--	-	SAKY--K--D---W
Fonsecaea multimorphosa CBS 10	XP_016637750.1	--DAKHKFS-DIT-NE--	-	SAKY--K--D-S-W
Endocarpon pusillum Z07020	XP_007803590.1	--DARHKFS-DIT-NE--	-	SAKY--K--D---W
Cladophialophora bantiana CBS	XP_016624332.1	--DAKHKFS-DIT-NE--	-	SAKY--K--D-S-W
Capronia epimyces CBS 606.96	XP_007737551.1	--AARHKFS-DIT-NE--	-	SAKY--K--D-S-W
Exophiala oligosperma	XP_016268534.1	--DAKHKFS-DIT-NE--	-	SAKY--K--D---W
Leptosphaeria maculans JN3	XP_003841630.1	--DAKHKFS-DIT-NE--	-	SAKY--K--D---W
Paraphaeosphaeria sporulosa	XP_018032922.1	--DAKHKFS-DIA-NE--	-	SAKY--K--D---W
Aureobasidium melanogenum CBS	KEQ67430.1	--DAKHKFS-DIT-NE--	-	SAKY--K--D---W
Pyronema omphalodes	CCX06330.1	--DAKHKFS-DVT-NE--	-	SAKY--K--D-S-W
Phialophora attae	XP_018004907.1	--DAKHNFS-DVT-NE--	-	SAKY--K--D-S-W
**Plants**				
Glycine max	XP_006584672.1	-PKEKFWTR--P---KF-	-Q	--SV---W-D-C-M
Coffea canephora	CDP17283.1	-TREKLWTK--P---KY-	-P	-QSC---W-D-C-L
Oryza sativa	XP_015633005.1	-PKEKFWTR--P---KV-	-P	-SSS---W-D-C-M
Arabidopsis thaliana	NP_173617.1	-PKEKFWTR--P--TKT-	-P	-QSV---W-D-C-L

Genlisea aurea	EPS66188.1	-PKEKFWTK--P---KI- -P -QSCE-KW-D-C-K
Morus notabilis	EXB62690.1	-PKEKVWTK--P---KY- -P -SSC---W-D-C--
Macleaya cordata	OVA18495.1	-PKEKVWTK--P---KY- -P -QSC---W-D-C-L
Aquilegia coerulea	PIA60189.1	-PKEKVWTR--P---KH- -P -QSC---W-D-C-L
Trema orientalis	PON98771.1	-PKEKVWTK--P---KY- -P -QSC---W-D-C--
Parasponia andersonii	PON53305.1	-PKEKVWTK--P---KY- -P -QSC---W-D-C--
Malus domestica	XP_008375376.1	-PKEKVWTK--P---KY- -P -QSC---W-D-C--
Ipomoea nil	XP_019151708.1	-PKEKVWTR--P---KH- -P -QSC---W-D-C-L
Apostasia shenzhenica	PKA53367.1	-PKEKVWTR--P---KQ- -P -QSC---W-D-C-L
Elaeis guineensis	XP_019706081.1	-PKEKVWTR--P--TKQ- -P -QSC---W-D-C-L
Ziziphus jujuba	XP_015902998.1	-PKEKVWTR--P--TKH- -P -QSTE-KW-D-C-L
Vitis vinifera	XP_002267007.3	-PKEKVWTR--P---KY- -P -QSSE-KW-D-C-L
Pyrus x bretschneideri	XP_009372761.1	-PKEKVWTK--P---KY- -P -QSC--KW-D-C--
Daucus carota subsp. sativus	XP_017254749.1	-TKEKVWTR--P---KN- -P -QSC--KW-D-C-L
Carica papaya	XP_021908574.1	-PKEKVWTR--P--TKY- -P -QSCE-KW-D-C-L
Panicum hallii	PAN49615.1	-PKEKVWTK--P---KY- -P -NSC--KW-D-C-K
Musa acuminata subsp. malaccen	XP_018675993.1	-PKEKIWTR--P--TKQ- -P -QSC--KW-D-C-L
Prunus persica	XP_020413784.1	-PREKVWTK--P---KY- -P -QSC--KW-D-C--
Sorghum bicolor	OQU92574.1	-PKEKVWTK--P---KY- -P -NSC--KW-D-C-K
Ricinus communis	XP_002526916.1	-PKEKVWTR--P--TKY- -P -QSSE-KW-D-C-L
Camelina sativa	XP_010427550.1	-PKEKVWTR--P--TKY- -P -QSTE-KW-D-C-L
Brassica napus	XP_013721154.2	-PKEKVWTR--P--TKY- -P -QSSE-KW-D-C-L
Juglans regia	XP_018849425.1	-PKEKVWTK--P---KH- -P -QSA--KW-D-C--
Fragaria vesca subsp. vesca	XP_004307106.1	-PREKVWTK--P---KY- -P -QSS--KW-D-C--
Eutrema salsugineum	ESQ44381.1	-PKEKVWTR--P--TKY- -P -QSSE-KW-D-C-L
Eucalyptus grandis	KCW90045.1	-PKQKVWTR--P---KY- -P -QACE-KW-D-C-L
Capsella rubella	XP_023638231.1	-PKEKVWTR--P--TKY- -P -QSSE-KW-D-C-L
Manihot esculenta	XP_021624297.1	-PKEKVWTR--P--TKC- -P -QSSE-KW-D-C-L
Dorcoceras hygrometricum	KZV47292.1	-PKDKIWTR--P---KH- -P -QSCE-KW-D-C-L
Cephalotus follicularis	GAV61641.1	-PKEKVWTR--PG-TKY- -P -QSSE-KW-D-C-L
Raphanus sativus	XP_018489538.1	-PKEKVWTR--P--TKH- -P -QSSE-KW-D-C-L
Nelumbo nucifera	XP_010276643.1	-PKEKVWTK--P---KH- -P -QSSE-KW-D-C--
Cajanus cajan	XP_020240276.1	-PKEKVWTK--P---KH- -P -PSCE--W-D-C--
Capsicum annuum	XP_016557282.1	-TKEKIWTK--T---KY- -P -QSCE-KW-D-C-L
Sesamum indicum	XP_011088692.1	-PRDKTWTR--PQ--KH- -P -QSCE--W-D-C-L
Hevea brasiliensis	XP_021677612.1	-PKEKVWTR-----TKH- -P -QSSE-KW-D-C-L
Trifolium pratense	PNY15056.1	-PKEKVWTR--A---KH- -P -PSCE--W-D-C-A
Solanum lycopersicum	XP_004230026.1	-TKEKVWTK--T---KH- -P -QSCE-KW-D-C-L
Erythranthe guttata	XP_012830685.1	-PRDKVWTK--PL--KH- -P -QSCE-KW-D-C-L
**AMOEBOZOA**		
Cavenderia fasciculata	XP_004359026.1	FYLSPKELR-D-T-TSQ- E- -STGP-K--D-C-M
Polysphondylium pallidum	XP_020433305.1	FYQSPKELR-DTA--Q-- E- -STGP-K--D-C--
Heterostelium album PN500	XP_020433305.1	FYQSPKELR-DTA--Q-- E- -STGP-K--D-C--
Dictyostelium discoideum AX4	XP_647148.1	FYVSPKTQR-E-A-TAM- N- -STGA-K--D-C-N
Tieghemostelium lacteum	KYQ96762.1	FYNSPKTLR-DAV--P-- N- -STGA-K--D-C-H
**APICOMPLEXA**		
Leishmania infantum	XP_001468654.1	RTQTLADFSSDD-DDEDE EN MPPVS-T-TDFS-M
Toxoplasma gondii	EPT29018.1	MKEKFSILPNTGMVKSIS DR RSLFAV--VD---M
Plasmodium vivax	KMZ89198.1	NTSNIINL-KHKDLLE-P TS NAQHRVV--N----
**OTHERS EUKARYOTES**		
Fonticula alba	XP_009497027.1	--TE-QKMV-DIT-NE-L -Y SK Y--K--D-M-W
Ostreococcus tauri	XP_003080505.1	--ERTVRQI--RS--SA- -P -FART-KW-E-R-E
Saprolegnia parasitica	XP_012202766.1	RRLAHRVVWA-PTAYVVS VS GVHY-YVLRV-E-I
Thecamonas trahens	XP_013753394.1	--FFTLKF-IL-TVWYA- -S -TRSSLLG-SFHFI
Naegleria gruberi	XP_002670454.1	--TNINKMP--EK--TR- -P -EFPF-D-YD-S-F

Figure S4. Detailed species distribution information for the 1 aa CSI shown in Figure 4.

Species	Accession	141		183
**Vertebrates PIP5K (>100)**				
Homo sapiens-Alpha-5K	NP_001129108.1	LFGIRPDDYLYSLCSE		PLIELCSSGASGSLFYVSSDDEFIIKT
Serinus canaria-Alpha-5K	XP_018781091.1	----------------		-----SN--------------------
Protobothrops mucrosquamatus-Alpha-5K	XP_015680374.1	----------C-----		-----SN------I-------------
Xenopus tropicalis-Alpha-5K	NP_001006899.1	--S-----------N-		-----SNP-----V----G--------
Maylandia zebra-Alpha-5K	XP_004541373.2	--------------N-		-----SNP-------------------
Homo sapiens-Beta-5K	NP_003549.1	----K-------I---		-----SNP-------F-T---------
Serinus canaria-Beta-5K	XP_009092185.1	----K-------I---		-----SNP-------F-T-G-------
Protobothrops mucrosquamatus-Beta-5K	XP_015679138.1	----K-------I---		-----SNP-------F-T---------
Xenopus tropicalis-Beta-5K	XP_004910836.1	----K-----------		-----SNP-----I-F-T---------
Maylandia zebra-Beta-5K	XP_004538536.1	----K-------I-N-		-----SNP---S-W--LT---------
Homo sapiens-Gamma-5K	XP_011526147.1	--------------N-		-----SNP---------T---------
Serinus canaria-Gamma-5K	XP_018777319.1	--------------N-		-----SNP---------T---------
Protobothrops mucrosquamatus-Gamma-5K	XP_015666168.1	--------------N-		-----SNP---------T---------
Xenopus tropicalis-Gamma-5K	XP_017946547.1	--------------N-		-----SNP---------T---------
Maylandia zebra-Gamma-5K	XP_012772288.1	--------------N-		-----SNP-----I---TR--------
**Vertebrates PIP4K (>100)**				
Homo sapiens-Alpha-4K	XP_011523628.1	R---DDQ--QN-VTRS		AP-NSD-Q-RC-TR-LTTY-RR-V---
Serinus canaria-Alpha-4K	XP_009084143.1	R---DDQ-FQN--TRS		APLAND-QAR--AR-HT-Y-KRY----
Python bivittatus-Alpha-4K	XP_007436413.1	R---DDQ-FQN--TRS		CPLAND-PAR--AR-HS-Y-KRYV---
Xenopus tropicalis-Alpha-4K	NP_001123723.1	R---DDQ-F-N--TRY		SPLAND-QAR--AR-HT-C-KRY----
Maylandia zebra-Alpha-4K	XP_004546610.1	R---DDQ-F-N--TRS		APLNSEAQ-R--AR-HT-Y-KRYV---
Homo sapiens-Beta-4K	EAW60533.1	R---DDQ--QN-VTRS		AP-NSD-Q-RC-TR-LTTY-RR-V---
Serinus canaria-Beta-4K	XP_009094714.2	R---DDQ--QN-VTRS		APVNSD-Q-RC-AR-LTTY-RR-V--A
Python bivittatus-Beta-4K	XP_007429905.1	R---DDQ--QN-VTRS		APVYSD-H-RC-VR-LTTY-RR-V--A
Xenopus tropicalis-Beta-4K	XP_002940195.1	R---DDQ--QN--TRS		APVNSENQ-RF--R-LTTY-RR-V---
Maylandia zebra-Beta-4K	XP_004538792.1	R-C-DDQ--QN--TRS		APLNSDTQ-RF-NRILS-Y-HR-V---
Homo sapiens-Gamma-4K	XP_011537049.1	R---DDQ---V--TRN		-PS-SEG -DGR-LI-Y-RTLV--E
Sturnus vulgaris-Gamma-4K	XP_014747327.1	R--VDDQ--QV--TRS		-PRWAG- -HRLLL-A-RTLVL-E
Python bivittatus-Gamma-4K	XP_007422116.1	R-N-DDQ--QV--TRS		-PTYETE G-GR-LL-Y-RTVV--E
Xenopus tropicalis	XP_017946647.1	R---DDQ-FQA--TRS		SPYCESE GHDGR-LL-Y-KTLV--E
Maylandia zebra-Gamma-4K	XP_004560306.1	R---EDL--QV--TRS		-PFSVDD QGEG-LLN-Y-RTLVV-Q
**Tunicata**				
Ciona intestinalis-5K	XP_018673474.1	---------ML-ISRL		--R--SNP-------F-TH--------
Ciona intestinalis-4K	XP_002119441.3	R---ADK--VS---VN		QPFRVDDK-R---R-LH-F-HKYV---
Oikopleura dioica-5K	CBY09966.1	I-N-STE-F-H-IGAR		S--PIGNP-----C-WITH-----V--
Oikopleura dioica-4K	CBY32977.1	I-D-SVEG-IH-IIH-		--TAIGNP-------WITH--------
**Cephalochordata/ Amphioxus**				
Branchiostoma floridae-5K	XP_002591361.1	----Q---F-I-----		--R--SNP--------LTA-----V--
Branchiostoma floridae -4K	XP_002599487.1	R-N-DDV--MN--TRS		QPVNTD-P-R--AR-LM-Y-KRYV---
**Echinodermata**				
Apostichopus japonicus-5K	PIK60516.1	----Q-----I--VKD		--R--SNP-----I--L-N--------
Apostichopus japonicus-4K	PIK54083.1	R-TVAETE-RN-FTFG		-PEYDN---K--AK-MKTH-RR-V---
**Hemichordata**				
Saccoglossus kowalevskii-5K	XP_006821157.1	----Q---F-L---ND		L-R--SNP-----V--LT---------
Saccoglossus kowalevskii-4K	XP_002732674.1	K---NDTE-MN-MT-A		EPVYND-P-R--AR-LI-A-KKYV---
**PROTEOSOME**				
**Arthropoda**				
Apis mellifera-5K	XP_006571145.1	----Q---F-M-M--A		--R--SNP-----I--LTD--------
Drosophila melanogaster-5K	NP_611729.2	----Q---FMM-M-TS		--R--SNP-----I--LTT--------
Apis mellifera-4K	XP_392797.3	R---DDL--KE-MTRS		QP-LED---K--AK--Q-Y-KL-----
Drosophila melanogaster-4K	NP_001033805.1	R--VDDV--RE--TRS		QP-QID---K--AQ--Q-Y-KF----S
**Nematoda**				
Caenorhabditis elegans-5K	NP_491576.2	--H-K-A-F-R-I-T-		--K--SNA-----I----Q--Q-----
Caenorhabditis brenneri-5K	EGT37979.1	--S-K-A-F-R-I-T-		--K--SNA-----I----Q--Q-----
Wuchereria bancrofti-5K	EJW88844.1	--A-KTA-F-R-V-MF		--K--SNA-----I----H--Q-----
Trichinella britovi	KRY55394.1	--N-K-E-FMA---GV		--R--SNP-----I--I----K--V--
Trichinella spiralis	KRY35256.1	--N-K-E-FMA---GV		--R--SNP-----I--I----K--V--
Brugia malayi-5K	CDP97235.1	--A-KTA-F-R-V-MF		--K--SNA-----I----H--Q-----
Loa loa-5K	XP_020303847.1	--A-KTA-F-R-I-MF		--K--SNA-----I----H--Q-----
Strongyloides ratti-5K	CEF65455.1	M-N-K-A-F-R---TQ		--R--SNP-----V-------K-----
Toxocara canis-5K	KHN83910.1	GHRRIDRQGER-I-T-		--K--SNA-----I-------Q-----
Trichinella papuae-4K	KRZ69085.1	--N-K-E-FMA---GV		--R--SNP-----I--I----K--V--
Caenorhabditis elegans-4K	NP_497500.1	Q--VDNFE--R--T-Y	E	-*LLDG-AKD-TPR-FI-Y-KK-V--S *-EPD
Caenorhabditis brenneri-4K	EGT42751.1	Q--VDSFE--R--T-Y	E	-*LLDG-AKD-TPR-F--Y-KK-V--S *-EPD
Wuchereria bancrofti-4K	EJW74307.1	H--VDQNE--R--TYS	E	-EP-QDQVDK---RLF--H-KK-V--S
Ascaris suum-4K	ERG81423.1	Q--VDQNE--RA-TCY	E	-DP-PDQADK--PRLF--Y-KK-V---

Trichinella britovi-4K	KRY60612.1	K-A-DD----K--TKH	E	-VVMEP-GRGG-ARVF--Y-KR-L---
Trichinella spiralis-4K	XP_003378394.1	K-A-DD----K--TKH	E	-VVMEP-GRGG-ARVF--Y-KR-L---
Brugia malayi-4K	XP_001902899.1	H--VDQNE--R--TYS	E	-EP--DQVDK---RLF--H-KK-V--S
Loa loa-4K	XP_020305842.1	H--VDQNE--R--TYS	E	-EP--DQLDK---RLF--H-KK-V--S
Strongyloides ratti-4K	CEF67371.1	Q---SQREF-A--VYN	E	-E LVVAESSGT-KL-I-Y-KK-V---
Toxocara canis-4K	KHN80062.1	H--VDQNE--R--TCY	E	-DP-PDQADK--PRLFI-Y-KK-V---
Trichinella papuae-4K	KRZ73132.1	K-A-DD----K--TKN	E	-VVMEP-GRSG-ARVF--Y-KR-L---
**Mollusca**				
Biomphalaria glabrata-beta-5K	XP_013074799.1	----Q--FML---D-		--K--SNP-----I--LTQ--------
Octopus bimaculoides-alpha-5K	XP_014789196.1	----HA--FML---T-		--Q---NP-----I--ITD--------
Crassostrea virginica-5K	XP_022306935.1	--S-QT--F-L---D-		--K--SNP-----I--L-Q--------
Mizuhopecten yessoensis-5K	XP_021350909.1	----Q---F-L---DD		--K--SNP-----I--LT---------
Crassostrea virginica-4K	XP_022305822.1	R---DDQ--MN-- TK		-PEDID-P-R--ARMMM-H-KKYF---
Mizuhopecten yessoensis-4K	XP_021377781.1	R---DDQ--MN-- TK		QAVDVD-P-R--ARMLM-Q-KK-FV--
Octopus bimaculoides-alpha-4K	XP_014773204.1	R-SVDDI--MN-- TK		QPV-MD-P-R--ARMLL-Q-KKYF---
Biomphalaria glabrata-beta-4K	XP_013081123.1	R--LDDN--MN--SKQ		QP-SCD-P-R--ARMLM-R-KR-F---
**Annelida**				
Helobdella robusta-5K	XP_009026309.1	----Q-E-F-L---N-		--K--SNP-----I--L-N--------
Capitella teleta-5K	ELT87243.1	----Q---F-L---N-		--Q--SNP-----I--ITN--------
Helobdella robusta-4K	XP_009023323.1	R-SVDE-C-MN--VKH		CPTDID-P-K--AR-LI-H-RKY-L--
Capitella teleta-4K	ELU08768.1	R-D-DE-T-GN--TKF		CPYDCD---R--AR-LH-W-KK-VV--
**Platyhelminthes**				
Macrostomum lignano-5K	PAA67906.1	-YN-DISQF-A-I-G-		E-E--SNP-----I--RTA--N-----
Clonorchis sinensis-5K	GAA56497.1	RYKLDIR-F-N-I--R		E-R--SNP-----I--ITQ--------
Macrostomum lignano-4K	PAA91900.1	K---ERTE--D-FIKR		QPQYDA-Q-R---K-LCTYNRHYV---
Clonorchis sinensis-4K	GAA55841.1	R--VNKL--WDAFTGY		QPLWDSAR-K---K-L-TYNRQ-VA-A
**Tardigrada**				
Hypsibius dujardini-5K	OQV12309.1	----Q----ML---DR		AM--ISNP-----I--LTE-----L--
Ramazzottius varieornatus-5K	GAU91082.1	V-D-S-E--MM-I-V-		G---ISNP--------LTE-EM-----
Hypsibius dujardini-4K	OQV16814.1	R-KVTDSQ-ML--T-S		EP-IKDTH-GQ--TY-LTA-KR-----
Ramazzottius varieornatus-4K	GAV02130.1	R-KVTDSQ-ML--TAS		EP-MKDVHDRQ--NY-LTA-KR-----
**PLACOZOA**				
Trichoplax adhaerens-5K	XP_002108154.1	----K-Q-FMI-M-DK		R-K-IRNP-------FLTN--R-----
Trichoplax adhaerens-4K	XP_002111279.1	C-D-DDEQFKQ-IAFS		--M QY-D-- K-F--R-KQYVV--
**PORIFERA**				
Amphimedon queenslandica-5K	XP_019849193.1	A-Q-KAE---LA--HQ		S-R--SNP--------L-A--------
Amphimedon queenslandica-4K	XP_019863884.1	R-K-DDY--MS--TQH		AHLAMDNP-R---T-F-T--KKL---S
**CNIDARIA**				
Hydra vulgaris-5K	XP_012564577.1	----Q-S-F-L--AN-		-IK-ISNP-------F--N--M--V--
Nematostella vectensis-5K	XP_001633067.1	--SM----FMMA--N-		--V--SNP---------TC--------
Exaiptasia pallida-5K	XP_020899131.1	--SMKA--FMM---ND		--Q--SNP---------TC-NQ-----
Hydra vulgaris-4K	XP_002161268.1	R-N-EEQL-AR-FLIQ		PCDSNA--N--AK-LITKNKM-Y---
Exaiptasia pallida-4K	XP_020903371.1	R--VNDQELAK-FLES		PLASS-Q-R--AK-FT-RNKK-Y---
Nematostella vectensis-4K	XP_001647531.1	R---EDK-LAEAFQVP		P-SSD-P-R--AK-FL-QNKR-YV-V
**CHAENOFLAGELLATA**				
Salpingoeca rosetta-5K	XP_004997164.1	I-N-DTA-F-L-M-HK		--R--SNP-------WL-H--R--V--
Salpingoeca rosetta-4K	XP_004998565.1	I-N-DTA-F-L-M-HK		--R--SNP-------WL-H--R--V--
Monosiga brevicollis-5K	EDQ89244.1	K-S-DTG-F-M-M-DS		--R--SNA-------WL-H--L--V--
Monosiga brevicollis-4K	EDQ89588.1	K-S-DTG-F-M-M-DS		--R--SNA-------WL-H--L--V--
**FILASTEREA** (only two species known) (share fig 2 and fig 3 CSIs)				
Capsaspora owczarzaki-5K	XP_004348939.1	A---KAE-FML---N-		--R--SNP--------M-HN-H-----
Capsaspora owczarzaki-4K	XP_004364933.1	R--VDA-Q-----AGA		EP-PVEAN-K--AS--MTH-KR--V-S
**ICHTHYOSPOREA** (partial sequence missing this region)				
Sphaeroforma arctica-5K	XP_014156830.1			
Sphaeroforma arctica-4K	XP_014150314.1			
**Fungi**				
Saccharomyces cerevisiae	NP_010494.1	---LD-A---V--T-K	Y	I-S--N-P-K---F--Y-R-YKY----
Zygosaccharomyces rouxii	XP_002495864.1	---LD-A---V--T-K	Y	I-S--N-P-K---F--F-R-YKY----
Kluyveromyces lactis	XP_451188.1	---LD-A---M--T-K	Y	I-S--N-P-K---F--F-R-YKY----
Schizosaccharomyces pombe	BAA87265.1	--HLDAA---V--T-K	Y	I-S--D-P-K---F--F-R-YR-----
Pneumocystis jirovecii	CCJ29873.1	--RLD-A---M--T-K	Y	I-S--G-P-K---F--F-R-YRY----
Candida albicans	KGU13027.1	I---D-A---V-ITGK	Y	I-S--G-P-K---F--Y-R-FR-----
Taphrina deformans	CCG81999.1	I-Q-DAA---V--TGK	Y	I-S--G-P-K---F--F-R-YR-----
Pleurotus ostreatus	KDQ31819.1	I-HMD-A---L--T-K	Y	I-S--G-P-K---F--F-R-YR-----
Lichtheimia corymbifera	CDH54623.1	K-HVDAA--MM--TNK	Y	I-S--T-P-K---F--Y-R-YR-----
Rhizophagus irregularis	ESA19204.1	F-H-DAA---V--T-K	Y	I-S--G-P-K---F--F-R-YR-----
Batrachochytrium dendrobatidis	XP_006675062.1	S-HVD-AE--L--TGK	Y	V-S--G-P-K---F--Y-Q-YR-----

Tetrapisispora blattae	XP_004182131.1	---LD-A---V--T-K Y I-S--N-P-K---F--Y-R-YRY----
Lachancea nothofagi	SCV03977.1	---LD-A---M--T-K Y I-S--N-P-K---F--F-R-YKY----
Kazachstania naganishii	XP_022462372.1	---LD-A---V--T-K Y I-S--N-P-K---F--Y-R-YRY----
Naumovozyma castellii	XP_003674750.1	----D-A---V--T-K Y I-S--N-P-K---F--Y-R-YKY----
Paraphaeosphaeria sporulosa	XP_018032922.1	I--LD-A---V--T-K Y I-S--G-P-K---F--F-R-YKY----
Cladophialophora bantiana	XP_016624332.1	I--LD-A---V--T-K Y I-S--G-P-K---F--F-R-YKY----
Fonsecaea multimorphosa	XP_016637750.1	I--LD-A---V--T-K Y I-S--G-P-K---F--F-R-YKY----
Exophiala oligosperma	XP_016268534.1	I--LD-A---V--T-K Y I-S--G-P-K---F--F-R-YKY----
Endocarpon pusillum	XP_007803590.1	I--LD-A---M--T-K Y I-S--G-P-K---F--F-R-YKY----
Cyphellophora europaea	XP_008713371.1	I--LD-A---M--T-K Y I-S--G-P-K---F--F-R-YKY----
Capronia epimyces	XP_007737551.1	I--LD-A---V--T-K Y I-S--G-P-K---F--F-R-YKY----
Aureobasidium melanogenum	KEQ67430.1	K--LD-A---V--T-K Y I-S--G-P-K---F--F-R-YKY----
Phialophora attae	XP_018004907.1	D--LD-A---M--T-K Y I-S--G-P-K---F--F-R-YKY----
Tuber aestivum	CUS11234.1	K--LD-A---M--T-K Y I-S--G-P-K---F--F-R-YKY----
Vanderwaltozyma polyspora	XP_001643104.1	M--LD-A---V--T-K Y I-S--H-P-K---F--Y-R-YRY----
Coniosporium apollinis	XP_007784276.1	N--LD-A---M--T-K Y I-S--G-P-K---F--F-R-YKY----
Pyrenophora teres	EFQ90510.1	T-KLD-A---V--T-K Y I-S--G-P-K---F--F-R-YKY----
Cyberlindnera jadinii	CEP23152.1	---LD-A---V--T-K Y I-S--N-P-K---F--F-R-YR-----
Pyronema omphalodes	CCX06330.1	K--LD-A---M--T-K Y I-S--G-P-K---F--F-R-YKY----
Wickerhamomyces ciferrii	XP_011276550.1	---LD-A---V--T-K Y I-S--N-P-K---F--F-R-YR-----
Torulaspora delbrueckii	XP_003682564.1	M--LD-A---V--T-R Y V-S--N-P-K---F--F-R-YKY----
Leptosphaeria maculans	XP_003841630.1	I-KLD-A---V--T-K Y I-S--G-P-K---F--F-R-YKY----
**Plants**		
Coffea canephora	CDP17283.1	--KVD-A--MI-I-GN D A-R--S-P-K---F--LTN--KYM---
Solanum tuberosum	XP_006349377.1	--KLDAA--MM-I-GD D G-R--S-P-K------L-R--R-V---
Zea mays	NP_001148043.1	M-K-DAA--MV-I-GS D A-R--S-P-K---V-FL-Q--R-M---
Lupinus angustifolius	XP_019415595.1	--QVD-A--ML-I-GD M T-R--S-P-K---F--LTQ--K-----
Oryza sativa Indica Group	EEC76033.1	--SVD-A--MIAI-GN D A-R--S-P-K---F--LTQ--R-M---
Glycine max	XP_006584672.1	--A-D-A--MLAI-GS D T-R-MS-P-K---I--LTQ--R-----
Phaseolus vulgaris	XP_007137364.1	--QVD-A--MLAI-GN D A-R--S-P-K---F--LTQ--R-M---
Vigna angularis	XP_017408067.1	--A-D-A--VLAI-GS D S-R-MS-P-K---I--LTQ--R-----
Arachis ipaensis	XP_016169794.1	--A-D-A--MLAI-GN D T-R-MS-P-K------LTQ--R-M---
Arabidopsis thaliana	AAB65487.1	M-KLDAAE-MM-I-GD D G-T-IS-P-K---I--L-H--R-V---
Physcomitrella patens	ACH90428.1	M-K-DAA--MI--TGD D A-R--V-P-K---V--L-H--R-----
Helicosporidium sp. ATCC 50920	KDD71632.1	AL-VDNAE--L--TK- G A-RR-P-P-K---V-FL-D-GR-V--S
Chlamydomonas reinhardtii	XP_001697577.1	MY--ADA--ML--GGS S A-WQ-N-P-K--CM-FL-D-ER-LV--
Bathycoccus prasinos	XP_007513534.1	DW-VDESEFML-I-G- Q A-K-MN-A-K---I-FA-T-ERY----
Monoraphidium neglectum	KIZ03092.1	A--WQ-S-MVQE---D Q A-RL-N-P-K---V-FL-A--KLLV--
Micromonas pusilla CCMP1545	XP_003058271.1	RW--DVG-FML---GD A A-R--P-P-K---V--L-H--K--V--
Elaeis guineensis	XP_019706081.1	--KVD-G--MI-I-GN D A-R--S-P-K---F--LTN--KYM---
Carica papaya	XP_021908574.1	--KVD-A--MI-I-GN D A-R--S-P-K---F--LTN--RYM---
Hevea brasiliensis	XP_021677612.1	--KVD-A--ML-I-GN D A-R--S-P-K---F--LTN--RYM---
Musa acuminata subsp. malaccen	XP_018675993.1	--KVD-A--MI-I-GN D A-R--S-P-K---F--LTN--RYM---
Vitis vinifera	XP_002267007.3	--KVD-A--MI-I-GN D A-R--S-P-K---F--LTN--RYM---
Ricinus communis	XP_002526916.1	--KVD-A--ML-I-GN D A-R--S-P-K---F--LTN--RYM---
Daucus carota subsp. sativus	XP_017254749.1	--KVD-A--ML-I-GN D A-R--S-P-K---F--LTN--KYM---
Macleaya cordata	OVA18495.1	--KVD-A--MI-I-GN D A-R--S-P-K---F--LTN--RYM---
Morus notabilis	EXB62690.1	--KVD-A--MI-I-GN D A-R--S-P-K---F--LTN--RYM---
Aquilegia coerulea	PIA60189.1	--KVD-A--MI-I-GN D A-R--S-P-K---F--LTN--RYM---
Nelumbo nucifera	XP_010276643.1	--KVD-A--MI-I-GN D A-R--S-P-K---F--LTN--RYM---
Eucalyptus grandis	KCW90045.1	--KVD-A--ML-I-GN D A-R--S-P-K---F--LTN--RYM---
Eutrema salsugineum	ESQ44381.1	--KVD-A--ML-I-GN D A-R--S-P-K---F--LTN--RYM---
Malus domestica	XP_008375376.1	--KVD-A--MI-I-GN D A-R--S-P-K---F--LTN--RYM---
Pyrus x bretschneideri	XP_009372761.1	--KVD-A--MI-I-GN D A-R--S-P-K---F--LTN--RYM---
Trema orientalis	PON98771.1	--KVD-A--MI-I-GN D A-R--S-P-K---F--LTN--RYM---
Genlisea aurea	EPS66188.1	--KVD-A--ML-I-GN D A-R--S-P-K---F--LTN--RYM---
Prunus persica	XP_020413784.1	--KVD-A--MI-I-GN D A-R--S-P-K---F--LTN--RYM---
Juglans regia	XP_018849425.1	--KVD-A--MI-I-GN D A-R--S-P-K---F--LTN--RYM---
Manihot esculenta	XP_021624297.1	--KVD-A--ML-I-GN D A-R--S-P-K---F--LTN--RYM---
Apostasia shenzhenica	PKA53367.1	--KVD-G--MI-I-GN D A-R--S-P-K---F--LTN--KYM---
Camelina sativa	XP_010427550.1	--KVD-A--ML-I-GN D A-R--S-P-K---F--LTN--RYM---
Capsella rubella	XP_023638231.1	--KVD-A--ML-I-GN D A-R--S-P-K---F--LTN--RYM---
Cephalotus follicularis	GAV61641.1	--KVD-A--ML-I-GN D A-R--S-P-K---F--LTN--RYM---
Sesamum indicum	XP_011088692.1	--KVD-A--ML---GN D A-R--S-P-K---F--LTN--KYM---
Parasponia andersonii	PON53305.1	--KVD-A--MI-I-GN D A-R--S-P-K---F--LTN--RYM---
Sorghum bicolor	OQU92574.1	--KVD-A--ML---GN D A-R--S-P-K---F--LTN--RYM---
Raphanus sativus	XP_018489538.1	--KVD-A--ML-I-GN D A-R--S-P-K---F--LTN--RYM---
Panicum hallii	PAN49615.1	--KVD-A--ML---GN D A-R--S-P-K---F--LTN--RYM---
Solanum lycopersicum	XP_004230026.1	--KVD-A--MI-I-GN D A-R--S-P-K---F--LTN--KYM---
Ziziphus jujuba	XP_015902998.1	--KVD-A--ML-I-GN D A-R--S-P-K---F--LTN--RYM---
Brassica napus	XP_013721154.2	--KVD-A--ML-I-GN D A-R--S-P-K---F--LTN--RYM---
Dorcoceras hygrometricum	KZV47292.1	--KVD-A--ML-I-GN D A-R--S-P-K---F--LTN--RYM---

Erythranthe guttata	XP_012830685.1	--KVD-G--MM-I-GN	D	A-R--S-P-K---F--LTN--KYM---	
Capsicum annuum	XP_016557282.1	--KVD-A--MI-I-GN	D	A-R--S-P-K---F--LTN--KYM---	
Trifolium pratense	PNY15056.1	--KVD-A--MI-I-GN	D	A-R--S-P-K---F--LTH--RYM---	
Ipomoea nil	XP_019151708.1	--KVD-A--MI-I-GN	D	A-R--S-P-K---F--LTN--KYM---	
Fragaria vesca subsp. vesca	XP_004307106.1	--KVD-A--MI---GN	D	A-R--S-P-K---F--LTN--RYM---	
Cajanus cajan	XP_020240276.1	--KVD-A--MI---GN	D	A-R--S-P-K---F--LTN--RYM---	
**AMOEBOZOA**					
Cavenderia fasciculata	XP_004359026.1	R---DQA-F-V---*-	N	A-R--PTP-K-----FF-H-MK-----	*NTLKNG
Tieghemostelium lacteum	KYQ96762.1	----DTA-FMV---*-	N	A-R--PTP-K-----FF-H-MK--L--	*NTLKNG
Heterostelium album	XP_020433305.1	Q---DQA-F-V---*-	N	A-R--PTP-K-----FF-H-MK-----	*NTLKNG
Dictyostelium discoideum	XP_647148.1	----DTA-FMV---*-	N	A-R--PTP-K-----FF-H-MK-----	*NTLKNG
Acanthamoeba castellanii	XP_004336615.1	H---EA----I--TGD	Y	I-S--V-P-Q--QF--F-Y-LR-ML--	
**APICOMPLEXA**					
Leishmania infantum	XP_001468654.1	----K-Q-FMI-M-DK	R	WHSI PTP-K-AAQLFFCGR DWV---	
Toxoplasma gondii	EPT29018.1	R-H-SSET-VR-VGP-	Q	*-S--V-E-R--A---YTA-GK-M---	*LLGNLLLGNLSS
Plasmodium vivax	KMZ89198.1	-Y---SKE-IS-VGP-	Q	*-S--L-E-K------FT-NGKY----	*VISNMVLGNLST
Plasmodium falciparum	ETW21088.1	FY--KSKE--T-VGP-	Q	*-S--L-E-K------FT-NGKY----	*VISNMVLGNLST
**OTHERS EUKARYOTES**					
Naegleria gruberi	XP_002670454.1	R---DASN--VC--H-	L	S-SI-GTP-K--A--FF-A-MQYML--	
Fonticula alba	XP_009497027.1	F-H-D-A--IM--TGR	Y	I-S--G-P-K---F--F-A-YRY----	
Ectocarpus siliculosus	CBJ28352.1	----DEAS-MN-VAGD	Y	DYL--ITNSK---F-FY-H-QKY---N	
Ostreococcus tauri	XP_003080505.1	RWNVD-A-FVL---GD	Q	A-R--A-P-K---V----H--K-----	
Saprolegnia parasitica	XP_012202766.1	R-D-DSA---VT--GD	F	NY--FM-NSK--QF-FY-H-GR-M---	
Phytophthora infestans	XP_002897706.1	R---DLA--MLT-SGD	F	NF--FM-NSK--QF-FY-H-GR-M---	
Thecamonas trahens	XP_013760701.1	SIFLNE-E--L--TR-	N	A-TTMA-P-K--AS--F-N-LR-V---	
Thecamonas trahens	XP_013753394.1	-A-VE-A-IIAR-NAA	D	*IHAHF-AA----F-CK-P-GL-VV--	*YLTGA
Giardia intestinalis	ESU45894.1	KDSLD-QSL-KEFVTC	T	HPGVMK-P-K-PTF-VYGESGKYL--Q	
Emiliania huxleyi CCMP1516	XP_005786295.1	R---SSQ--ML--T--	Y	V-V-MFTNSK---F-FY-A-YR-VL--	
Trichomonas vaginalis G3	XP_001579530.1	F--VSDVE--V--T-D	Y	MMS--KTI-R-SAM--YTW-GRYV---	
Trichomonas vaginalis G3	XP_001583013.1	---YSF-TFC EILK	K	GKSSITFG-K-NAQ-F-TD-GR-L---	

Figure S5. Detailed species distribution information for the 1 aa conserved deletion in PIP5Kα shown in Figure 5.

		385		427	
**Mammals**					
Homo sapiens-Alpha-5K	NP_001129108.1	DGDTVSVHRPGFYAERFQRFMCNTVFKKIP		LKPSPSKKFRSGS	
Pongo abelii-Alpha-5K	XP_009242802.1	------------------------------		-------------	
Gorilla gorilla gorilla-Alpha-5K	XP_018890710.1	------------------------------		-------------	
Rhinopithecus roxellana-Alpha-5K	XP_010385509.1	------------------------------		-------------	
Piliocolobus tephrosceles-Alpha-5K	XP_023069451.1	------------------------------		-------------	
Cebus capucinus imitator-Alpha-5K	XP_017401774.1	------------------------------		-------------	
Callithrix jacchus-Alpha-5K	ABY82090.1	------------------------------		-------------	
Dasypus novemcinctus-Alpha-5K	XP_012378081.2	----------------------S-------		-------------	
Otolemur garnettii-Alpha-5K	XP_023375185.1	------------------------A-----		-------------	
Propithecus coquereli-Alpha-5K	XP_012514222.1	------------------------------		-Q-WK-L-LQNQ-	
Chlorocebus sabaeus-Alpha-5K	XP_007975432.1	------------------------------		-Q-WK-L-LQ-Q-	
Macaca nemestrina-Alpha-5K	XP_011767619.1	------------------------------		-Q-WK-L-LQ-Q-	
Galeopterus variegatus-Alpha-5K	XP_008582482.1	------------------------------		FQ-WK-L-LQ-Q-	
Enhydra lutris kenyoni-Alpha-5K	XP_022381744.1	------------------------------		-Q-*K-L-LQNQ-	*SW
Ailuropoda melanoleuca-Alpha-5K	XP_019655316.1	------------------------------		-Q-*K-L-LQ-Q-	*SW
Panthera pardus-Alpha-5K	XP_019288329.1	------------------------------		FQS*K-L-LQ-Q-	*SW
Acinonyx jubatus-Alpha-5K	XP_014933058.1	------------------------------		FQS*K-L-LQ-Q-	*SW
Balaenoptera acutorostrata-Alpha-5K	XP_007178100.1	----------S-----------T-------		SQ-*K-M--Q-Q-	*SW
Ovis aries-Alpha-5K	XP_012036418.1	----------S-----------T-------		FQ-*KNM--Q-Q-	*SW
Bos taurus-Alpha-5K	XP_005203884.1	----------S-----------T-------		FQ-*KNM--Q-Q-	*SW
Odocoileus virginianus texanus-Alpha-5K	XP_020730051.1	----------S-----------T-------		FQ-*KNM--Q-Q-	*SW
Physeter catodon-Alpha-5K	XP_007130523.1	----------S-----------T------L		SQ-*K-M--Q-Q-	*SW
**Birds**					
Sturnus vulgaris-Alpha	XP_014748828.1	----------S-----------H---R---		-------RS-A-V	
Serinus canaria-Alpha	XP_018781091.1	----------S-----------H---RR--		---PRDPGLTRFP	
Numida meleagris-Alpha-5K	XP_021232499.1	----------S-------------------		--------S---T	
Meleagris gallopavo-Alpha-5K	XP_010722285.1	----------S-------------------		--------S---T	
Coturnix japonica-Alpha-5K	XP_015740163.1	----------S-------------------		--------S---T	
Chaetura pelagica-Alpha-5K	XP_010001104.1	----------S-------------------		--------S---T	
Columba livia-Alpha-5K	XP_021136061.1	----------S-------------------		--------S---T	
Gallus gallus-Alpha-5K	NP_001135912.2	----------S-------------------		--------S---T	
Meleagris gallopavo-Alpha-5K	XP_010722283.1	----------S-------------------		--------S---T	
Antrostomus carolinensis-Alpha-5K	XP_010163162.1	----------S-------------A-----		--------S---T	
Melopsittacus undulatus-Alpha-5K	XP_012985591.1	----------S-------------A-----		--------S---T	
Haliaeetus leucocephalus-Alpha-5K	XP_010563784.1	----------S-------------A-----		--------S---T	
Aquila chrysaetos canadensis-Alpha-5K	XP_011596815.1	----------S-------------A-----		--------S---T	
Apteryx australis mantelli-Alpha-5K	XP_013798562.1	----------S-------------A-----		--------S---T	
Anas platyrhynchos-Alpha-5K	XP_021132788.1	----------S-------------A-----		--------S---T	
Anser cygnoides domesticus-Alpha-5K	XP_013056952.1	----------S-------------A-----		--------S---T	
Nipponia nippon-Alpha-5K	XP_009465378.1	----------S-------------A-----		--------S---T	
Amazona aestiva-Alpha-5K	KQL59296.1	----------S-------------A-----		--------S-C-T	
**Reptiles**					
Gekko japonicus-Alpha-5K	XP_015279999.1	----------S------------M------		--------S---V	
Anolis carolinensis-Alpha-5K	XP_008120464.1	----------S------------V------		--S-----S---A	
Pogona vitticeps-Alpha-5K	XP_020653819.1	----------S------------M------		--S-----S---A	
Python bivittatus-Alpha-5K	XP_015744819.1	----------S-------Q---HA------		--S-----S---M	
Protobothrops mucrosquamatus-Alpha-5K	XP_015680374.1	----------S-----------QA------		--S-----S---M	
**Amphibians**					
Xenopus tropicalis-Alpha-5K	OCA14230.1	--------------D---K---SI------		--T-----S-TMP	
Xenopus laevis-Alpha-5K	OCT69594.1	--------------D---K---VS------		--T-----S-TMP	
Nanorana parkeri-Alpha-5K	XP_018421916.1	------------------K---A-------		--A-----S-TMP	
**Fishes**					
Danio rerio-Alpha-5K	NP_001018438.1	----------S---D---K---S---R-SQ		--T----RS-L-P	
Poecilia formosa-Alpha-5K	XP_007556196.1	------------------Q-----------		--------S-G-G	
Maylandia zebra-Alpha-5K	XP_004541373.2	------------------Q-----------		--------S-G-G	
Neolamprologus brichardi-Alpha-5K	XP_006804986.1	------------------Q-----------		--------S-G-G	
Acanthochromis polyacanthus-Alpha-5K	XP_022051707.1	------------------Q-----------		--------S-G-G	
Pundamilia nyererei-Alpha-5K	XP_005732196.1	------------------Q-----------		--------S-G-G	
Oryzias latipes-Alpha-5K	XP_004078182.1	------------------Q-----------		--------S-G-G	
Amphiprion ocellaris-Alpha-5K	XP_023117624.1	------------------Q-----------		--------S-G-G	
Xiphophorus maculatus-Alpha-5K	XP_023186755.1	------------------Q-----------		--------S-G-G	
Cyprinodon variegatus-Alpha-5K	XP_015248386.1	------------------Q-----------		--------S-G-G	
Oreochromis niloticus-Alpha-5K	XP_005472748.1	------------------Q-----------		--------S-G-G	
Fundulus heteroclitus-Alpha-5K	XP_012721150.1	------------------Q-----------		--------S-G-G	
Monopterus albus-Alpha-5K	XP_020462945.1	------------------Q-----------		--------S-G-G	

Cynoglossus semilaevis-Alpha-5K	XP_008321275.1	------------------Q----------- --------S-G-G
Tetraodon nigroviridis-Alpha-5K	CAG11890.1	------------------Q----------- --------S-G-G
Esox lucius-Alpha-5K	XP_012994913.1	------------------Q----------- -R------S-G-G
Labrus bergylta-Alpha-5K	XP_020497624.1	--------------D---Q----------- --------S-G-G
Austrofundulus limnaeus-Alpha-5K	XP_013885096.1	--------------D---Q----------- --------S-G-G
Stegastes partitus-Alpha-5K	XP_008285964.1	------------------Q-----A----- --------S-G-G
Nothobranchius furzeri-Alpha-5K	XP_015806121.1	------------------Q----------- --------S---G
Oncorhynchus mykiss-Alpha-5K	CDQ97059.1	------------------Q----------- --L-----S-G-G
Paralichthys olivaceus-Alpha-5K	XP_019948859.1	------------------Q----------- --------S---G
Lates calcarifer-Alpha-5K	XP_018541718.1	------------------Q---S------- --------S-G-G
Seriola dumerili-Alpha-5K	XP_022621881.1	------------------Q---S------- --------S-G-G
Salmo salar-Alpha-5K	XP_014057413.1	------------------Q----------- --L-----S-G-G
Clupea harengus-Alpha-5K	XP_012684664.1	--------------------------R--- --------S-G-C
Boleophthalmus pectinirostris-Alpha-5K	XP_020783608.1	------------------Q-------R--- --A-----S---G
Pygocentrus nattereri-Alpha-5K	XP_017555944.1	----------------------S---R-A --S-----S-G-C
Astyanax mexicanus-Alpha-5K	XP_022529206.1	--------------------------R-A --S-----SQR-C
Homo sapiens-Beta	AAC50914.1	----------S---D--LK--NSR-----Q A --A-----RCNSI
Rattus norvegicus-Beta	NP_001012761.1	----------S---D--LK--NSR-----Q A --A-----RCNSI
Sturnus vulgaris-Beta	XP_014725312.1	----------S---D--LK--NAR----VQ A --------RCNSI
Serinus canaria-Beta	XP_009092185.1	----------S---D--LK--NAR----VQ A --------RCNSI
Xenopus tropicalis-Beta	XP_012827258.1	---S------S---D--LK--NAR----VQ A --T-----RCNSI
Nanorana parkeri-Beta	XP_018411161.1	----------S---G--LK--NSK----VQ A --S----RRCNSI
Xenopus laevis-Beta	AAH55973.1	---S------C---D--LK--NSR----VQ A --A-----RGNSI
Python bivittatus-Beta	XP_007439496.2	----------S---D--LK--STR----NQ T --S-----RCNSI
Protobothrops mucrosquamatus-Beta	XP_015679138.1	----------S---D--LK--STR----NQ I --S-----RCNSI
Danio rerio-Beta	NP_001004579.1	----------S---N--LK--SSR--R-NQ P NRF-----A-NSI
Maylandia zebra-Beta	XP_004538536.1	----------S---D--LK--GT-----SH P -RGAS--RKKNSL
Pundamilia nyererei-Beta	XP_005722354.1	---S----------S--LK--STR--R-TQ P IRF----RT-TSI
Homo sapiens-Gamma	XP_011526148.1	----------S------FK--S----R-NS S --S-----G-G-A
Rattus norvegicus-Gamma	NP_001009967.2	----------S------FK--SS---R-SS S --S-----G-GAL
Protobothrops mucrosquamatus-Gamma	XP_015666168.1	----------S------FK--T----R-SS S --A-----G-NAL
Python bivittatus-Gamma	XP_007441380.1	----------S------FK--T----R-SS S --A-----G--AL
Serinus canaria-Gamma	XP_018777315.1	----------S------FK--T----R-NS S --S-----G--AL
Sturnus vulgaris-Gamma	XP_014739865.1	----------S------FK--T----R-NS S --S-----G--AL
Xenopus tropicalis-Gamma	XP_017946547.1	----------S------FK--T----R-TS S --S-----G--AL
Maylandia zebra-Gamma	XP_004554931.1	--------------D--LK--ST-----SS S --S-----G-VSL
Pundamilia nyererei-Gamma	XP_005734030.1	--------------D--LK--ST-----SS S --S-----G-VSL

Mammals

Homo sapiens-Beta-5K	EAW62466.1	----------S---D--LK--NSR-----Q A --A-----RCNSI
Carlito syrichta-Beta-5K	XP_008053854.1	----------S---D--LK--NSR-----Q A --A-----RCNSI
Marmota marmota marmota-Beta-5K	XP_015348322.1	----------S---D--LK--NSR-----Q A --A-----RCNSI
Orcinus orca-Beta-5K	XP_004276469.1	----------S---D--LK--NSR-----Q A --A-----RCNSI
Pongo abelii-Beta-5K	PNJ81438.1	----------S---D--LK--NSR-----Q A --A-----RCNSI
Mandrillus leucophaeus-Beta-5K	XP_011825535.1	----------S---D--LK--NSR-----Q A --A-----RCNSI
Nomascus leucogenys-Beta-5K	XP_003273915.1	----------S---D--LK--NSR-----Q A --A-----RCNSI
Felis catus-Beta-5K	XP_019671300.2	----------S---D--LK--NSR-----Q A --A-----RCNSI
Tursiops truncatus-Beta-5K	XP_019790298.1	----------S---D--LK--NSR-----Q A --A-----RCNSI
Equus przewalskii-Beta-5K	XP_008510987.1	----------S---D--LK--NSR-----Q A --A-----RCNSI
Colobus angolensis palliatus-Beta-5K	XP_011804275.1	----------S---D--LK--NSR-----Q A --A-----RCNSI
Gorilla gorilla gorilla-Beta-5K	XP_018889556.1	----------S---D--LK--NSR-----Q A --A-----RCNSI
Lipotes vexillifer-Beta-5K	XP_007466832.1	----------S---D--LK--NSR-----Q A --A-----RCNSI
Pan paniscus-Beta-5K	XP_003824805.1	----------S---D--LK--NSR-----Q A --A-----RCNSI
Pan troglodytes-Beta-5K	XP_001137535.2	----------S---D--LK--NSR-----Q A --A-----RCNSI
Propithecus coquereli-Beta-5K	XP_012497079.1	----------S---D--LK--NSR-----Q A --A-----RCNSI
Pantholops hodgsonii-Beta-5K	XP_005976403.1	----------S---D--LK--NSR-----Q A --A-----RCNSI
Macaca mulatta-Beta-5K	EHH24152.1	----------S---D--LK--NSR-----Q A --A-----RCNSI
Ursus maritimus-Beta-5K	XP_008689163.1	----------S---D--LK--NSR-----Q A --A-----RCNSI
Eptesicus fuscus-Beta-5K	XP_008143183.1	----------S---D--LK--NSR-----Q A --A-----RCNSI
Myotis davidii-Beta-5K	ELK29070.1	----------S---D--LK--NSR-----Q A --A-----RCNSI
Myotis brandtii-Beta-5K	XP_014384682.1	----------S---D--LK--NSR-----Q A --A-----RCNSI
Vicugna pacos-Beta-5K	XP_015105804.1	----------S---D--LK--NSR-----Q A --A-----RCNSI
Camelus bactrianus-Beta-5K	XP_010966211.1	----------S---D--LK--NSR-----Q A --A-----RCNSI
Camelus dromedarius-Beta-5K	XP_010998086.1	----------S---D--LK--NSR-----Q A --A-----RCNSI
Saimiri boliviensis boliviensi-Beta-5K	XP_010344445.1	----------S---D--LK--NSR-----Q A --A-----RCNSI
Rhinopithecus roxellana-Beta-5K	XP_010361273.1	----------S---D--LK--NSR-----Q A --A-----RCNSI
Cebus capucinus imitator-Beta-5K	XP_017398912.1	----------S---D--LK--NSR-----Q A --A-----RCNSI
Cercocebus atys-Beta-5K	XP_011912030.1	----------S---D--LK--NSR-----Q A --A-----RCNSI
Papio anubis-Beta-5K	XP_003911826.1	----------S---D--LK--NSR-----Q A --A-----RCNSI
Physeter catodon-Beta-5K	XP_023982630.1	----------S---D--LK--NSR-----Q A --A-----RCNSI
Sus scrofa-Beta-5K	XP_020920288.1	----------S---D--LK--NSR-----Q A --A-----RCNSI
Callithrix jacchus-Beta-5K	XP_008994234.1	----------S---D--LK--NSR-----Q A --A-----RCNSI
Rhinopithecus bieti-Beta-5K	XP_017728533.1	----------S---D--LK--NSR-----Q A --A-----RCNSI

Canis lupus familiaris-Beta-5K	XP_005615942.1	----------S---D--LK--NSR-----Q A --A-----RCNSI
Chlorocebus sabaeus-Beta-5K	XP_007967617.1	----------S---D--LK--NSR-----Q A --A-----RCNSI
Delphinapterus leucas-Beta-5K	XP_022429264.1	----------S---D--LK--NSR-----Q A --A-----RCNSI
Myotis lucifugus-Beta-5K	XP_023620347.1	----------S---D--LK--NSR-----Q A --A-----RCNSI
Panthera pardus-Beta-5K	XP_019319198.1	----------S---D--LK--NSR-----Q A --A-----RCNSI
Erinaceus europaeus-Beta-5K	XP_016046043.1	----------S---D--LK--NSR-----Q A --A-----RCNSI
Panthera tigris altaica-Beta-5K	XP_015390909.1	----------S---D--LK--NSR-----Q A --A-----RCNSI
Galeopterus variegatus-Beta-5K	XP_008578886.1	----------S---D--LK--NSR-----Q A --A-----RCNSI
Macaca fascicularis-Beta-5K	EHH57441.1	----------S---D--LK--NSR-----Q A --A-----RCNSI
Balaenoptera acutorostrata-Beta-5K	XP_007182303.1	----------S---D--LK--NSR-----Q A --A-----RCNSI
Manis javanica-Beta-5K	XP_017509139.1	----------S---D--LK--NSR----VQ A --A-----RCNSI
Oryctolagus cuniculus-Beta-5K	XP_008254412.1	----------S---D--LK--NSR-----Q V --A-----RCNSI
Condylura cristata-Beta-5K	XP_004677673.1	----------S---D--LK--NSR-----Q A --A-----RC-SI
Macaca nemestrina-Beta-5K	XP_011756750.1	----------S---D--LK--NSR-----Q A F-AHLLRERCNSI

Birds

Haliaeetus albicilla-Beta-5K	KFQ07119.1	----------S---D--LK--NTR----VQ A --S-----RCNSI
Manacus vitellinus-Beta-5K	XP_017936501.1	----------S---D--LK--STR----VQ A --S-----RCNSI
Chlamydotis macqueenii-Beta-5K	XP_010124567.1	----------S---D--LK--NTR----VQ A --S-----RCNSI
Falco peregrinus-Beta-5K	XP_005242231.1	----------S---D--LK--NTR-----Q A --S-----RCNSI
Callipepla squamata-Beta-5K	OXB62686.1	----------S---D--LK--NSR----VQ A -RS-----RCNSI
Sturnus vulgaris-Beta-5K	XP_014725312.1	----------S---D--LK--NAR----VQ A --------RCNSI
Numida meleagris-Beta-5K	XP_021236396.1	----------S---D--LK--NTR----VQ A -RS-----RCNSI
Taeniopygia guttata-Beta-5K	XP_002191336.1	----------S---D--LK--NAR----VQ A --------RCNSI
Haliaeetus leucocephalus-Beta-5K	XP_010566885.1	----------S---D--LK--NTR----VQ A --S-----RCNSI
Geospiza fortis-Beta-5K	XP_005420909.1	----------S---D--LK--NAR----VQ A --------RCNSI
Corvus brachyrhynchos-Beta-5K	XP_017586100.1	----------S---D--LK--NAR----VQ A --------RCNSI
Zonotrichia albicollis-Beta-5K	XP_005491000.1	----------S---D--LK--NAR----VQ A --------RCNSI
Merops nubicus-Beta-5K	KFQ22315.1	----------S---D--LK--STR----VQ A --S-----RCNSI
Serinus canaria-Beta-5K	XP_009092185.1	----------S---D--LK--NAR----VQ A --------RCNSI
Lonchura striata domestica-Beta-5K	OWK55566.1	----------S---D--LK--NAR----VQ A --------RCNSI
Phaethon lepturus-Beta-5K	KFQ71669.1	----------S---D--LK--STR----VQ A --S-----RCNSI
Apaloderma vittatum-Beta-5K	XP_009864634.1	----------S---D--LK--STR----VQ A --S-----RCNSI
Meleagris gallopavo-Beta-5K	XP_010724018.1	----------S---D--LK--NTR----VQ A -RS-----RCNSI
Pseudopodoces humilis-Beta-5K	XP_005522707.1	----------S---D--LK--NTR----VQ A -R------RCNSI
Struthio camelus australis-Beta-5K	XP_009670362.1	----------S---D--LK--STR----VQ A --T-----RCNSI
Parus major-Beta-5K	XP_015508926.1	----------S---D--LK--NTR----VQ A -R------RCNSI
Gallus gallus-Beta-5K	XP_015135771.1	----------S---D--LK--NTR----VQ A -RS-----RCNSI
Pterocles gutturalis-Beta-5K	XP_010081563.1	----------S---D--LK--STR----VQ A --S-----QCNSI
Lepidothrix coronata-Beta-5K	XP_017661575.1	----------S---D--LK--STR----VQ A --S-----RCNSI
Mesitornis unicolor-Beta-5K	XP_010183780.1	----------S---D--LK--STR----VQ A -RS-----RCNSI
Coturnix japonica-Beta-5K	XP_015704584.1	---A------S---D--LK--NTR----VQ A --S-----RCNSI
Nipponia nippon-Beta-5K	XP_009458847.1	----------S---D--LK--STR----VQ A --S-----RCNSI
Anas platyrhynchos-Beta-5K	XP_021123700.1	----------S---D--LK--STR----VQ A -RS-----RCNSI
Pygoscelis adeliae-Beta-5K	XP_009326352.1	----------S---D--LK--STR----VQ A --S-----RCNSI
Calidris pugnax-Beta-5K	XP_014807148.1	----------S---D--LK--STR----VQ A --S-----RCNSI
Aptenodytes forsteri-Beta-5K	XP_009283561.1	----------S---D--LK--STR----VQ A --S-----RCNSI
Aquila chrysaetos canadensis-Beta-5K	XP_011576030.1	----------S---D--LK--STR----VQ A --S-----RCNSI
Picoides pubescens-Beta-5K	KFV72101.1	----------S---D--LK--STK----VQ A --------RCNSI
Egretta garzetta-Beta-5K	KFP13705.1	----------S---D--LK--STR----FQ A --S-----RCNSI
Buceros rhinoceros silvestris-Beta-5K	XP_010138959.1	----------S---D--LK--STR----VH A --T-----RCNSI
Opisthocomus hoazin-Beta-5K	KFR14126.1	----------S---D--LK--STR----VQ A --S-----QCNSI
Anser cygnoides domesticus-Beta-5K	XP_013049494.1	----------S---D--LK--STR----LQ A -RS-----RCNSI
Tinamus guttatus-Beta-5K	XP_010224625.1	---S------S---D--LK--STR----VQ A -RT-----RCNSI
Podiceps cristatus-Beta-5K	KFZ69294.1	---A------S---D--LK--STR----LQ A --S-----RCNSI
Chaetura pelagica-Beta-5K	KFU85810.1	----------S---D--LK--STK--R-VQ A --S-----RCNSI
Cuculus canorus-Beta-5K	KFO79437.1	----------S---D--LK--STR----VQ G --S-----WCNSI

Reptiles

Gekko japonicus-Beta-5K	XP_015261333.1	----------S---D--LK--SSR----NQ A --S-----RCNSI
Anolis carolinensis-Beta-5K	XP_008101519.1	----------S---D--LK--SSR----NQ T --S-----RCNSI
Pogona vitticeps-Beta-5K	XP_020661323.1	----------S---D--LK--SSR----NQ T --S-----RCNSI
Python bivittatus-Beta-5K	XP_007439496.2	----------S---D--LK--STR----NQ T --S-----RCNSI
Protobothrops mucrosquamatus-Beta-5K	XP_015679138.1	----------S---D--LK--STR----NQ I --S-----RCNSI
Thamnophis sirtalis-Beta-5K	XP_013907811.1	----------S---D--LK--STR----NQ L --S-----RCNSI
Ophiophagus hannah-Beta-5K	ETE59205.1	----------S---D--LK--STR----NQ * --S-----RCNSI *SRDEGWLV

Amphibians

Xenopus tropicalis-Beta-5K	XP_012827258.1	---S------S---D--LK--NAR----VQ A --T-----RCNSI
Nanorana parkeri-Beta-5K	XP_018411161.1	----------S---G--LK--NSK----VQ A --S----RRCNSI
Xenopus laevis-Beta-5K	AAH55973.1	---S------C---D--LK--NSR----VQ A --A-----RGNSI

Fishes

Maylandia zebra-Beta-5K	XP_004538536.1	----------S---D--LK--GT-----SH P -RGAS--RKKNSL
Pundamilia nyererei-Beta-5K	XP_005725614.1	----------S---D--LK--GT-----SH P -RGAS--RKKNSL

Neolamprologus brichardi-Beta-5K	XP_006803535.1	----------S---D--LK--GT-----SH P -RGAS--RKKNSL
Oreochromis niloticus-Beta-5K	XP_003440259.2	----------S---D--LK--GT-----SH P -RGAS--RKKNSL
Haplochromis burtoni-Beta-5K	XP_005921766.1	----------S---D--LK--GT-----SH P -RGAS--RKKNSL
Takifugu rubripes-Beta-5K	XP_011618834.1	----------S---D--LK--GS------H P -RGAS--RKKGSL
Cynoglossus semilaevis-Beta-5K	XP_016894343.1	----------S---D--L---GT----R-H P -RGAS---KKNSL
Acanthochromis polyacanthus-Beta-5K	XP_022072780.1	----------S---D--LK--GS------H P -RGTS--RK-NSL
Paralichthys olivaceus-Beta-5K	XP_019960688.1	----------S---D--LK--GS------H P -RGAS--RK--SL
Kryptolebias marmoratus-Beta-5K	XP_017288436.1	----------S---D--LK--GT-----LH P -RGAS-RRK-GSL
Amphiprion ocellaris-Beta-5K	XP_023138083.1	----------N---D--LK--GS------H P -RGTS--RK-NSL
Lates calcarifer-Beta-5K	XP_018558033.1	----------S---D--LK--GS-----LH P -RGAS--RK-GSL
Oryzias melastigma-Beta-5K	XP_024154386.1	----------S---D--LK--GT-----AH A -RGAS-RRKK-SL
Notothenia coriiceps-Beta-5K	XP_010787650.1	----------S---D--LK--GT------H P -RGTS--RK-TSM
Xiphophorus maculatus-Beta-5K	XP_023193673.1	--------------D--LK--GT---R-LH P -RGAS--RK--SL
Seriola lalandi dorsalis-Beta-5K	XP_023255674.1	----------S---D--LK--ES-----SH P -RGAS-RRK--SL
Poecilia formosa-Beta-5K	XP_007575909.1	--------------D--LK--GT---R-LH P -RGAS--RK--SL
Danio rerio-Beta-5K	XP_005155628.1	----------N---D--LK--GS------H P -RGAS-RRKK-SI
Cyprinodon variegatus-Beta-5K	XP_015228657.1	--------------D--LK--GT-----LH P IRGAS-RRK--SL
Labrus bergylta-Beta-5K	XP_020491677.1	----------S---D--LN--GS-----NH P -RGAS--RK-TSF
Oryzias latipes-Beta-5K	XP_011480348.1	----------S---D--LK-LST-----AH S -RGTS--RKKNSL
Fundulus heteroclitus-Beta-5K	XP_021179699.1	----------C---D--LK--ST---R-LH P -RGAS--RK--SL
Larimichthys crocea-Beta-5K	XP_010734153.1	----------S---D--LK--GS------H S -RGAS--RKKGSV
Monopterus albus-Beta-5K	XP_020470796.1	----------S---D--LK--SSV---RLH P -RGAS---K-NSL

Mammals

Homo sapiens-Gamma-5K	AAC32904.1	----------S------FK--S----R-NS S --S-----G-G-A
Gorilla gorilla gorilla-Gamma-5K	XP_018871823.1	----------S------FK--S----R-NS S --S-----G-G-A
Cercocebus atys-Gamma-5K	XP_011928451.1	----------S------FK--S----R-NS S --S-----G-G-A
Callithrix jacchus-Gamma-5K	XP_008985238.1	----------S------FK--S----R-NS S --S-----G-G-A
Pongo abelii-Gamma-5K	XP_024093427.1	----------S------FK--S----R-NS S --S-----G-G-A
Papio anubis-Gamma-5K	XP_003914709.1	----------S------FK--S----R-NS S --S-----G-G-A
Pan troglodytes-Gamma-5K	XP_016792550.1	----------S------FK--S----R-NS S --S-----G-G-A
Cebus capucinus imitator-Gamma-5K	XP_017362897.1	----------S------FK--S----R-NS S --S-----G-G-A
Piliocolobus tephrosceles-Gamma-5K	XP_023039373.1	----------S------FK--S----R-NS S --S-----G-G-A
Saimiri boliviensis boliviensi-Gamma-5K	XP_010347757.1	----------S------FK--S----R-NS S --S-----G-G-A
Macaca mulatta-Gamma-5K	EHH29471.1	----------S------FK--S----R-NS S --S-----G-G-A
Pan paniscus-Gamma-5K	XP_008970866.1	----------S------FK--S----R-NS S --S-----G-G-A
Macaca fascicularis-Gamma-5K	XP_015295799.1	----------S------FK--S----R-NS S --S-----G-G-A
Rhinopithecus roxellana-Gamma-5K	XP_010365585.1	----------S------FK--S----R-NS S --S-----G---A
Nomascus leucogenys-Gamma-5K	XP_012358835.1	V---------S------FK--S----R-NS S --S-----G---A
Panthera pardus-Gamma-5K	XP_019286317.1	----------S------FK--S----R-NS S --S-----G-GA-
Felis catus-Gamma-5K	XP_023099540.1	----------S------FK--S----R-NS S --S-----G-GA-
Microcebus murinus-Gamma-5K	XP_020139527.1	----------S------FK--S----R-NS S --S-----G-GAL
Enhydra lutris kenyoni-Gamma-5K	XP_022381192.1	----------S------FK--S----R-NS S --S-----G-GAL
Propithecus coquereli-Gamma-5K	XP_012501604.1	----------S------FK--S----R-NS S --S-----G-GAL
Orycteropus afer afer-Gamma-5K	XP_007949279.1	----------S------FK--S----R-NS S --S-----G-GAL
Camelus ferus-Gamma-5K	XP_014410201.1	----------S------FK--S----R-NS S --S-----G-GAL
Neomonachus schauinslandi-Gamma-5K	XP_021561194.1	----------S------FK--S----R-NS S --S-----G-GAL
Loxodonta africana-Gamma-5K	XP_010599556.2	----------S------FK--S----R-NS S --S-----G-GAL
Leptonychotes weddellii-Gamma-5K	XP_006738797.1	----------S------FK--S----R-NS S --S-----G-GAL
Odobenus rosmarus divergens-Gamma-5K	XP_004395402.1	----------S------FK--S----R-NS S --S-----G-GAL
Castor canadensis-Gamma-5K	XP_020036849.1	----------S------FK--S----R-NS S --S-----G-GAL
Otolemur garnettii-Gamma-5K	XP_012659946.2	----------S------FK--S----R-NS S --S-----G-GAL
Mesocricetus auratus-Gamma-5K	XP_021080145.1	----------S------FK--SS---R-SS S --S-----G-GAL
Sarcophilus harrisii-Gamma-5K	XP_023352567.1	----------S------FK--S----R-NS S --S-----G-TTL
Galeopterus variegatus-Gamma-5K	XP_008581117.1	----------S------FK--S----R-NS S --S-----G--AL

Birds

Manacus vitellinus-Gamma-5K	XP_017939017.1	----------S------FK--T----R-NS S --S-----G--AL
Columba livia-Gamma-5K	PKK17397.1	----------S------FK--T----R-NS S --S-----G--AL
Calypte anna-Gamma-5K	XP_008496647.1	----------S------FK--T----R-NS S --S-----G--AL
Haliaeetus albicilla-Gamma-5K	XP_009926193.1	----------S------FK--T----R-NS S --S-----G--AL
Manacus vitellinus-Gamma-5K	XP_017939023.1	----------S------FK--T----R-NS S --S-----G--AL
Geospiza fortis-Gamma-5K	XP_014165085.1	----------S------FK--T----R-NS S --S-----G--AL
Gallus gallus-Gamma-5K	XP_015155249.1	----------S------FK--T----R-NS S --S-----G--AL
Colius striatus-Gamma-5K	XP_010198227.1	----------S------FK--T----R-NS S --S-----G--AL
Mesitornis unicolor-Gamma-5K	XP_010180216.1	----------S------FK--T----R-NS S --S-----G--AL
Nestor notabilis-Gamma-5K	XP_010018411.1	----------S------FK--T----R-NS S --S-----G--AL
Lonchura striata domestica-Gamma-5K	XP_021383891.1	----------S------FK--T----R-NS S --S-----G--AL
Haliaeetus albicilla-Gamma-5K	XP_009926191.1	----------S------FK--T----R-NS S --S-----G--AL
Fulmarus glacialis-Gamma-5K	XP_009580515.1	----------S------FK--T----R-NS S --S-----G--AL
Pterocles gutturalis-Gamma-5K	XP_010085086.1	----------S------FK--T----R-NS S --S-----G--AL
Pygoscelis adeliae-Gamma-5K	XP_009321283.1	----------S------FK--T----R-NS S --S-----G--AL
Serinus canaria-Gamma-5K	XP_018777312.1	----------S------FK--T----R-NS S --S-----G--AL
Melopsittacus undulatus-Gamma-5K	XP_005140981.2	----------S------FK--T----R-NS S --S-----G--AL

Manacus vitellinus-Gamma-5K	XP_017939015.1	----------S------FK--T----R-NS S --S-----G--AL
Lonchura striata domestica-Gamma-5K	XP_021383889.1	----------S------FK--T----R-NS S --S-----G--AL
Apteryx australis mantelli-Gamma-5K	XP_013798587.1	----------S------FK--T----R-NS S --S-----G--AL
**Reptiles**		
Thamnophis sirtalis-Gamma-5K	XP_013927381.1	----------S------FK--T----R-SS S --A-----G--AL
Pogona vitticeps-Gamma-5K	XP_020636870.1	----------S------FK--T----R-SS S --A-----G--AL
Python bivittatus-Gamma-5K	XP_007441380.1	----------S------FK--T----R-SS S --A-----G--AL
Anolis carolinensis-Gamma-5K	XP_008123366.1	----------S------FK--T----R-SS S --A-----G--AL
Gekko japonicus-Gamma-5K	XP_015276073.1	----------S------FK--T----R-SS S --S-----G-GAL
**Amphibians**		
Protobothrops mucrosquamatus-Gamma-5K	XP_015666168.1	----------S------FK--T----R-SS S --A-----G-NAL
Xenopus tropicalis-Gamma-5K	OCA49041.1	----------S------FK--T----R-TS S --S-----G--AL
Xenopus laevis-Gamma-5K	XP_018109526.1	-----------------FK--TH---R-TS S --S-----G--AL
Nanorana parkeri-Gamma-5K	XP_018420852.1	--------------D--FK--T-I--R-TS S --S-----G--AL
**Fishes**		
Lates calcarifer-Gamma-5K	XP_018532494.1	----------S------YK-CSTI----SC S -RS----RG-GAL
Danio rerio-Gamma-5K	XP_002666296.1	----------S---D--L---SS---R-TS S --S----RG-G-L
Cyprinodon variegatus-Gamma-5K	XP_015241514.1	----------S------FK-CSTV--R-SC S -R-----RG-GAL
Neolamprologus brichardi-Gamma-5K	XP_006786750.1	----------S------YK-CSTV----SC S -RS----RG-GVL
Oreochromis niloticus-Gamma-5K	XP_005476184.1	----------S------YK-CSTV----SC S -RS----RG-GVL
Xiphophorus maculatus-Gamma-5K	XP_005797142.2	----------S------FK-CSTV--R-SC S -R-----RG-GAL
Haplochromis burtoni-Gamma-5K	XP_005920620.1	----------S------YK-CSTV----SC S -RS----RG-GVL
Maylandia zebra-Gamma-5K	XP_012772289.1	----------S------YK-CSTV----SC S -RS----RG-GVL
Pundamilia nyererei-Gamma-5K	XP_005721179.1	----------S------YK-CSTV----SC S -RS----RG-GVL
Acanthochromis polyacanthus-Gamma-5K	XP_022046423.1	----------S------YK-CSTI--R-SN S -RS----RG-GVL
Fundulus heteroclitus-Gamma-5K	XP_012729576.1	----------S------F--CSTV--R-SC S -R-----RG-GAL
Seriola lalandi dorsalis-Gamma-5K	XP_023267354.1	-----------------YK-CSTI----SC S -RS----RG-GAL
Seriola dumerili-Gamma-5K	XP_022610606.1	-----------------YK-CSTI----SC S -RS----RG-GAL
Oryzias latipes-Gamma-5K	XP_004079345.2	-----------------YK-CSTI----SC S -RS----RG-GQL
Poecilia mexicana-Gamma-5K	XP_014856633.1	----------S------FK-CSTV--R-SC S -R------G-GAL
Poecilia formosa-Gamma-5K	XP_007571215.1	----------S------FK-CSTV--R-SC S -R------G-GAL
Poecilia latipinna-Gamma-5K	XP_014911115.1	----------S------FK-CSTV--R-SC S -R------G-GAL
Amphiprion ocellaris-Gamma-5K	XP_023121344.1	-----------------YK-CSTI--R-SN S -RS----RG-GVL
Kryptolebias marmoratus-Gamma-5K	XP_017277796.1	----------S------YK-CSSV----SC S -RS----RG-GTF
Poecilia reticulata-Gamma-5K	XP_017165352.1	----------S------F--CSTV--R-SC S -R------G-GAL
Stegastes partitus-Gamma-5K	XP_008304116.1	-----------------YK-CSTI--R-SC S -RS----RG-GVL
Austrofundulus limnaeus-Gamma-5K	XP_013876251.1	----------S------Y--CSTV----SC S -RS-- -RG-GTF

Figure S6. Detailed species distribution information for the 2 aa conserved insert in PIP5Kβ isoform shown in Figure 6.

		250		301
**Mammals**				
Homo sapiens-Beta-5K	EAW62466.1	LYSTAMESIQGPGKSGDGI	IT	ENPDTMGGIPAKSHRGEKLLLFMGIIDILQS
Myotis brandtii-Beta-5K	XP_014384682.1	-------------------	--	-------------------------------
Carlito syrichta-Beta-5K	XP_008053854.1	-------------------	--	--------------K----------------
Pan paniscus-Beta-5K	XP_003824805.1	-------------------	--	-------------------------------
Pan troglodytes-Beta-5K	XP_001137535.2	-------------------	--	-------------------------------
Gorilla gorilla gorilla-Beta-5K	XP_018889556.1	-------------------	--	-------------------------------
Chlorocebus sabaeus-Beta-5K	XP_007967617.1	-------------------	--	-------------------------------
Myotis lucifugus-Beta-5K	XP_023620347.1	-------------------	--	-------------------------------
Rhinopithecus bieti-Beta-5K	XP_017728533.1	-------------------	--	-------------------------------
Ailuropoda melanoleuca-Beta-5K	XP_019664190.1	-------------------	--	--------------K----------------
Mandrillus leucophaeus-Beta-5K	XP_011825535.1	-------------------	--	-------------------------------
Pongo abelii-Beta-5K	PNJ81438.1	-------------------	--	-------------------------------
Nomascus leucogenys-Beta-5K	XP_003273915.1	-------------------	--	-------------------------------
Macaca fascicularis-Beta-5K	EHH57441.1	-------------------	--	-------------------------------
Colobus angolensis palliatus-Beta-5K	XP_011804275.1	-------------------	--	-------------------------------
Macaca mulatta-Beta-5K	EHH24152.1	-------------------	--	-------------------------------
Canis lupus familiaris-Beta-5K	XP_005615942.1	-------------------	--	--------------K----------------
Callithrix jacchus-Beta-5K	XP_008994234.1	-------------------	--	-------------------------------
Cebus capucinus imitator-Beta-5K	XP_017398912.1	-------------------	--	-------------------------------
Camelus bactrianus-Beta-5K	XP_010966211.1	-------------------	--	-------------------------------
Cercocebus atys-Beta-5K	XP_011912030.1	-------------------	--	-------------------------------
Rhinopithecus roxellana-Beta-5K	XP_010361273.1	-------------------	--	-------------------------------
Vicugna pacos-Beta-5K	XP_015105804.1	-------------------	--	-------------------------------
Papio anubis-Beta-5K	XP_003911826.1	-------------------	--	-------------------------------
Myotis davidii-Beta-5K	ELK29070.1	-------------------	--	-------------------------------
Balaenoptera acutorostrata-Beta-5K	XP_007182303.1	-------------------	--	--------------K----------------
Macaca nemestrina-Beta-5K	XP_011756750.1	-------------------	--	-------------------------------
Orcinus orca-Beta-5K	XP_004276469.1	-------------------	--	--------------K----------------
Lipotes vexillifer-Beta-5K	XP_007466832.1	-------------------	--	--------------K----------------
Ursus maritimus-Beta-5K	XP_008689163.1	-------------------	--	--------------K----------------
Tursiops truncatus-Beta-5K	XP_019790298.1	-------------------	--	--------------K----------------
Cricetulus griseus-Beta-5K	ERE80811.1	---------------A---	-A	--------------K----------------
Delphinapterus leucas-Beta-5K	XP_022429264.1	-------------------	V-	--------------K----------------
Castor canadensis-Beta-5K	XP_020014191.1	--------------P---L	V-	--------------K----------------
Manis javanica-Beta-5K	XP_017509139.1	-------------------	--	--------------K----------------
Propithecus coquereli-Beta-5K	XP_012497079.1	-------------------	--	--------------K----------------
Pantholops hodgsonii-Beta-5K	XP_005976403.1	-------------------	--	--------------K----------------
Ictidomys tridecemlineatus-Beta-5K	XP_021581313.1	-------------------	-A	--------------K----------------
Sus scrofa-Beta-5K	XP_020920288.1	-------------------	--	--------------K----------------
Oryctolagus cuniculus-Beta-5K	XP_008254412.1	-------------------	--	--------------K----------------
Condylura cristata-Beta-5K	XP_004677673.1	-------------------	--	--------------K----------------
Eptesicus fuscus-Beta-5K	XP_008143183.1	-------------------	-A	-------------------------------
Camelus dromedarius-Beta-5K	XP_010998086.1	-------------------	-A	-------------------------------
Physeter catodon-Beta-5K	XP_023982630.1	---------------A---	--	--------------K----------------
Saimiri boliviensis boliviensi-Beta-5K	XP_010344445.1	--------------P----	--	-------------------------------
Marmota marmota marmota-Beta-5K	XP_015348322.1	-------------------	-A	--------------K----------------
Panthera tigris altaica-Beta-5K	XP_015390909.1	--------------P----	--	--------------K----------------
Equus przewalskii-Beta-5K	XP_008510987.1	-------------------	--	---E----------K----------------
Panthera pardus-Beta-5K	XP_019319198.1	--------------P----	--	--------------K----------------
Felis catus-Beta-5K	XP_019671300.2	--------------P----	--	--------------K----------------
Erinaceus europaeus-Beta-5K	XP_016046043.1	------------------L	--	--------------K----------------
Galeopterus variegatus-Beta-5K	XP_008578886.1	-------------------	--	--------------K----------------
**Birds**				
Haliaeetus albicilla-Beta-5K	KFQ07119.1	-----------------SV	T-	-TTN----------K----------------
Manacus vitellinus-Beta-5K	XP_017936501.1	-----------------SV	T-	-TTN--------N-K----------------
Lonchura striata domestica-Beta-5K	OWK55566.1	-----------------S-	T-	-KTN----------K----------------
Corvus brachyrhynchos-Beta-5K	XP_017586100.1	-----------------SV	T-	-TTN----------K----------------
Numida meleagris-Beta-5K	XP_021236396.1	-----------------SV	V-	-TTN----------K----------------
Cathartes aura-Beta-5K	KFP49800.1	-----------------SV	T-	-TTN----------K----------------
Fulmarus glacialis-Beta-5K	XP_009574388.1	-----------------S-	T-	-TTN----------K----------------
Sturnus vulgaris-Beta-5K	XP_014725312.1	-----------------SV	T-	-TTN----------K----------------
Struthio camelus australis-Beta-5K	XP_009670362.1	-----------------SV	--	-ATN----------K----------------
Gallus gallus-Beta-5K	XP_015135771.1	-----------------SV	V-	-TTN----------K----------------
Anas platyrhynchos-Beta-5K	XP_021123700.1	-----------------SV	V-	-ATN----------K----------------
Chlamydotis macqueenii-Beta-5K	XP_010124567.1	-----------------S-	T-	-TTN----------K----------------
Apaloderma vittatum-Beta-5K	XP_009864634.1	-----------------TV	N-	-ITN----------K----------------

Podiceps cristatus-Beta-5K	KFZ69294.1	-----------------SV	T-	-TTN----------K----------------
Tinamus guttatus-Beta-5K	XP_010224625.1	--------------A--SV	--	-ATN----------K----------------
Anser cygnoides domesticus-Beta-5K	XP_013049494.1	-----------------SV	V-	-ATN----------K----------------
Zonotrichia albicollis-Beta-5K	XP_005491000.1	-----------------SL	T-	-KTN---------------------------
Pelecanus crispus-Beta-5K	KFQ61112.1	-----------------SV	T-	-TTN----------K----------------
Gavia stellata-Beta-5K	XP_009806849.1	-----------------SV	T-	-TTN----------K----------------
Colinus virginianus-Beta-5K	OXB75530.1	-----------------SV	T-	-TAN----------K----------------
Aquila chrysaetos canadensis-Beta-5K	XP_011576030.1	-----------------S-	T-	-TTN----------K----------------
Phaethon lepturus-Beta-5K	KFQ71669.1	-----------------SV	T-	-TTN----------K----------------
Picoides pubescens-Beta-5K	KFV72101.1	-----------------SV	T-	-TTN----------K----------------
Haliaeetus leucocephalus-Beta-5K	XP_010566885.1	-----------------SV	T-	-TTN----------K----------------
Nipponia nippon-Beta-5K	XP_009458847.1	-----------------SV	T-	-TTN----------K----------------
Calidris pugnax-Beta-5K	XP_014807148.1	-----------------SV	T-	-TTN----------K----------------
Taeniopygia guttata-Beta-5K	XP_002191336.1	-----------------S-	T-	-KTN----------K----------------
Aptenodytes forsteri-Beta-5K	XP_009283561.1	-----------------SV	T-	-TTN----------K----------------
Opisthocomus hoazin-Beta-5K	KFR14126.1	-----------------SV	T-	-TTN----------K----------------
Coturnix japonica-Beta-5K	XP_015704584.1	-----------------SV	V-	-TTN----------K---------------
Serinus canaria-Beta-5K	XP_009092185.1	-----------------SV	T-	-KTN----------K----------------
Geospiza fortis-Beta-5K	XP_005420909.1	-----------------SV	T-	-KTN----------K----------------
Mesitornis unicolor-Beta-5K	XP_010183780.1	-----------------SV	T-	ATTN----------K----------------
Callipepla squamata-Beta-5K	OXB62686.1	-----------------SV	T-	-TTN----------K----------------
Merops nubicus-Beta-5K	KFQ22315.1	-----------------SV	S-	-TAN----------K----------------
Falco peregrinus-Beta-5K	XP_005242231.1	-----------------SV	T-	KTTN---------------------------
Pygoscelis adeliae-Beta-5K	XP_009326352.1	-----------------SL	T-	-TTN----------K----------------
Pterocles gutturalis-Beta-5K	XP_010081563.1	-----------S-----LV	T-	-TTN----------K----------------
Chaetura pelagica-Beta-5K	KFU85810.1	-----------------SL	S-	-TTN----------K----------------
Egretta garzetta-Beta-5K	KFP13705.1	--------------T--SV	T-	-TTN----------K----------------
Lepidothrix coronata-Beta-5K	XP_017661575.1	-----------------SV	T-	-TTN--------N-K----------------
Balearica regulorum gibbericep-Beta-5K	KFO12738.1	-----------------SV	P-	-TTN-------R--K----------------
Cariama cristata-Beta-5K	XP_009702170.1	-----------------S-	TP	-TTN----------K----------------
Cuculus canorus-Beta-5K	KFO79437.1	----------------ESV	T-	-TTN----------K----------------
Meleagris gallopavo-Beta-5K	XP_010724018.1	-----------S-----SV	V-	-TTN----------K----------------
Parus major-Beta-5K	XP_015508926.1	-----------------SV	TR	-ATN----------K----------------
Buceros rhinoceros silvestris-Beta-5K	XP_010138959.1	-----------------S-	T-	-TTN---------NK----------------
Pseudopodoces humilis-Beta-5K	XP_005522707.1	-----------------SV	TR	-TTN----------K----------------
**Reptiles**				
Anolis carolinensis-Beta-5K	XP_008101519.1	-----------------CV	--	-STN---------------------------
Gekko japonicus-Beta-5K	XP_015261333.1	--------------T--CV	--	-STN----------K----------------
Pogona vitticeps-Beta-5K	XP_020661323.1	-----------------CV	--	-ST----------------------------
Python bivittatus-Beta-5K	XP_007439496.2	-----------------CV	--	--TN---------Y-----------------
Ophiophagus hannah-Beta-5K	ETE59205.1	-----------------CV	--	--TN---------Y-----------------
Protobothrops mucrosquamatus-Beta-5K	XP_015679138.1	-----------------CV	--	--TN---------Y-----------------
Thamnophis sirtalis-Beta-5K	XP_013907811.1	-----------------CV	--	--TN---------Y-----------------
**Amphibians**				
Rana catesbeiana-Beta-5K	PIO37889.1	-----L--------AV-AV		ISG---------N----RV------------
Nanorana parkeri-Beta-5K	XP_018411161.1	-----L--------AV-TV		ISE---------N-K--QV------------
Xenopus tropicalis-Beta-5K	XP_012827258.1	-----L--------DV-SF		IKES--------N----RM------------
Xenopus laevis-Beta-5K	AAH55973.1	-----L--------DV-FF		IKES--------N-K--RV------------
**Fishes**				
Danio rerio-Beta	XP_005155628.1	-----R-----D--APEPV	AD	ADDE-L------ -KD----I-L--------
Danio rerio-Beta	NP_001004579.1	-----------D--AAEAL		TTD---------T--D--V-I-L--------
Maylandia zebra-Beta	XP_004538536.1	-----L-----NV-DPEPV		ADD--L------ -KD-S--I-L--------
Pundamilia nyererei-Beta-5K	XP_005725614.1	-----L-----NV-DPEPV		ADD--L------ -KD-S--I-L--------
Maylandia zebra-Beta-5K	XP_004538536.1	-----L-----NV-DPEPV		ADD--L------ -KD-S--I-L--------
Oreochromis niloticus-Beta-5K	XP_003440259.2	-----L-----NV-DPEPV		ADD--L------ -KD-S--I-L--------
Neolamprologus brichardi-Beta-5K	XP_006803535.1	-----L-----NV-DPEPV		ADD--L------ -KD-S--I-L--------
Haplochromis burtoni-Beta-5K	XP_005921766.1	-----L-----NV-DPEPV		ADD--L------ -KD-S--I-L--------
Paralichthys olivaceus-Beta-5K	XP_019960688.1	-----L-----NV-DPEPV		ADD--L------ -KD-N--I-L--------
Amphiprion ocellaris-Beta-5K	XP_023138083.1	-----L-----NV-DPEPV		ADD--L------ -KD-S--I-L--------
Kryptolebias marmoratus-Beta-5K	XP_017288436.1	-----L-----NV-DPEPV		ADD--L------ -KD-N--I-L--------
Fundulus heteroclitus-Beta-5K	XP_021179699.1	-----L-----NV-DPEPV		ADD--L------ -KD-N--I-L--------
Xiphophorus maculatus-Beta-5K	XP_023193673.1	-----L-----NL-DPEPV		ADD--L------ -KD-N--I-L--------
Poecilia formosa-Beta-5K	XP_007575909.1	-----L-----NL-DPEPV		ADD--L------ -KD-N--I-L--------
Austrofundulus limnaeus-Beta-5K	XP_013878032.1	-----L-----NV-DP-PV		ADD--L------ --D-S--I-L--------
Acanthochromis polyacanthus-Beta-5K	XP_022072780.1	-----L-----SV-DPEPV		ADD--L------ -KD-S--I-L--------
Lates calcarifer-Beta-5K	XP_018558033.1	-----L-----SV-DPEPV		ADD--F------ -KD-N--I-L--------
Cyprinodon variegatus-Beta-5K	XP_015228657.1	-----L-----TV-DPEPV		ADD--F------ -KD-N--I-L--------
Seriola lalandi dorsalis-Beta-5K	XP_023255674.1	-----L-----SV-DPEPV		ADD--F------ -KD-N--I-L--------
Larimichthys crocea-Beta-5K	XP_010734153.1	-----L-----TV-DPEPV		ADD--F------ -KD-N--I-L--------
Labrus bergylta-Beta-5K	XP_020491677.1	-----L-----NV-DP-PV		ADDE-------R -KD-Q--I-L--------
Oryzias latipes-Beta-5K	XP_011480348.1	-----L-----SV-DPEPV		ADD--F------ --D-N--I-L--------

Monopterus albus-Beta-5K	XP_020470796.1	-----L-----NV-APESV	ADDE-L------ -KD-N--I-L--------
Cynoglossus semilaevis-Beta-5K	XP_016894343.1	-----L-----DVRDPGPV	ADD--L-----R -KD-S--I-L--------
Takifugu rubripes-Beta-5K	XP_011618834.1	-----L-----SM-DPEPL	GDD--F------ YKD-N--I-L--------
Oryzias melastigma-Beta-5K	XP_024154386.1	-----L-----TV-DPEPV	VDD--F------ -KD-N--I-L--------
Stegastes partitus-Beta-5K	XP_008274617.1	-----L---R-NV-DTEPG	TDD--L------ -KD-S--I-L--------
Notothenia coriiceps-Beta-5K	XP_010787650.1	-----L-----NT-ASEPV	ADD--F------ NKD-NF-I-L--------

Mammals

Homo sapiens-Alpha-5K	BAG63614.1	-----------EARR-GTM	-TD-H------RNSK--R---YI--------
Mesocricetus auratus-Alpha-5K	XP_012982621.1	-----------EARR-GTV	-TE-H------RNSK--R---YI--------
Galeopterus variegatus-Alpha-5K	XP_008582482.1	-----------EARR-GTM	-TD-H------RNSK--R---YI--------
Bison bison bison-Alpha-5K	XP_010855825.1	-----------EARR-GTM	-TD-H------RNSK--R---YI--------
Dasypus novemcinctus-Alpha-5K	XP_012378081.2	-----------EARR-GTM	-TD-H------RNNK--R---YI--------
Otolemur garnettii-Alpha-5K	XP_023375185.1	-----------EARR-GTM	-TD-H------RNSK--R---YI--------
Cervus elaphus hippelaphus-Alpha-5K	OWK04510.1	-----------EARR-GTM	-TD-H------RNSK--R---YI--------
Macaca nemestrina-Alpha-5K	XP_011767619.1	-----------EARR-GTM	-TD-H------RNSK--R---YI--------
Chlorocebus sabaeus-Alpha-5K	XP_007975432.1	-----------EARR-GTM	-TD-H------RNSK--R---YI--------
Saimiri boliviensis boliviensi-Alpha-5K	XP_010328091.1	-----------EARR-GTM	-TD-H------RNSK--R---YI--------
Panthera pardus-Alpha-5K	XP_019288329.1	-----------EARR-GTM	-TD-H------RNSK--R---YI--------
Gorilla gorilla gorilla-Alpha-5K	XP_018890710.1	-----------EARR-GTM	-TD-H------RNSK--R---YI--------
Rhinopithecus bieti-Alpha-5K	XP_017733212.1	-----------EARR-GTM	-TD-H------RNSK--R---YI--------
Odocoileus virginianus texanus-Alpha-5K	XP_020730051.1	-----------EARR-GTM	-TD-H------RNSK--R---YI--------
Propithecus coquereli-Alpha-5K	XP_012514222.1	-----------EARR-GTM	-TD-H------RNSK--R---YI--------
Loxodonta africana-Alpha-5K	XP_023403096.1	-----------EARR-GTM	-TD-H------RNSK--R---YI--------
Balaenoptera acutorostrata-Alpha-5K	XP_007178100.1	-----------EARR-GTM	-TD-H------RNSK--R---YI--------
Physeter catodon-Alpha-5K	XP_007130523.1	-----------EARR-GTM	-TD-H------RNSK--R---YI--------
Ovis aries-Alpha-5K	XP_012036418.1	-----------EARR-GTM	-TD-H------RNSK--R---YI--------
Bos taurus-Alpha-5K	XP_005203884.1	-----------EARR-GTM	-TD-H------RNSK--R---YI--------
Pan troglodytes-Alpha-5K	XP_009428656.1	-----------EARR-GTM	-TD-H------RNSK--R---YI--------
Ailuropoda melanoleuca-Alpha-5K	XP_019655316.1	-----------EARR-GTM	-TD-H------RNSK--R---YI--------
Acinonyx jubatus-Alpha-5K	XP_014933058.1	-----------EARR-GTM	-TD-H------RNSK--R---YI--------
Enhydra lutris kenyoni-Alpha-5K	XP_022381744.1	-----------EARR-GTM	-TD-H------RNSK--R---YI--------
Nomascus leucogenys-Alpha-5K	XP_003259270.1	-----------EARR-GTM	-TD-H------RNSK--R---YI--------
Pantholops hodgsonii-Alpha-5K	XP_005970730.1	-----------EARR-GTM	-TD-H------RNSK--R---YI--------
Pan paniscus-Alpha-5K	XP_003817313.1	-----------EARR-GTM	-TD-H------RNSK--R---YI--------
Odobenus rosmarus divergens-Alpha-5K	XP_004404309.1	-----------EARR-GTM	-TD-H------RNSK--R---YI--------
Carlito syrichta-Alpha-5K	XP_021575138.1	-----------EARR-GTM	-TD-H------RNSK--R---YI--------
Colobus angolensis palliatus-Alpha-5K	XP_011793246.1	-----------EARR-GTM	-TD-H------RNSK--R---YI--------
Bos mutus-Alpha-5K	XP_005895003.1	-----------EARR-GTM	-TD-H------RNSK--R---YI--------
Pongo abelii-Alpha-5K	XP_009242802.1	-----------EARR-GTM	-TD-H------RNSK--R---YI--------
Leptonychotes weddellii-Alpha-5K	XP_006728426.1	-----------EARR-GTM	-TD-H------RNSK--R---YI--------
Bubalus bubalis-Alpha-5K	XP_006058227.1	-----------EARR-GTM	-TD-H------RNSK--R---YI--------
Neomonachus schauinslandi-Alpha-5K	XP_021543792.1	-----------EARR-GTM	-TD-H------RNSK--R---YI--------
Ursus maritimus-Alpha-5K	XP_008693896.1	-----------EARR-GTM	-TD-H------RNSK--R---YI--------
Capra hircus-Alpha-5K	XP_005677674.2	-----------EARR-GTM	-TD-H------RNSK--R---YI--------
Rhinopithecus roxellana-Alpha-5K	XP_010385509.1	-----------EARR-GTM	-TD-H------RNSK--R---YI--------
Piliocolobus tephrosceles-Alpha-5K	XP_023069451.1	-----------EARR-GTM	-TD-H------RNSK--R---YI--------
Callithrix jacchus-Alpha-5K	ABY82090.1	-----------EARR-GTM	-TD-H------RNSK--R---YI--------
Mustela putorius furo-Alpha-5K	XP_012903418.1	-----------EARR-GTM	-TD-H------RNSK--R---YI--------
Cebus capucinus imitator-Alpha-5K	XP_017401774.1	-----------EARR-GTM	-TD-H------RNSK--R---YI--------

Birds

Balearica regulorum gibbericep-Alpha-5K	KF015204.1	-----------EARR-GT-	-TD-Q------RNAK--R---YI----V---
Aptenodytes forsteri-Alpha-5K	KFM08822.1	-----------EARR-GT-	-TD-Q------RNAK--R---YI----V---
Eurypyga helias-Alpha-5K	KFW07847.1	-----------EARR-GT-	-TD-Q------RNAK--R---YI----V---
Phaethon lepturus-Alpha-5K	KFQ69620.1	-----------EARR-GT-	-TD-Q------RNAK--R---YI----V---
Acanthisitta chloris-Alpha-5K	KFP88795.1	-----------EARR-GT-	-TD-Q------RNAK--R---YV----V---
Cathartes aura-Alpha-5K	KFP49378.1	-----------EARR-GT-	-TD-Q------RNAK--R---YI----V---
Phoenicopterus ruber ruber-Alpha-5K	KFQ81007.1	-----------EARR-GT-	-TD-Q------RNAK--R---YI----V---
Cariama cristata-Alpha-5K	KFP65666.1	-----------EARR-GT-	-TD-Q------RNAK--R---YI----V---
Picoides pubescens-Alpha-5K	KFV76585.1	-----------EARR-GT-	-TD-Q------RNAK--R---YI----V---
Podiceps cristatus-Alpha-5K	KFZ67268.1	-----------EARR-GT-	-TD-Q------RNAK--R---YI----V---
Gavia stellata-Alpha-5K	KFV53654.1	-----------EARR-GT-	-TD-Q------RNAK--R---YI----V---
Charadrius vociferus-Alpha-5K	KGL86760.1	-----------EARR-GT-	-TD-Q------RNAK--R---YI----V---
Nestor notabilis-Alpha-5K	KFQ47794.1	-----------EARR-GT-	-TD-Q------RNAK--R---YI----V---
Cuculus canorus-Alpha-5K	KF073190.1	-----------EARR-GT-	-TD-Q------RNAK--R---YI----V---
Struthio camelus australis-Alpha-5K	KFV72556.1	-----------EARR-GT-	-TD-Q------RNAK--R---YI----V---
Chlamydotis macqueenii-Alpha-5K	KFP45876.1	-----------EARR-GT-	-TD-Q------RNAK--R---YI----V---
Calypte anna-Alpha-5K	KF096662.1	-----------EARR-GT-	-TD-Q------RNAK--R---YV----V---
Antrostomus carolinensis-Alpha-5K	XP_010163162.1	-----------EARR-GT-	-TD-Q------RNAK--R---YI----V---
Anas platyrhynchos-Alpha-5K	XP_021132788.1	-----------EARR-GT-	-TD-Q------RNAK--R---YI----V---
Melopsittacus undulatus-Alpha-5K	XP_012985591.1	-----------EARR-GT-	-TD-Q------RNAK--R---YI----V---
Anser cygnoides domesticus-Alpha-5K	XP_013056952.1	-----------EARR-GT-	-TD-Q------RNAK--R---YI----V---

Haliaeetus leucocephalus-Alpha-5K	XP_010563784.1	-----------EARR-GT-	-TD-Q------RNAK--R---YI----V---
Aquila chrysaetos canadensis-Alpha-5K	XP_011596815.1	-----------EARR-GT-	-TD-Q------RNAK--R---YI----V---
Callipepla squamata-Alpha-5K	OXB55834.1	-----------EARR-GT-	-TD-Q------RNAK--R---YV----V---
Apteryx australis mantelli-Alpha-5K	XP_013798562.1	-----------EARR-GT-	-TD-Q------RNAK--R---YI----V---
Columba livia-Alpha-5K	XP_021136061.1	-----------EARR-GT-	-TD-Q------RNAK--R---YI----V---
Meleagris gallopavo-Alpha-5K	XP_010722283.1	-----------EARR-GT-	-TD-Q------RNAK--R---YV----V---
Amazona aestiva-Alpha-5K	KQL59296.1	-----------EARR-GT-	-TD-Q------RNAK--R---YI----V---
Nipponia nippon-Alpha-5K	XP_009465378.1	-----------EARR-GT-	-TD-Q------RNAK--R---YI----V---
Gallus gallus-Alpha-5K	NP_001135912.2	-----------EARR-GT-	-TD-Q------RNAK--R---YV----V---
Meleagris gallopavo-Alpha-5K	XP_010722285.1	-----------EARR-GT-	-TD-Q------RNAK--R---YV----V---
Numida meleagris-Alpha-5K	XP_021232499.1	-----------EARR-GT-	-TD-Q------RNAK--R---YV----V---
Coturnix japonica-Alpha-5K	XP_015740163.1	-----------EARR-GT-	-TD-Q------RNAK--R---YV----V---
Chaetura pelagica-Alpha-5K	XP_010001104.1	-----------EARR-GT-	-TD-Q------RNA---R---YV----V---
Colinus virginianus-Alpha-5K	OXB72319.1	-----------EARR-GT-	-TD-HSQQVEQS-P*--R---YV----V---
*KHAVLCPCRMGGIPARNAK			
Mesitornis unicolor-Alpha-5K	KFQ32940.1	-----------EARR-GT-	-TD-QL---H- K--R---YI----V---

Reptiles

Pogona vitticeps-Alpha-5K	XP_020653819.1	-----------EARR-GT-	-TD-Q------RNAK--R---YV----V---
Anolis carolinensis-Alpha-5K	XP_008120464.1	-----------EARR-GT-	-TD-Q-----SRNAK--R--IYV----V---
Python bivittatus-Alpha-5K	XP_015744819.1	-----------EARR-GT-	-TD-Q------RNAK--R---YV----V---
Gekko japonicus-Alpha-5K	XP_015279999.1	-----------EARR-GTV	-TD-Q-----SRNAK--R---YI----V---
Protobothrops mucrosquamatus-Alpha-5K	XP_015680374.1	-----------EARR-GT-	-TD-Q-----SRNAK--R---YV----V---

Amphibian

Xenopus tropicalis-Alpha-5K	OCA14230.1	-----------EARR-GA-	-TD-Q------RNAK--R---YI-V--V---
Xenopus laevis-Alpha-5K	NP_001233235.1	-----------EARR-GA-	-TD-Q------RNAK--R---YI-V--V---
Nanorana parkeri-Alpha-5K	XP_018421916.1	-----------EARR-GP-	-TD-Q------RNTK--R---YI-V------

Fishes

Maylandia zebra-Alpha-5K	XP_004541373.2	--C--------EARGKGAL	DSE-H------RNSK--R--IYI--------
Tetraodon nigroviridis-Alpha-5K	CAG11890.1	--C--------EARGKGAL	DSE-H------RNSK--R---YI--------
Notothenia coriiceps-Alpha-5K	XP_010788253.1	--C--------EARGKGAL	DSE-H-----SRNSK--R--IYI--------
Neolamprologus brichardi-Alpha-5K	XP_006804986.1	--C--------EARGKGAL	DSE-H------RNSK--R--IYI--------
Pundamilia nyererei-Alpha-5K	XP_005732196.1	--C--------EARGKGAL	DSE-H------RNSK--R--IYI--------
Paralichthys olivaceus-Alpha-5K	XP_019948859.1	--C--------EARGKGAL	DSE-H------RNSK--R--IYI--------
Oreochromis niloticus-Alpha-5K	XP_005472748.1	--C--------EARGKGAL	DSE-H------RNSK--R--IYI--------
Boleophthalmus pectinirostris-Alpha-5K	XP_020783608.1	--C--------EARGKGAL	DSE-H------RNSK--R--IYI--------
Acanthochromis polyacanthus-Alpha-5K	XP_022051707.1	--C--------EARGKGAL	DSE-H------RNNK--R--IYI--------
Oncorhynchus mykiss-Alpha-5K	CDQ97059.1	--C--------EARGKGAL	-SE-H------RNSK--R--V-I--------
Austrofundulus limnaeus-Alpha-5K	XP_013885096.1	--C--------EARGKGAL	DSE-H------RNAK--R--IYI--------
Amphiprion ocellaris-Alpha-5K	XP_023117624.1	--C--------EARGKGAL	DSE-H------RNNK--R--IYI--------
Salvelinus alpinus-Alpha-5K	XP_024001797.1	--C--------EARGKGAL	-SE-H------RNSK--R--V-I--------
Hippocampus comes-Alpha-5K	XP_019747686.1	--C--------EARGKGAL	DSE-H------RNGK--R--IYI--------
Stegastes partitus-Alpha-5K	XP_008285964.1	--C--------EARGKGAL	DSE-H------RNT---R--IYI--------
Kryptolebias marmoratus-Alpha-5K	XP_017280565.1	--C--------EARGKGAL	DSE-H------RNAK-DR--IYI--------
Seriola lalandi dorsalis-Alpha-5K	XP_023256608.1	--C--------EARGKGAL	DSE-H-----GRNTK--R--IYI--------
Seriola dumerili-Alpha-5K	XP_022621881.1	--C--------EARGKGAL	DSE-H-----GRNTK--R--IYI--------
Nothobranchius furzeri-Alpha-5K	XP_015806121.1	--C--------EARGKGAL	DSE-HV-----RNAK--R--IYI--------
Oncorhynchus kisutch-Alpha-5K	XP_020358970.1	--C--------EARGKGAL	-SE-H------RNSK--R--V-I--------
Lates calcarifer-Alpha-5K	XP_018541718.1	--C--------EARGKGAL	DSE-H-----GRNAK--R--IYI--------
Esox lucius-Alpha-5K	XP_012994913.1	--C--------EARGKGAL	-SE-H------RNTK--R--V-I--------
Xiphophorus maculatus-Alpha-5K	XP_023186755.1	--C--------EARGKGAL	DSE-HV-----RNVK--R--IYI--------
Fundulus heteroclitus-Alpha-5K	XP_012721150.1	--C--------EARGKGAL	DSE-HV-----RNVK--R--IYI--------
Labrus bergylta-Alpha-5K	XP_020497624.1	--C--------EARGKGAL	DSE-H-----SRNTK--R--IYI--------
Poecilia formosa-Alpha-5K	XP_007556196.1	--C--------EARGKGAL	DSE-HV-----RNVK--R--IYI--------
Poecilia latipinna-Alpha-5K	XP_014875113.1	--C--------EARGKGAL	DSE-HV-----RNVK--R--IYI--------
Poecilia reticulata-Alpha-5K	XP_008429902.1	--C--------EARGKGAL	DSE-HV-----RNVK--R--IYI--------
Poecilia mexicana-Alpha-5K	XP_014839941.1	--C--------EARGKGAL	DSE-HV-----RNVK--R--IYI--------
Oryzias melastigma-Alpha-5K	XP_024123874.1	--C--------EARGKGAM	DSE-H------R-SK--R--IYI--------
Takifugu rubripes-Alpha-5K	XP_011603926.1	--C--------EARGKGAL	DSE-H------RNPK--R---YI--------
Salmo salar-Alpha-5K	XP_014057413.1	--C--------EARGKGAL	-SE-H------RNSK--R--V-I--------
Cynoglossus semilaevis-Alpha-5K	XP_008321275.1	--C--------EARGKGAL	DSENH---M--XNNK--R--IYI--------
Monopterus albus-Alpha-5K	XP_020462945.1	--C--------EAQGKGAL	DSD-H------RNVK--R--IYI--------
Cyprinodon variegatus-Alpha-5K	XP_015248386.1	--C--------EARGKGSL	DSE-HV-----RNAK--R--IYI--------
Oryzias latipes-Alpha-5K	XP_004078182.1	--C--------EARGKEAM	DSE-H------R-SK--R--IYI--------
Clupea harengus-Alpha-5K	XP_012684664.1	--C--------EARGKGAM	DSE-Y------RNS---R--I----------
Astyanax mexicanus-Alpha-5K	XP_022529206.1	-----------EARGKGTL	DSE-QW-----RNSK--R--VYI--------
Mesocricetus auratus-Alpha-5K	XP_012982621.1	-----------EARR-GTV	-TE-H------RNSK--R---YI--------
Pygocentrus nattereri-Alpha-5K	XP_017555944.1	-----------EARGRGTL	DSE-QW-----RNSK--R---YI--------

Mammals

Homo sapiens-Gamma-5K	AAC32904.1	-----------GAAR-EA-	-SD--------VNG---R---HI--------
Otolemur garnettii-Gamma-5K	XP_012659946.2	-----------GAAR-EA-	-SD--------VNG---R---HI--------
Pongo abelii-Gamma-5K	XP_024093427.1	-----------GAAR-EA-	-SD--------VNG---R---HI--------

Pan paniscus-Gamma-5K	XP_008970866.1	-----------GAAR-EA-	-SD--------VNG---R---HI--------
Cebus capucinus imitator-Gamma-5K	XP_017362897.1	-----------GAAR-EA-	-SD--------VNG---R---HI--------
Pan troglodytes-Gamma-5K	XP_016792550.1	-----------GAAR-EA-	-SD--------VNG---R---HI--------
Mesocricetus auratus-Gamma-5K	XP_021080145.1	-----------GAAR-EA-	-TD--------VNG---R---HI--------
Gorilla gorilla gorilla-Gamma-5K	XP_018871823.1	-----------GAAR-EA-	-SD--------VNG---R---HI--------
Microcebus murinus-Gamma-5K	XP_020139527.1	-----------GAAR-EA-	-SD--------VNG---R---HI--------
Callithrix jacchus-Gamma-5K	XP_008985238.1	-----------GAAR-EA-	-SD--------VNG---R---HI--------
Aotus nancymaae-Gamma-5K	XP_021532289.1	-----------GAAR-EA-	-SD--------VNG---R---HI--------
Castor canadensis-Gamma-5K	XP_020036849.1	-----------GAAR-EA-	-TD--------VNG---R---HI--------
Nomascus leucogenys-Gamma-5K	XP_012358835.1	-----------GAAR-EA-	-SD--------VNG---R---HI--------
Galeopterus variegatus-Gamma-5K	XP_008581117.1	-----------GAAR-EA-	-SD--------VNG---R---HI--------
Camelus ferus-Gamma-5K	XP_014410201.1	-----------GAAR-EA-	-TD--------VNG---R---HI--------
Panthera pardus-Gamma-5K	XP_019286317.1	-----------GAAR-EA-	-SD--------VNG---R---HI--------
Felis catus-Gamma-5K	XP_023099540.1	-----------GAAR-EA-	-SD--------VNG---R---HI--------
Leptonychotes weddellii-Gamma-5K	XP_006738797.1	-----------GAAR-EA-	-SD--------VNG---R---HI--------
Sarcophilus harrisii-Gamma-5K	XP_023352567.1	-----------GAAR-ES-	-TD--------VNG---R---HI--------
Loxodonta africana-Gamma-5K	XP_010599556.2	-----------GATR-EA-	-TD--------VNG---R---HI--------
Odobenus rosmarus divergens-Gamma-5K	XP_004395402.1	-----------GAAR-EA-	-SD--------VNG---R---HI--------
Enhydra lutris kenyoni-Gamma-5K	XP_022381192.1	-----------GAAR-EA-	-SD--------VNG---R---HI--------
Propithecus coquereli-Gamma-5K	XP_012501604.1	-----------GAAR-EA-	-SD--------VNG---R---HI--------
Rhinopithecus roxellana-Gamma-5K	XP_010365585.1	-----------GAAR-EP-	-SD--------VNG---R---HI--------
Macaca fascicularis-Gamma-5K	XP_015295799.1	-----------GAAR-EP-	-SD--------VNG---R---HI--------
Macaca mulatta-Gamma-5K	EHH29471.1	-----------GAAR-EP-	-SD--------VNG---R---HI--------
Orycteropus afer afer-Gamma-5K	XP_007949279.1	-----------GAAR-EA-	-TD--------VNG---R---HI--------
Piliocolobus tephrosceles-Gamma-5K	XP_023039373.1	-----------GAAR-EP-	-SD--------VNG---R---HI--------
Saimiri boliviensis boliviensi-Gamma-5K	XP_010347757.1	-----------GAAR-EA-	-SD--------VNG---R---HI--------
Neomonachus schauinslandi-Gamma-5K	XP_021561194.1	-----------GAAR-EA-	-SD--------VNG---R---HI--------
Papio anubis-Gamma-5K	XP_003914709.1	-----------GAAR-EP-	-SD--------VNG---R---HI--------
Cercocebus atys-Gamma-5K	XP_011928451.1	-----------GAAR-EP-	-SD--------VNG---R---HI--------

Birds

Geospiza fortis-Gamma-5K	XP_014165085.1	-----------GAAR-EA-	DTD--------VNGK--R---HV--------
Apaloderma vittatum-Gamma-5K	KFP77981.1	-----------GAAR-ES-	DTD--------VNGK--R---HV--------
Gavia stellata-Gamma-5K	KFV49688.1	-----------GAAR-ES-	DTD--------VNGK--R---HV--------
Colius striatus-Gamma-5K	KFP30926.1	-----------GAAR-ES-	DTD--------VNGK--R---HV--------
Balearica regulorum gibbericep-Gamma-5K	KFO06958.1	-----------GAAR-ES-	DTD--------VNGK--R---HV--------
Podiceps cristatus-Gamma-5K	KFZ64041.1	-----------GAAR-ES-	DTD--------VNGK--R---HV--------
Phalacrocorax carbo-Gamma-5K	KFW78608.1	-----------GAAR-ES-	DTD--------VNGK--R---HV--------
Chaetura pelagica-Gamma-5K	KFU93981.1	-----------GAAR-ES-	DTD--------VNGK--R---HV--------
Aptenodytes forsteri-Gamma-5K	KFM06020.1	-----------GAAR-ES-	DTD--------VNGK--R---HV--------
Cyanistes caeruleus-Gamma-5K	XP_023798643.1	-----------GAAR-ES-	DTD--------VNGK--R---HV--------
Corvus brachyrhynchos-Gamma-5K	KFO53565.1	-----------GAAR-ES-	DTD--------VNGK--R---HV--------
Haliaeetus albicilla-Gamma-5K	XP_009926191.1	-----------GAAR-ES-	DTD--------VNGK--R---HV--------
Eurypyga helias-Gamma-5K	XP_010151218.1	-----------GAAR-ES-	DTD--------VNGK--R---HV--------
Manacus vitellinus-Gamma-5K	XP_017939015.1	-----------GAAR-EA-	DTD--------VNGK--R---HV--------
Cuculus canorus-Gamma-5K	KFO78532.1	-----------GAAR-ES-	DTD--------VNGK--R---HV--------
Mesitornis unicolor-Gamma-5K	KFQ21874.1	-----------GATR-ES-	DTD--------VNGK--R---HV--------
Lonchura striata domestica-Gamma-5K	XP_021383894.1	-----------GAAR-EA-	DTD--------VNGK--R---HV--------
Acanthisitta chloris-Gamma-5K	XP_009079275.1	-----------GAAR-ES-	DTD--------VNGK--R---HV--------
Calypte anna-Gamma-5K	XP_008496647.1	-----------GAAR-ES-	DTD--------VNGK--R---HV--------
Opisthocomus hoazin-Gamma-5K	XP_009940139.1	-----------GAAR-ES-	DTD--------VNGK--R---HV--------
Patagioenas fasciata monilis-Gamma-5K	OPJ77564.1	-----------GAAR-ES-	DTD--------VNGK--R---HV--------
Leptosomus discolor-Gamma-5K	XP_009951577.1	-----------GAAR-ES-	DTD--------VNGK--R---HV--------
Antrostomus carolinensis-Gamma-5K	XP_010166918.1	-----------GAAR-ES-	DTD--------VNGK--R---HV--------
Aquila chrysaetos canadensis-Gamma-5K	XP_011591930.1	-----------GAAR-ES-	DTD--------VNGK--R---HV--------
Anas platyrhynchos-Gamma-5K	EOB01100.1	-----------GAAR-ES-	DTD--------VNGK--R---HV--------
Columba livia-Gamma-5K	XP_021137288.1	-----------GAAR-ES-	DTD--------VNGK--R---HV--------
Pygoscelis adeliae-Gamma-5K	XP_009321283.1	-----------GAAR-ES-	DTD--------VNGK--R---HV--------
Serinus canaria-Gamma-5K	XP_018777310.1	-----------GAAR-EA-	DTD--------VNGK--R---HV--------
Falco cherrug-Gamma-5K	XP_014143069.1	-----------GAAR-ES-	DTD--------VNGK--R---HV--------
Chlamydotis macqueenii-Gamma-5K	XP_010124972.1	-----------GAAR-EP-	DTD--------VNGK--R---HV--------
Lepidothrix coronata-Gamma-5K	XP_017692513.1	-----------GAAR-EA-	DTD--------VNGK--R---HV--------
Parus major-Gamma-5K	XP_015507018.1	-----------GAAR-ES-	DTD--------VNGK--R---HV--------
Apteryx australis mantelli-Gamma-5K	XP_013798587.1	-----------GAAR-ES-	DTD--------VNGK--R---HV--------
Pseudopodoces humilis-Gamma-5K	XP_014115569.1	-----------GAAR-ES-	DTD--------VNGK--R---HV--------
Gallus gallus-Gamma-5K	NP_001305950.1	-----------GAAR-ES-	DTD--------VNGK--R---HV--------
Taeniopygia guttata-Gamma-5K	XP_012426629.1	-----------GAAR-ES-	DTD--------VNGK--R---HV--------
Sturnus vulgaris-Gamma-5K	XP_014739865.1	-----------GAAR-ES-	DTD--------VNGK--R---HV--------
Ficedula albicollis-Gamma-5K	XP_016159970.1	-----------GAAR-ES-	DTD--------VNGK--R---HV--------
Amazona aestiva-Gamma-5K	KQK82211.1	-----------GAVR-EP-	DTD-A------VNGK--R---HV--------
Numida meleagris-Gamma-5K	XP_021234277.1	-----------GAAR-ES-	DTD--------VNGK--R---HV--------
Melopsittacus undulatus-Gamma-5K	XP_005140981.2	-----------GAVR-ES-	DTD-A------VNGK--R---HV--------

**Reptiles**			
Gekko japonicus-Gamma-5K	XP_015276073.1	-----------GAAR-ES-	-TD--------VNGK--R---HV--------
Anolis carolinensis-Gamma-5K	XP_008123366.1	-----------GAAR-ES-	DTD--------VNGK--R---HV--------
Python bivittatus-Gamma-5K	XP_007441380.1	-----------GAAR-ES-	DTD--------VNGK--R---HV--------
Pogona vitticeps-Gamma-5K	XP_020636870.1	-----------GAAR-ES-	DTD--------VNGK--R---HV--------
Thamnophis sirtalis-Gamma-5K	XP_013927381.1	-----------GAAR-EP-	DTD--------VNGK--R---HV--------
**Amphibianss**			
Protobothrops mucrosquamatus-Gamma-5K	XP_015666168.1	-----------GAAR-EP-	DTD--------VNG---R---HV--------
Nanorana parkeri-Gamma-5K	XP_018420852.1	-----------GAAH-ES-	DTD--------VNG---R---YI--------
Xenopus tropicalis-Gamma-5K	OCA49041.1	-----------GAAH-ES-	DTD--------VNGK--R---YI--------
Xenopus laevis-Gamma-5K	XP_018109526.1	-----------GAAH-ES-	DTD--------VNGK--R---YI--------
**Fishes**			
Oreochromis niloticus-Gamma-5K	XP_005476184.1	---S-------GSTCR-TL	-QD--------MGAK--R----I--------
Maylandia zebra-Gamma-5K	XP_012772289.1	---S-------GSTCR-TL	-QD--------MGAK--R----I--------
Haplochromis burtoni-Gamma-5K	XP_005920620.1	---S-------GSTCR-TL	-QD--------MGAK--R----I--------
Neolamprologus brichardi-Gamma-5K	XP_006786750.1	---S-------GSTCR-TL	-QD--------MGAK--R----I--------
Pundamilia nyererei-Gamma-5K	XP_005721179.1	---S-------GSTCR-TL	-QD--------MGAK--R----I--------
Austrofundulus limnaeus-Gamma-5K	XP_013876251.1	-----------GSTCR-TL	DQD--------M-SK--R----I--------
Poecilia reticulata-Gamma-5K	XP_017165352.1	-----------GSTCR-TL	-HD--------MGSK--R----V--------
Poecilia formosa-Gamma-5K	XP_007571215.1	-----------GSTCR-AL	-HD--------MGSK--R----I--------
Poecilia latipinna-Gamma-5K	XP_014911115.1	-----------GSTCR-AL	-HD--------MGSK--R----I--------
Xiphophorus maculatus-Gamma-5K	XP_005797142.2	-----------GSTCR-AM	-HD--------MGSK--R----I--------
Fundulus heteroclitus-Gamma-5K	XP_012729576.1	-----I-----GSTCR-TL	-QD--------VGSK--R----I--------
Acanthochromis polyacanthus-Gamma-5K	XP_022046423.1	--T--------GSTCR-TL	DHD--------VGSK--R----I--------
Amphiprion ocellaris-Gamma-5K	XP_023121344.1	--T--------GSTCR-TL	DHD--------VGSK--R----I--------
Stegastes partitus-Gamma-5K	XP_008304116.1	--T--------GSTCR-TL	DHD--------VGSK--R----I--------
Poecilia mexicana-Gamma-5K	XP_014856633.1	-----------GSTCR-AL	AHD--------MGSK--R----I--------
Lates calcarifer-Gamma-5K	XP_018532494.1	-----------GSTCR-TL	DHD--------VGGK--R----I--------
Seriola dumerili-Gamma-5K	XP_022610606.1	-----------GSTCR-TL	DHD--------VGGK--R----I--------
Seriola lalandi dorsalis-Gamma-5K	XP_023267354.1	-----------GSTCR-TL	DHD--------VGGK--R----I--------
Cyprinodon variegatus-Gamma-5K	XP_015241514.1	-----I-----GTTCRETL	-HD--------MGSK--R----I--------
Oryzias melastigma-Gamma-5K	XP_024129977.1	F-T--------GSTCR-TL	DHD--------V-SK--R----I--------
Paralichthys olivaceus-Gamma-5K	XP_019956130.1	-----------GSTCR-TL	DHD--------V-GK--R----I--------
Nothobranchius furzeri-Gamma-5K	XP_015812680.1	-----------GSTCR-TL	DHE--------A-GK--R----I--------
Kryptolebias marmoratus-Gamma-5K	XP_017277796.1	-----------GSTCR-TL	DHD--------V-SK--C----V--------
Oryzias latipes-Gamma-5K	XP_004079345.2	F-T--------GSTCR-TL	DHD--------V-SK--H----I--------
Cynoglossus semilaevis-Gamma-5K	XP_016897956.1	-----------GSACR-AL	DHD--------V-SK-DR----I--------
Notothenia coriiceps-Gamma-5K	XP_010784186.1	-----------GSTCR-TL	DHD--------VGSK--R----I--------

Figure S7. Detailed species distribution information for the 1-4 aa conserved deletions in PIP4Kγ isoforms shown in Figure 7.

Mammals PIP4Kγ

		113		157
Homo sapiens-Gamma-4K	NP_079055.3	DRFGIDDQDYLVSLTRNPPSESEG		SDGRFLISYDRTLVIKEVSSE
Colobus angolensis palliatus-Gamma-4K	XP_011811608.1	------------------------		---------------------
Pan troglodytes-Gamma-4K	PNI39221.1	------------------------		---------------------
Heterocephalus glaber-Gamma-4K	EHA99894.1	----------------S-------		---------------------
Pongo abelii-Gamma-4K	PNJ44552.1	------------------------		-------C-------------
Leptonychotes weddellii-Gamma-4K	XP_006740137.1	----------------S-------		---------------------
Papio anubis-Gamma-4K	XP_021778219.1	------------------------		---------------------
Jaculus jaculus-Gamma-4K	XP_012805360.1	----------------S-------		---------------------
Macaca mulatta-Gamma-4K	XP_015007545.1	------------------------		---------------------
Carlito syrichta-Gamma-4K	XP_008059033.1	------------------------		---------------------
Nomascus leucogenys-Gamma-4K	XP_012365624.1	------------------------		---------------------
Mandrillus leucophaeus-Gamma-4K	XP_011843974.1	------------------------		---------------------
Chlorocebus sabaeus-Gamma-4K	XP_008002017.1	------------------------		---------------------
Rhinopithecus roxellana-Gamma-4K	XP_010387252.1	------------------------		---------------------
Gorilla gorilla gorilla-Gamma-4K	XP_004053495.1	------------------------		---------------------
Saimiri boliviensis boliviensi-Gamma-4K	XP_010335544.1	--------------------K---		---------------------
Aotus nancymaae-Gamma-4K	XP_012325190.1	--------------------K---		---------------------
Myotis davidii-Gamma-4K	ELK35081.1	----------------S-------		---------------------
Tupaia chinensis-Gamma-4K	ELV09232.1	----------------S-------		---------------------
Pteropus vampyrus-Gamma-4K	XP_023380548.1	----------------S-------		---------------------
Echinops telfairi-Gamma-4K	XP_004700570.1	----------------S-------		---------------------
Microtus ochrogaster-Gamma-4K	XP_005358035.1	----------------S-------		---------------------
Octodon degus-Gamma-4K	XP_023563071.1	----------------S-------		---------------------
Cebus capucinus imitator-Gamma-4K	XP_017391593.1	--------------------K---		---------------------
Rhinolophus sinicus-Gamma-4K	XP_019597823.1	----------------S-------		---------------------
Monodelphis domestica-Gamma-4K	XP_016282376.1	----------Q-----S-------		------V--------------
Meriones unguiculatus-Gamma-4K	XP_021513464.1	----------------S-------		---------------------
Loxodonta africana-Gamma-4K	XP_010596604.1	----------------S-------		---------------------
Cavia porcellus-Gamma-4K	XP_013014825.1	----------------S-------		---------------------
Orcinus orca-Gamma-4K	XP_004276596.1	----------------S-------		---------------------
Chrysochloris asiatica-Gamma-4K	XP_006859545.1	----------------S-------		---------------------
Physeter catodon-Gamma-4K	XP_007103960.1	----------------S-------		---------------------
Canis lupus familiaris-Gamma-4K	XP_022279807.1	----------------S-------		---------------------
Ictidomys tridecemlineatus-Gamma-4K	XP_021589204.1	----------------S-------		---------------------
Chinchilla lanigera-Gamma-4K	XP_005397457.1	----------------S-------		---------------------
Fukomys damarensis-Gamma-4K	XP_010642889.1	----------------S-------		---------------------
Peromyscus maniculatus bairdii-Gamma-4K	XP_006973365.1	----------------S-------		---------------------
Panthera pardus-Gamma-4K	XP_019310451.1	----------------S-------		---------------------
Miniopterus natalensis-Gamma-4K	XP_016080057.1	----------------S-------		---------------------
Eptesicus fuscus-Gamma-4K	XP_008146924.1	----------------S-------		---------------------
Trichechus manatus latirostris-Gamma-4K	XP_012412018.1	----------------S-------		---------------------
Nannospalax galili-Gamma-4K	XP_008842285.1	----------------S-------		---------------------
Enhydra lutris kenyoni-Gamma-4K	XP_022378705.1	----------------S-------		---------------------
Felis catus-Gamma-4K	XP_003988999.1	----------------S-------		---------------------
Myotis lucifugus-Gamma-4K	XP_006093625.1	----------------S-------		---------------------
Orycteropus afer afer-Gamma-4K	XP_007948228.1	----------------S-------		---------------------
Pteropus alecto-Gamma-4K	XP_006909018.1	----------------S-------		---------------------
Ursus maritimus-Gamma-4K	XP_008682580.1	----------------S-------		---------------------
Mustela putorius furo-Gamma-4K	XP_004773296.1	----------------S-------		---------------------
Odobenus rosmarus divergens-Gamma-4K	XP_004401791.1	----------------S-------		---------------------
Galeopterus variegatus-Gamma-4K	XP_008575628.1	----------------S-------		---------------------
Panthera tigris altaica-Gamma-4K	XP_007075077.1	----------------S-------		---------------------

Birds PIP4Kγ

Apteryx australis mantelli-Gamma-4K	XP_013808128.1	E---------Q-----S--HSEG		--R---L-------V--L---
Colinus virginianus-Gamma-4K	OXB50802.1	E---------Q-----S--HAED		G-R-L-L-------V--L---
Nipponia nippon-Gamma-4K	XP_009472221.1	E---------Q-----S--HWEG		--R---L-S-----V--L---
Aquila chrysaetos canadensis-Gamma-4K	XP_011599681.1	E---------Q-----S--HWEG		--R---L-S-----V--L---
Haliaeetus leucocephalus-Gamma-4K	XP_010571143.1	E---------Q-----S--HWEG		--R---L-S-----V--L---
Numida meleagris-Gamma-4K	XP_021237437.1	E---------Q-----S--HAED		G-R-L-L-------V--L---
Patagioenas fasciata monilis-Gamma-4K	OPJ67307.1	E---------Q-----S--HWEG		--R---L-S-----A--L---
Gallus gallus-Gamma-4K	XP_015129207.1	E---------Q-----S--HAED		G-R-L-L-------V--L---
Calypte anna-Gamma-4K	XP_008496595.1	E--------FQ---S-P--HWEG		-GR---L-S--S--L--L---
Parus major-Gamma-4K	XP_015472211.1	E---------Q-----S--RWAG		-GH-L-L-A-----L--L---
Pseudopodoces humilis-Gamma-4K	XP_005532053.1	E---------Q-----S--RWAG		-GH-L-L-A-----L--L---
Sturnus vulgaris-Gamma-4K	XP_014747327.1	E---V-----Q-----S--RWAG		-GH-L-L-A-----L--L---
Lonchura striata domestica-Gamma-4K	OWK49884.1	E---V-----Q---A-S--RWAG		-GR-L-L-A-----L--L---
Calidris pugnax-Gamma-4K	XP_014814995.1	LAVLAWGVNHQ-----S--HWDG		--R-L-L-------V--L---

Reptiles PIP4Kγ

Thamnophis sirtalis-Gamma-4K	XP_013921070.1	E--N------Q-----S--TYETE		GG----L-----V----IT--
Python bivittatus-Gamma-4K	XP_007422116.1	E--N------Q-----S--TYETE		GG----L-----V----IT--
Anolis carolinensis-Gamma-4K	XP_003223671.1	E--S------Q-----S--TYETE		GG----L----------I---
Pogona vitticeps-Gamma-4K	XP_020634130.1	E--S------------S--TYETE		GG----L----------I---
Gekko japonicus-Gamma-4K	XP_015277147.1	E--N------Q-----S--TYETE	G	GG----M---K-----LI---
Xenopus tropicalis-Gamma-4K	XP_017946647.1	E--------FQA----SS-YCESE	G	H-----L---K------I---

Amphibians PIP4Kγ

Xenopus laevis-Gamma-4K	NP_001088764.1	E--------FQ-----SS-YCESE	G	H-----L---K------I---
Xenopus tropicalis-Gamma-4K	OCA46528.1	E--------FQA----SS-YCESE	G	H-----L---K------I---
Nanorana parkeri-Gamma-4K	XP_018424949.1	E---------Q-----SS-YSESE	A	H-----L---K--AV--I---

Fishes PIP4Kγ

Danio rerio-Gamma-4K	NP_956395.2	E----E-L--QA--A-SA-MKGD-	Q	GE-LLFT------IV-QI---
Maylandia zebra-Gamma-4K	XP_004560306.1	E----E-L--Q-----S--FSVDD	Q	GE-LL-N-------V-QI---
Cyprinus carpio-Gamma-4K	XP_018928439.1	E----E-L--Q---A-SA-MRGD-	Q	GE-LLFT------IV-QI---
Sinocyclocheilus anshuiensis-Gamma-4K	XP_016354281.1	E----E-L--Q---A-SA-MRGD-	Q	GE-LLFT------IV-QI---
Sinocyclocheilus grahami-Gamma-4K	XP_016145970.1	E----E-L--Q---A-SA-MRGD-	Q	GE-LLFT------IV-QI---
Sinocyclocheilus anshuiensis-Gamma-4K	XP_016310233.1	E----E-L--Q---A-SA-MRGD-	Q	GE-LLFT------IA-QI---
Sinocyclocheilus grahami-Gamma-4K	XP_016139347.1	E----E-L--Q---A-SA-MRGD-	Q	GE-LLFT------IA-QI---
Sinocyclocheilus rhinocerous-Gamma-4K	XP_016376478.1	E----E-L--Q---A-SA-MRGD-	Q	GE-LLFT------IA-QI---
Pygocentrus nattereri-Gamma-4K	XP_017563406.1	E----E-L--Q-----S--VKG--	Q	GE- LFT---------QI---
Danio rerio-Gamma-4K	XP_003199456.1	E---VEH---Q-----S--VKDDK	EEE	AVLLL-K------IV-QI---
Pundamilia nyererei-Gamma-4K	XP_005739600.1	E----E---FQ---A-SS-LRHDE	GK	YA-LL-T----------I---
Maylandia zebra-Gamma-4K	XP_004545042.1	E----E---FQ---A-S--LRHDE	GK	YA-LL-T----------I---
Seriola lalandi dorsalis-Gamma-4K	XP_023287472.1	E----E-L--Q-----S--LR-GD	DQ	GE-LL-N---------QI---
Seriola dumerili-Gamma-4K	XP_022618948.1	E----E-L--Q-----S--LR-GD	DQ	GE-LL-N---------QI---
Larimichthys crocea-Gamma-4K	XP_010740921.2	E----E-L--Q-----S--MR-VD	DQ	GE-LL-N---------QI---
Amphiprion ocellaris-Gamma-4K	XP_023117013.1	E----E-L--Q-----SS-MR-VD	DQ	GE-LL-N----A--V-QI---
Kryptolebias marmoratus-Gamma-4K	XP_017291956.1	E----E-L--Q-----S--MR-AD	NQ	GEVLL-T---------QI---
Takifugu rubripes-Gamma-4K	XP_003976150.1	E----E-L--QI----S--TR-AD	DQ	GE-FL-N---------QI---

Mammals PIP4Kα

Homo sapiens-Alpha-4K	NP_005019.2	E--------FQN----SA-LPNDS	QAR	-GA--HT---KRYI--TIT--
Castor canadensis-Alpha-4K	XP_020023048.1	E--------FQN----SA-LPNDS	---	-GA--HT---KRYI--TIT--
Rattus norvegicus-Alpha-4K	XP_006254387.1	E--------FQN----SA-LPNDS	---	-GA--HT---KRY---TIT--
Rhinopithecus bieti-Alpha-4K	XP_017722939.1	E--------FQN----SA-LPNDS	---	-GA--HT---KRYI--TIT--
Pantholops hodgsonii-Alpha-4K	XP_005975971.1	E--------FQN----SA-LPNDS	---	-GA--HT---KRYI--TIT--
Panthera pardus-Alpha-4K	XP_019316107.1	E--------FQN----SA-LPNDS	---	-GA--HT---KRYI--TIT--
Phascolarctos cinereus-Alpha-4K	XP_020822528.1	E--------FQN----SA-LPNDS	---	-GA--HT---KRYI--TIT--
Hipposideros armiger-Alpha-4K	XP_019488697.1	E--------FQN----SA-LPNDS	---	-GA--HT---KRY---TIT--
Miniopterus natalensis-Alpha-4K	XP_016080208.1	E--------FQN----SA-LPNDS	---	-GA--HT---KRYI--TIT--
Mandrillus leucophaeus-Alpha-4K	XP_011846714.1	E--------FQN----SA-LPNDS	---	-GA--HT---KRYI--TIT--
Camelus ferus-Alpha-4K	XP_006193040.1	E--------FQN----SA-LPNDS	---	-GA--HT---KRYI--TIT--
Pteropus vampyrus-Alpha-4K	XP_011362713.1	E--------FQN----SA-LPNDS	---	-GA--HT---KRY---TIT--
Myotis brandtii-Alpha-4K	XP_014394848.1	E--------FQN----SA-LPNDS	---	-GA--HT---KRYI--TIT--
Marmota marmota marmota-Alpha-4K	XP_015350772.1	E--------FQN----SA-LPNDS	---	-GA--HT---KRY---TIT--
Fukomys damarensis-Alpha-4K	XP_019062405.1	E--------FQN----SA-LPNDS	---	-GA--HT---KRY---TIT--
Cricetulus griseus-Alpha-4K	XP_016829655.1	E--------FQN----SA-LPNDS	---	-GA--HT---KRY---TIT--
Papio anubis-Alpha-4K	XP_021798805.1	E--------FQN----SA-LPNDS	---	-GA--HT---KRYI--TIT--
Rhinolophus sinicus-Alpha-4K	XP_019596557.1	E--------FQN----SA-LPNDS	---	-GA--HT---KRY---TIT--
Galeopterus variegatus-Alpha-4K	XP_008569555.1	E--------FQN----SA-LPNDS	---	-GA--HT---KRY---TIT--
Trichechus manatus latirostris-Alpha-4K	XP_023590804.1	E--------FQN----SA-LPNDS	---	-GA--HT---KRY---TIT--
Meriones unguiculatus-Alpha-4K	XP_021507920.1	E--------FQN----SA-LPNDS	---	-GA--HT----RY---TIT--
Ornithorhynchus anatinus-Alpha-4K	XP_007671345.1	E--------FQN----SA-LPNDS	---	-GA--H----KRYI--TIT--
Tursiops truncatus-Alpha-4K	XP_019788953.1	E--------FQN----SA-LPNDS	---	-GA--HT---KRYI--TIT--
Chrysochloris asiatica-Alpha-4K	XP_006869963.1	E--------FQN----SA-LPNDS	---	-GA--HT----RY---TIT--
Enhydra lutris kenyoni-Alpha-4K	XP_022371404.1	E--------FQN----SA-LPNDS	---	-GA--HT---KRYI--TIT--
Erinaceus europaeus-Alpha-4K	XP_016045188.1	E--------FQN----SA-LPNDS	---	-GA--HT---KRYI--TIT--
Chlorocebus sabaeus-Alpha-4K	XP_008000685.1	E--------FQN----SA-LPNDS	---	-GA--HT---KRYI--TIT--
Delphinapterus leucas-Alpha-4K	XP_022436191.1	E--------FQN----SA-LPNDS	---	-GA--HT---KRYI--TIT--
Mustela putorius-Alpha-4K	XP_012914369.1	E--------FQN----SA-LPNDS	---	-GA--HT---KRYI--TIT--
Propithecus coquereli-Alpha-4K	XP_012499889.1	E--------FQN----SA-LPNDS	---	-GA--HT---KRYI--TIT--
Bubalus bubalis-Alpha-4K	XP_006080013.1	E--------FQN----SA-LPNDS	---	-GA--HT---KRYI--TIT--
Ailuropoda melanoleuca-Alpha-4K	XP_002913198.1	E--------FQN----SA-LPNDS	---	-GA--HT---KRYI--TIT--
Octodon degus-Alpha-4K	XP_023560737.1	E--------FQN----SA-LPNDS	---	-GA--HT---KRY---TITG-
Nomascus leucogenys-Alpha-4K	XP_012354754.1	E--------FQN----SA-LPNDS	---	-GA--HT---KRYI--TIT--
Dipodomys ordii-Alpha-4K	XP_012870167.1	E--------FQN----SA-LPNDS	---	-GA--HT---KRYI--TIT--
Mus musculus-Alpha-4K	NP_001342075.1	E--------FQN----SA-LPNDS	---	-GA--HT---KRY---TIT--
Ovis aries-Alpha-4K	XP_004014270.1	E--------FQN----SA-LPNDS	---	-GA--HT---KRYI--TIT--
Rousettus aegyptiacus-Alpha-4K	XP_015980169.1	E--------FQN----SA-LPNDS	---	-GA--HT---KRY---TIT--
Elephantulus edwardii-Alpha-4K	XP_006887123.1	E--------FQN----SA-LP-DS	---	-GA--HT---KRY---TIT--
Otolemur garnettii-Alpha-4K	XP_003786808.1	E--------FQN----SA-LPNDS	---	-GA--HT---KRYI--TIT--
Bos taurus-Alpha-4K	NP_001179698.1	E--------FQN----SA-LPNDS	---	-GA--HT---KRYI--TIT--
Sarcophilus harrisii-Alpha-4K	XP_023360857.1	E--------FQN----SA-LPNDS	---	-GA--HT---KRYI--TIT--
Orcinus orca-Alpha-4K	XP_004280994.1	E--------FQN----SA-LPNDS	---	-GA--HT---KRYI--TIT--

Neomonachus schauinslandi-Alpha-4K	XP_021552362.1	E--------FQN----SA-LPNDS --- -GA--HT---KRYI--TIT--
Felis catus-Alpha-4K	XP_003988198.1	E--------FQN----SA-LPNDS --- -GA--HT---KRYI--TIT--
Ochotona princeps-Alpha-4K	XP_004578610.1	E--------FQN----SA-LPNDS --- -GA--HT---KRY---TIT--
Microcebus murinus-Alpha-4K	XP_012622763.1	E--------FQN----SA-LPNDS --- -GA--HT---KRY---TIT--
Monodelphis domestica-Alpha-4K	XP_007504939.1	E--------FQN----SA-LPNDS --- -GA--HT---KRY---TIT--
Ceratotherium simum simum-Alpha-4K	XP_004425357.1	E--------FQN----SA-LPNDS --- -GA--HT---KRYI--TIT--
Heterocephalus glaber-Alpha-4K	XP_004859979.1	E--------FQN----SA-LPNDS --- -GA--HT---KRY---TIT--
Eptesicus fuscus-Alpha-4K	XP_008147081.1	E--------FQN----SA-LPNDS --- -GA--HT---KRYI--TIT--
Callithrix jacchus-Alpha-4K	XP_002750139.1	E--------FQN----SA-LPNDS --- -GA--HT---KRY---TIT--
Tupaia chinensis-Alpha-4K	XP_006139771.1	E--------FQN----SA-LPNDS --- -GA--HT---KRY---TIT--
Saimiri boliviensis boliviensi-Alpha-4K	XP_003930713.1	E--------FQN----SA-LPNDS --- -GA--HT---KRY---TIT--
Fukomys damarensis-Alpha-4K	XP_019062406.1	E--------FQN----SA-LPNDS --- -GA--HT---KRY---TIT--
Nannospalax galili-Alpha-4K	XP_008829944.1	E--------FQN----SA-LPNDS --- -GA--HT---KRY---TIT--
Condylura cristata-Alpha-4K	XP_004691357.1	E--------FQN----SA-LPNDS --- -GA--HT---KRY---TIT--
Oryctolagus cuniculus-Alpha-4K	XP_002717443.2	E--------FQN----SA-LPNDS --- -GA--HT---KRY---TIT--
Ictidomys tridecemlineatus-Alpha-4K	XP_005325824.1	E--------FQN----SA-LPNDS --- -GA--HT---KRY---TIT--
Sorex araneus-Alpha-4K	XP_004605412.1	E--------FQN----SA-LPNDS --- -GA--HT---KRYI--TIT--
Mesocricetus auratus-Alpha-4K	XP_021090108.1	E--------FQN----SA-LPNDS --- -GA--HT---KRY---TIT--
Peromyscus maniculatus bairdii-Alpha-4K	XP_006978789.1	E--------FQN----SA-LPNDS --- -GA--HT---KRY---TIT--
Piliocolobus tephrosceles-Alpha-4K	XP_023079035.1	E--------FQN----SA-LPNDS --- -GA--HT---KRYI--TIT--
Galeopterus variegatus-Alpha-4K	XP_008569556.1	E--------FQN----SA-LPNDS --- -GA--HT---KRY---TIT--
Microtus ochrogaster-Alpha-4K	XP_005354724.1	E--------FQN----SA-LPNDS --- -GA--HT---KRY---TIT--
Carlito syrichta-Alpha-4K	XP_008061830.1	E--------FQN----SA-LPNDS --- -GA--HT---KRY---TIT--
Sus scrofa-Alpha-4K	NP_001182710.1	E--------FQN----SA-LPNDS --- -GA--HT----RY---TIT--
Jaculus jaculus-Alpha-4K	XP_004668473.1	E--------FQN----SA-LPNDS --- -GA--HS---KRYI--TIT--
Cebus capucinus imitator-Alpha-4K	XP_017391066.1	E--------FQN----SA-LPNDS --- -GA--HT---KRY---TIT--
Balaenoptera acutorostrata-Alpha-4K	XP_007189941.1	E--------FQN----SA-LPNDS --- -GA--HM---KRYI--TIT--
Orycteropus afer-Alpha-4K	XP_007934555.1	E--------FQN----SA-LPNDS --- -GA--HT---KRY---AIT--
Equus przewalskii-Alpha-4K	XP_008508780.1	E--------FQN----SA-LPNDS --- -GA--HT-C-KRY---TIT--

Birds PIP4Kα

Merops nubicus-Alpha-4K	XP_008943768.1	E--------FQN----SA-LANDS --- -GA--HT---KRYI--TIT--
Chlamydotis macqueenii-Alpha-4K	XP_010120727.1	E--------FQN----SA-LANDS --- -GA--HT---KRYI--TIT--
Tauraco erythrolophus-Alpha-4K	XP_009982567.1	E--------FQN----SA-LANDS --- -GA--HT---KRYI--TIT--
Pygoscelis adeliae-Alpha-4K	XP_009328840.1	E--------FQN----SA-LANDS --- -GA--HT---KRYI--TIT--
Calidris pugnax-Alpha-4K	XP_014802059.1	E--------FQN----SA-LANDS --- -GA--HT---KRYI--TIT--
Pterocles gutturalis-Alpha-4K	KFV14977.1	E--------FQN----SA-LANDS --- -GA--HT---KRYI--TIT--
Calypte anna-Alpha-4K	XP_008490926.1	E--------FQN----SA-LANDS --- -GA--HT---KRYI--TIT--
Zonotrichia albicollis-Alpha-4K	XP_005480377.1	E--------FQN----SA-LANDS --- -GA--HT---KRYI--TIT--
Buceros rhinoceros silvestris-Alpha-4K	KFO84995.1	E--------FQN----SA-LANDS --- -GA--HT---KRYI--TIT--
Falco peregrinus-Alpha-4K	XP_013151911.1	E--------FQN----SA-LANDS --- -GA--HT---KRYI--TIT--
Columba livia-Alpha-4K	XP_005500853.1	E--------FQN----SA-LANDS --- -GA--HT---KRYI--TIT--
Taeniopygia guttata-Alpha-4K	XP_002191171.2	E--------FQN----SA-LANDS --- -GA--HT---KRYI--TIT--
Picoides pubescens-Alpha-4K	KFV76283.1	E--------FQN----SA-LANDS --- -GA--HT---KRYI--TIT--
Egretta garzetta-Alpha-4K	KFP14997.1	E--------FQN----SA-LANDS --- -GA--HT---KRYI--TIT--
Leptosomus discolor-Alpha-4K	KFQ02093.1	E--------FQN----SA-LANDS --- -GA--HT---KRYI--TIT--
Chaetura pelagica-Alpha-4K	XP_010005256.1	E--------FQN----SA-LANDS --- -GA--HT---KRYI--TIT--
Anas platyrhynchos-Alpha-4K	XP_021134965.1	E--------FQN----SA-LANDS --- -GA--HT---KRYI--TIT--
Nipponia nippon-Alpha-4K	XP_009467307.1	E--------FQN----SA-LANDS --- -GA--HT---KRYI--TIT--
Apteryx australis mantelli-Alpha-4K	XP_013804798.1	E--------FQN----SA-LANDS --- -GA--HT---KRYI--TIT--
Tinamus guttatus-Alpha-4K	XP_010212163.1	E--------FQN----SA-LANDS --- -GA--HT---KRYI--TIT--
Colius striatus-Alpha-4K	XP_010200239.1	E--------FQN----SA-LANDS --- -GA--HT---KRYI--TIT--
Corvus brachyrhynchos-Alpha-4K	XP_008637456.1	E--------FQN----SA-LANDS --- -GA--HT---KRYI--TIT--
Balearica regulorum gibbericep-Alpha-4K	KFO11315.1	E--------FQN----SA-LANDS --- -GA--HT---KRYI--TIT--
Struthio camelus australis-Alpha-4K	XP_009671943.1	E--------FQN----SA-LANDS --- -GA--HT---KRYI--TIT--
Numida meleagris-Alpha-4K	XP_021241582.1	E--------FQN----SA-LANDS --- -GA--HT---KRYI--TIT--
Coturnix japonica-Alpha-4K	XP_015708788.1	E--------FQN----SA-LANDS --- -GA--HT---KRYI--TIT--
Anser cygnoides domesticus-Alpha-4K	XP_013027517.1	E--------FQN----SA-LANDS --- -GA--HT---KRYI--TIT--
Callipepla squamata-Alpha-4K	OXB53668.1	E--------FQN----SA-LANDS --- -GA--HT---KRYI--TIT--
Serinus canaria-Alpha-4K	XP_018764876.1	E--------FQN----SA-LANDS --- -GA--HT---KRYI--TIT--
Opisthocomus hoazin-Alpha-4K	XP_009936207.1	E--------FQN----SA-LANDS --- -GA--HT---KRY---TIT--
Aptenodytes forsteri-Alpha-4K	XP_009273500.1	E--------FQN----SA-VANDS --- -GA--HT---KRYI--TIT--
Acanthisitta chloris-Alpha-4K	KFP80895.1	E--------FQN----SS-LANDS --- -GA--HT---KRYI--TIT--
Lonchura striata domestica-Alpha-4K	XP_021386060.1	E--------FQN----SA-LANDS --- -GA--HT---KRYI--TIT--
Colinus virginianus-Alpha-4K	OXB84665.1	E--------FQN----SA-LANDS --- -GA--HT---KRYI--TIT--
Eurypyga helias-Alpha-4K	XP_010146922.1	E--------FQN----SA-LANDS --- -GA--HT---KRY---SIT--

Amphibians PIP4Kα

Nanorana parkeri-Alpha-4K	XP_018419770.1	E--------FQN----CA-LANDS --- -GA--HT-C-KRYI--TIT--
Xenopus laevis-Alpha-4K	XP_018124586.1	E--------F-N----YS-LANDS --- -GA--HT-C-KRYI--TIT--
Rana catesbeiana-Alpha-4K	PIO13764.1	E---------QN----SA-VN--N -G- FGS---TTF--RFI--TI-G-
Xenopus tropicalis-Alpha-4K	NP_001123723.1	E--------F-N----YS-LANDS --- -GA--HT-C-KRYI--TIT—

Reptiles PIP4Kα

Gekko japonicus-Alpha-4K	XP_015262444.1	E--------FQN----SC-LANDS --- -GA--HT---KRY---TIT--
Pogona vitticeps-Alpha-4K	XP_020657359.1	E--------FQN----SC-LANDS -G- -SA--HT---KRY---TIT--
Anolis carolinensis-Alpha-4K	XP_003222207.1	E--A-----FQN----SC-LANDS --- -GA--HT---KRY---TIT--

Thamnophis sirtalis-Alpha-4K	XP_013923180.1	E--------FQN----SC-LANDS A-- -GA--HS---KRY---TIT--
Python bivittatus-Alpha-4K	XP_007436413.1	E--------FQN----SC-LANDS P-- -GA--HS---KRY---TIT--
Pogona vitticeps-Alpha-4K	XP_020657367.1	E--------FQN----SC-LANDS -G- -SA--HT---KRY---TIT--
Protobothrops mucrosquamatus-Alpha-4K	XP_015667052.1	E--------FQN----SC-LANDS P-- -GA--HS---KRY---TIT--

Fishes PIP4Kα

Danio rerio-Alpha-4K	NP_001122174.1	E--V-----FQN----SA-LV--A -G- -GA--HT---KRY---TI---
Oryzias latipes-Alpha-4K	XP_004081045.1	E-------EFSN----SA-LN-DA -G- -GA--HT---KRY---TI---
Oreochromis niloticus-Alpha-4K	XP_019218741.1	E--------F-N----SA-LN--A -G- -GA--HT---KRY---TI---
Haplochromis burtoni-Alpha-4K	XP_005912140.1	E--------F-N----SA-LN--A -G- -GA--HT---KRY---TI---
Takifugu rubripes-Alpha-4K	XP_003967903.1	E--------F-N----SA-LN--A -G- -GA--HT---KRY---TI---
Amphiprion ocellaris-Alpha-4K	XP_023127853.1	E--------F-N----SA-LN--A -G- -GA--HT---KRY---TI---
Paralichthys olivaceus-Alpha-4K	XP_019943171.1	E--------F-N----SA-LN--A -G- -GA--HT---KRY---TI---
Lates calcarifer-Alpha-4K	XP_018557492.1	E--------F-N----SA-LN--A -G- -GA--HT---KRY---TI---
Austrofundulus limnaeus-Alpha-4K	XP_013863896.1	E-------EFSN----SA-LN--A -G- -GA--HT---KRY---TI---
Larimichthys crocea-Alpha-4K	XP_010730941.1	E--C------QN----SA-LN-DS -G- FGN---S---HRF---T----

Mammals PIP4Kβ

Homo sapiens-Beta-4K	EAW60533.1	E---------QN-V--SA-IN-DS -G- CGT---TT---RF---T----
Rousettus aegyptiacus-Beta-4K	XP_016020207.1	E---------QN-V--SA-IN-DS -G- CGT---TT---RF---T----
Pantholops hodgsonii-Beta-4K	XP_005965028.1	E---------QN-V--SA-IN-DS -G- CGT---TT---RF---T----
Chlorocebus sabaeus-Beta-4K	XP_008011093.1	E---------QN-V--SA-IN-DS -G- CGT---TT---RF---T----
Cricetulus griseus-Beta-4K	XP_003499653.1	E---------QN-V--SA-IN-DS -G- CGT---TT---RF---T----
Tupaia chinensis-Beta-4K	XP_006156082.1	E---------QN-V--SA-IN-DS -G- CGT---TT---RF---T----
Ailuropoda melanoleuca-Beta-4K	EFB19938.1	E---------QN-V--SA-IN-DS -G- CGT---TT---RF---T----
Papio anubis-Beta-4K	XP_021784845.1	E---------QN-V--SA-IN-DS -G- CGT---TT---RF---T----
Heterocephalus glaber-Beta-4K	EHB09675.1	E---------QN-V--SA-IN-DS -G- CGT---TT---RF---T----
Bos mutus-Beta-4K	XP_005898119.1	E---------QN-V--SA-IN-DS -G- CGT---TT---RF---T----
Mus musculus-Beta-4K	AAL18245.1	E---------QN-V--SA-IN-DS -G- CGT---TT---RF---T----
Acinonyx jubatus-Beta-4K	XP_014933988.1	E---------QN-V--SA-IN-DS -G- CGT---TT---RF---T----
Saimiri boliviensis boliviensi-Beta-4K	XP_010340117.1	E---------QN-V--SA-IN-DS -G- CGT---TT---RF---T----
Equus przewalskii-Beta-4K	XP_008521513.1	E---------QN-V--SA-IN-DS -G- CGT---TT---RF---T----
Marmota marmota marmota-Beta-4K	XP_015362142.1	E---------QN-V--SA-IN-DS -G- CGT---TT---RF---T----
Tursiops truncatus-Beta-4K	XP_019780539.1	E---------QN-V--SA-IN-DS -G- CGT---TT---RF---T----
Delphinapterus leucas-Beta-4K	XP_022413616.1	E---------QN-V--SA-IN-DS -G- CGT---TT---RF---T----
Mustela putorius furo-Beta-4K	XP_012917975.1	E---------QN-V--SA-IN-DS -G- CGT---TT---RF---T----
Lipotes vexillifer-Beta-4K	XP_007465206.1	E---------QN-V--SA-IN-DS -G- CGT---TT---RF---T----
Echinops telfairi-Beta-4K	XP_004707158.1	E---------QN-V--SA-IN-DS -G- CGT---TT---RF---T----
Rhinolophus sinicus-Beta-4K	XP_019596211.1	E---------QN-V--SA-IN-DS -G- CGT---TT---RF---T----
Bison bison bison-Beta-4K	XP_010841435.1	E---------QN-V--SA-IN-DS -G- CGT---TT---RF---T----
Erinaceus europaeus-Beta-4K	XP_016047112.1	E---------QN-V--SA-IN-DS -G- CGT---TT---RF---T----
Mus pahari-Beta-4K	XP_021068416.1	E---------QN-V--SA-IN-DS -G- CGT---TT---RF---T----
Bubalus bubalis-Beta-4K	XP_006074405.1	E---------QN-V--SA-IN-DS -G- CGT---TT---RF---T----
Elephantulus edwardii-Beta-4K	XP_006889429.1	E---------QN-V--SA-IN-DS -G- CGT---TT---RF---T----
Odobenus rosmarus divergens-Beta-4K	XP_004395129.1	E---------QN-V--SA-IN-DS -G- CGT---TT---RF---T----
Miniopterus natalensis-Beta-4K	XP_016057782.1	E---------QN-V--SA-IN-DS -G- CGT---TT---RF---T----
Panthera tigris altaica-Beta-4K	XP_007085822.1	E---------QN-V--SA-IN-DS -G- CGT---TT---RF---T----
Odocoileus virginianus texanus-Beta-4K	XP_020735216.1	E---------QN-V--SA-IN-DS -G- CGT---TT---RF---T----
Canis lupus familiaris-Beta-4K	XP_022278739.1	E---------QN-V--SA-IN-DS -G- CGT---TT---RF---T----
Castor canadensis-Beta-4K	XP_020027849.1	E---------QN-V--SA-IN-DS -G- CGT---TT---RF---T----
Orcinus orca-Beta-4K	XP_004282755.1	E---------QN-V--SA-IN-DS -G- CGT---TT---RF---T----
Loxodonta africana-Beta-4K	XP_010592684.1	E---------QN-V--SA-IN-DS -G- CGT---TT---RF---T----
Ursus maritimus-Beta-4K	XP_008687183.1	E---------QN-V--SA-IN-DS -G- CGT---TT---RF---T----
Oryctolagus cuniculus-Beta-4K	XP_008269611.2	E---------QN-V--SA-IN-DS -G- CGT---TT---RF---T----
Pteropus alecto-Beta-4K	XP_006925185.1	E---------QN-V--SA-IN-DS -G- CGT---TT---RF---T----
Ovis aries-Beta-4K	XP_012041405.1	E---------QN-V--SA-IN-DS -G- CGT---TT---RF---T----
Felis catus-Beta-4K	XP_019673360.2	E---------QN-V--SA-IN-DS -G- CGT---TT---RF---T----
Hipposideros armiger-Beta-4K	XP_019494299.1	E---------QN-V--SA-IN-DS -G- CGT---TT---RF---T----
Chrysochloris asiatica-Beta-4K	XP_006832597.1	E---------QN-V--SA-IN-DS -G- CGT---TT---RF---T----
Propithecus coquereli-Beta-4K	XP_012518294.1	E---------QN-V--SA-IN-DS -G- CGT---TT---RF---T----
Otolemur garnettii-Beta-4K	XP_003786482.1	E---------QN-V--SA-IN-DS -G- CGT---TT---RF---T----
Mandrillus leucophaeus-Beta-4K	XP_011840098.1	E---------QN-V--SA-IN-DS -G- CGT---TT---RF---T----
Saimiri boliviensis boliviensi-Beta-4K	XP_010340118.1	E---------QN-V--SA-IN-DS -G- CGT---TT---RF---T----
Neotoma lepida-Beta-4K	OBS76957.1	E---------QN-V--SA-IN-DS -G- CGT---TT---RF---T----
Dipodomys ordii-Beta-4K	XP_012892556.1	E---------QN-V--SA-IN-DS -G- CGT---TT---RF---T----
Trichechus manatus latirostris-Beta-4K	XP_004378067.1	E---------QN-V--SA-IN-DS -G- CGT---TT---RF---T----
Bos taurus-Beta-4K	NP_001179125.1	E---------QN-V--SA-IN-DS -G- CGT---TT---RF---T----
Orycteropus afer afer-Beta-4K	XP_007940200.1	E---------QN-V--SA-IN-DS -G- CGT---TT---RF---T----
Peromyscus maniculatus bairdii-Beta-4K	XP_006971925.1	E---------QN-V--SA-IN-DS -G- CGT---TT---RF---T----
Ceratotherium simum simum-Beta-4K	XP_004434649.1	E---------QN-V--SA-IN-DS -G- CGT---TT---RF---T----
Eptesicus fuscus-Beta-4K	XP_008152007.1	E---------QN-V--SA-IN-DS -G- CGT---TT---RF---T----
Leptonychotes weddellii-Beta-4K	XP_006743367.1	E---------QN-V--SA-IN-DS -G- CGT---TT---RF---T----
Ictidomys tridecemlineatus-Beta-4K	XP_005321814.1	E---------QN-V--SA-IN-DS -G- CGT---TT---RF---T----
Dasypus novemcinctus-Beta-4K	XP_004454989.1	E---------QN-V--SA-IN-DS -G- CGT---TT---RF---T----
Microtus ochrogaster-Beta-4K	XP_005368369.1	E---------QN-V--SA-IN-DS -G- CGT---TT---RF---T----
Sorex araneus-Beta-4K	XP_004608775.1	E---------QN-V--SA-IN-DS -G- CGT---TT---RF---T----
Ochotona princeps-Beta-4K	XP_004591183.1	E---------QN-V--SA-IN-DS -G- CGT---TT---RF---T----

Galeopterus variegatus-Beta-4K	XP_008579047.1	E---------QN-V--SA-IN-DS -G- CGT---TT---RF---T----
Colobus angolensis palliatus-Beta-4K	XP_011815221.1	E---------QN-V--SA-IN-DS -G- CGT---TT---RF---T----
Piliocolobus tephrosceles-Beta-4K	XP_023060061.1	E---------QN-V--SA-IN-DS -G- CGT---TT---RF---T----
Ursus maritimus-Beta-4K	XP_008687191.1	E---------QN-V--SA-IN-DS -G- CGT---TT---RF---T----
Castor canadensis-Beta-4K	XP_020027850.1	E---------QN-V--SA-IN-DS -G- CGT---TT---RF---T----
Homo sapiens-Beta-4K	NP_003550.1	E---------QN-V--SA-IN-DS -G- CGT---TT---RF---T----
Bubalus bubalis-Beta-4K	XP_006074406.1	E---------QN-V--SA-IN-DS -G- CGT---TT---RF---T----
Jaculus jaculus-Beta-4K	XP_004655548.1	E---------QN-V--SA-IN-DS -G- CGT---TT---RF---T----
Rattus norvegicus-Beta-4K	NP_446002.1	E---------QN-V--SA-IN-DS -G- CGT---TT---RF---T----
Panthera tigris altaica-Beta-4K	XP_015394625.1	E---------QN-V--SA-IN-DS -G- CGT---TT---RF---T----
Condylura cristata-Beta-4K	XP_004684324.1	E---------QN-V--SA-VN-DS -G- CGT---TT---RF---T----
Myotis lucifugus-Beta-4K	XP_006100821.1	E---------QN-V--SA-IN-DS -G- CGT---TT---RF---T----
Mus caroli-Beta-4K	XP_021033032.1	E---------QN-V--SA-IN-DS -G- CGT---TT---RF---T----
Neomonachus schauinslandi-Beta-4K	XP_021560049.1	E---------QN-V--SA-IN-DS -G- CGT---TT---RF---T----
Felis catus-Beta-4K	XP_019673361.1	E---------QN-V--SA-IN-DS -G- CGT---TT---RF---T----
Equus asinus-Beta-4K	XP_014689184.1	E---------QN-V--SA-IN-DS -G- CGT---TT---RF---T----
Capra hircus-Beta-4K	XP_017920477.1	E---------QN-V--SA-IN-DS -G- CGT---TT---RF---T----
Callithrix jacchus-Beta-4K	XP_017828202.1	E---------QN-V--SA-IN-DS -G- CGT---TT---RF---T----
Macaca fascicularis-Beta-4K	EHH58054.1	E---------QN-V--SA-IN-DS -G- CGT---TT---RF---T----
Mesocricetus auratus-Beta-4K	XP_005076083.1	E---------QN-V--SA-IN-DS -G- CGT---TT---RF---T----
Fukomys damarensis-Beta-4K	XP_019062456.1	E---------QN-V--SA-IN-DS -G- CGT---TT---RF---T----
Cricetulus griseus-Beta-4K	XP_016822589.1	E---------QN-V--SA-IN-DS -G- CGT---TT---RF---T----
Carlito syrichta-Beta-4K	XP_008064634.1	E---------QN-V--SA-IN-DS -G- CGT---TT---RF---T----
Pan paniscus-Beta-4K	XP_008960384.1	E---------QN-V--SA-IN-DS -G- CGT---TT---RF---T----
Cebus capucinus imitator-Beta-4K	XP_017352721.1	E---------QN-V--SA-IN-DS -G- CGT---TT---RF---T----
Sarcophilus harrisii-Beta-4K	XP_023358122.1	E---------QN-V--SA-IN-DS -G- CGT---TT---RF---A----
Phascolarctos cinereus-Beta-4K	XP_020852981.1	E---------QN-V--SA-IN-DS -G- CGT---TT---RF---A----
Cavia porcellus-Beta-4K	XP_013005043.1	E--A------QN-V--SA-IN-DS -G- CGT---TT---RF---T----
Monodelphis domestica-Beta-4K	XP_001366640.1	E---------QN-V--SA-VN-DS -G- CGT---TT---RF---A----
Camelus bactrianus-Beta-4K	XP_010958800.1	E--A------QN-V--SA-IN-DS -G- CGT---TT---RF---T----
Chinchilla lanigera-Beta-4K	XP_013374727.1	E--A------QN-V--SA-IN-DS -G- CGT---TT---RF---T----
Manis javanica-Beta-4K	XP_017500215.1	E---------QN-V--SA-IN-DS -G- CGTH--TT---RF---T----
Octodon degus-Beta-4K	XP_004633743.1	E--A------QN-V--SA-IN-DS -G- CGT---TT---RF---T----
Enhydra lutris kenyoni-Beta-4K	XP_022365086.1	E---------Q-RV--SA-IN-DS -G- CGT---TT---RF---T----
Rhinolophus sinicus-Beta-4K	XP_019596557.1	E--------FQN----SA-LPNDS --- -GA--HT---KRY---TIT--
**Birds PIP4Kβ**		
Bambusicola thoracicus-Beta-4K	POI23438.1	E---------QN-V--SA-VN-DS -G- CGA---TT---RF---A----
Sturnus vulgaris-Beta-4K	XP_014740843.1	E---------QN-V--SA-VN-DS -G- CGA---TT---RF---A----
Corvus brachyrhynchos-Beta-4K	XP_008633051.1	E---------QN-V--SA-VN-DS -G- CGA---TT---RF---A----
Acanthisitta chloris-Beta-4K	XP_009067947.1	E---------QN-V--SA-VN-DS -G- CGA---TT---RF---A----
Apaloderma vittatum-Beta-4K	XP_009867625.1	E---------QN-V--SA-VN-DS -G- CGA---TT---RF---A----
Picoides pubescens-Beta-4K	KFV69749.1	E---------QN-V--SA-VN-DS -G- CGA---TT---RF---A----
Cyanistes caeruleus-Beta-4K	XP_023798430.1	E---------QN-V--SA-VN-DS -G- CGA---TT---RF---A----
Struthio camelus australis-Beta-4K	XP_009679369.1	E---------QN-V--SA-VN-DS -G- CGA---TT---RF---A----
Anser cygnoides domesticus-Beta-4K	XP_013055874.1	E---------QN-V--SA-VN-DS -G- CGA---TT---RF---A----
Columba livia-Beta-4K	XP_021151168.1	E---------QN-V--SA-VN-DS -G- CGA---TT---RF---A----
Aquila chrysaetos canadensis-Beta-4K	XP_011592357.1	E---------QN-V--SA-VN-DS -G- CGA---TT---RF---A----
Lepidothrix coronata-Beta-4K	XP_017693394.1	E---------QN-V--SA-VN-DS -G- CGA---TT---RF---A----
Gallus gallus-Beta-4K	XP_015155005.1	E---------QN-V--SA-VN-DS -G- CGA---TT---RF---A----
Numida meleagris-Beta-4K	XP_021233875.1	E---------QN-V--SA-VN-DS -G- CGA---TT---RF---A----
Haliaeetus leucocephalus-Beta-4K	XP_010583833.1	E---------QN-V--SA-VN-DS -G- CGA---TT---RF---A----
Patagioenas fasciata monilis-Beta-4K	OPJ74427.1	E---------QN-V--SA-VN-DS -G- CGA---TT---RF---A----
Coturnix japonica-Beta-4K	XP_015741210.1	E---------QN-V--SA-VN-DS -G- CGA---TT---RF---A----
Tinamus guttatus-Beta-4K	XP_010213328.1	E---------QN-V--SA-VN-DS -G- CGA---TT---RF---A----
Manacus vitellinus-Beta-4K	XP_017923222.1	E---------QN-V--SA-VN-DS -G- CGA---TT---RF---A----
Callipepla squamata-Beta-4K	OXB63642.1	E---------QN-V--SA-VN-DS -G- CGA---TT---RF---A----
Pseudopodoces humilis-Beta-4K	XP_005531464.1	E---------QN-V--SA-VN-DS -G- CGA---TT---RF---A----
Zonotrichia albicollis-Beta-4K	XP_005496888.2	E---------QN-V--SA-VN-DS -G- CGA---TT---RF---A----
Serinus canaria-Beta-4K	XP_009094714.2	E---------QN-V--SA-VN-DS -G- CGA---TT---RF---A----
Aptenodytes forsteri-Beta-4K	XP_009273130.1	E---------QN-V--SA-VN-DS -G- CGA---TT---RF---A----
Meleagris gallopavo-Beta-4K	XP_010722908.1	E---------QN-V--SA-VN-DS -G- CGA---TT---RF---A----
Geospiza fortis-Beta-4K	XP_005430606.1	E---------QN-V--SA-VN-DS -G- CGA---TT---RF---A----
Nestor notabilis-Beta-4K	XP_010010237.1	E---------QN-V--SA-VN-DS -G- CGA---TT---RF---A----
Falco peregrinus-Beta-4K	XP_013153929.1	E---------QN-V--SA-VN-DS -G- CGA---TT---RF---A----
Ficedula albicollis-Beta-4K	XP_016160540.1	E---------QN-V--SA-VN-DS -G- CGA---TT---RF---A----
Colinus virginianus-Beta-4K	OXB69681.1	E---------QN-V--SA-VN-DS -G- CGA---TT---RF---A----
Anas platyrhynchos-Beta-4K	EOA95554.1	E---------QN-V--SA-VN-DS -G- CGA---TT---RF---A----
Eurypyga helias-Beta-4K	KFW07279.1	E---------QN-V--SA-VN-DS -G- CGA---TT---RF---A----
Opisthocomus hoazin-Beta-4K	KFR08354.1	E---------QN-V--SA-VN-DS -G- CGA---TT---RF---A----
Phaethon lepturus-Beta-4K	KFQ72396.1	E---------QN-V--SA-VN-DS -G- CGA---TT---RF---A----
Calypte anna-Beta-4K	KFO97476.1	E---------QN-V--SA-VN-DS -G- CGA---TT---RF---A----
Merops nubicus-Beta-4K	KFQ34921.1	E---------QN-V--SA-VN-DS -G- CGA---TT---RF---A----
Chaetura pelagica-Beta-4K	KFU89343.1	E---------QN-V--SA-VN-DS -G- CGA---TT---RF---A----
Amazona aestiva-Beta-4K	KQK77230.1	E---------QN-V--SA-VN-DS -G- CGA---TT---RF---A----
Chlamydotis macqueenii-Beta-4K	KFP42939.1	E---------QN-V--SA-VN-DS -G- CGA---TT---RF---A----

Apteryx australis mantelli-Beta-4K	XP_013812931.1	E---------QN-V--SA-VN-DS -G- CGA---TT---RF---A----
Melopsittacus undulatus-Beta-4K	XP_005140623.1	E---------QN-V--SA-VN-DS -G- CGA---TT---RF---A----

Amphibians PIP4Kβ

Rana catesbeiana-Beta-4K	PIO13764.1	E---------QN----SA-VN--N -G- FGS---TTF--RFI--TI-G-
Xenopus tropicalis-Beta-4K	XP_002940195.1	E---------QN----SA-VN--N -G- FGS---TT---RF---TI---
Nanorana parkeri-Beta-4K	XP_018428736.1	E---------QN----SA-VN--N -G- FGS---TTF--RFI--TI-G-
Xenopus laevis-Beta-4K	AAM18511.1	E---------QN----SA-VN--N LG- FGS---TT---RF---TI-G-

Reptiles PIP4Kβ

Protobothrops mucrosquamatus-Beta-4K	XP_015684863.1	E---------QN-V--SA-VY-DS HG- CGV---T----RF---A----
Pogona vitticeps-Beta-4K	XP_020670192.1	E---------QN-V--SA-VY-DS HG- CGV---TT---RF---A----
Thamnophis sirtalis-Beta-4K	XP_013913720.1	E---------QN-V--SA-VYNDS HG- CGV---TT---RF---A----
Anolis carolinensis-Beta-4K	XP_003222566.1	E---------QN-V--SA-VY--S HG- CGV---TT---RF---A----
Ophiophagus hannah-Beta-4K	ETE71454.1	E---------QN-V--SA-VYNDS HG- CGV---TT---RF---A----
Gekko japonicus-Beta-4K	XP_015279045.1	E---------QN-V--SA-VYTDS HG- CGVH--TT---RF---A----

Fishes PIP4Kβ

Oryzias latipes-Beta-4K	XP_004084068.1	E---------QN----SA-LN-DS -G- FGN---C---HRF---TI---
Takifugu rubripes-Beta-4K	XP_011610877.1	E--C------QN----SA-LN-DS -G- FGN---TT--HRF---T----
Austrofundulus limnaeus-Beta-4K	XP_013877104.1	E--C------QN----SA-IN-DS -G- FGN---T---HRF---T----
Lates calcarifer-Beta-4K	XP_018533977.1	E--C------QN----SA-LN-DS -G- FGS---S---HRF---T----
Amphiprion ocellaris-Beta-4K	XP_023135729.1	E--C------QN----SA-LN-DS -G- FGN---S---HRF---T----
Larimichthys crocea-Beta-4K	XP_010730941.1	E--C------QN----SA-LN-DS -G- FGN---S---HRF---T----
Paralichthys olivaceus-Beta-4K	XP_019939731.1	E--C------QN----SA-LN-DS -G- FGN---S---HRF---T----
Haplochromis burtoni-Beta-4K	XP_005919307.1	E--C------QN----SA-LN-DT -G- FGN-I-S---HRF---T----
Oreochromis niloticus-Beta-4K	XP_003448020.1	E--C------QN----SA-LN-DT -G- FGN-I-S---HRF---T----

Mammals PIP5Kα

Mus musculus-Alpha-5K	AAH03763.1	EL---RPD---Y--CSE-LI-LSN SGA -GSL-YV-S-DEFI--T-QHK
Homo sapiens-Alpha-5K	XP_011526152.1	EL---RPD---Y--CNE-LI-LSN PGA -GSL-YVTS-DEFI--T-MHK
Cricetulus griseus-Alpha-5K	ERE90766.1	EL---RPD---Y--CSE-LI-LSN SGA -GSL-YV-S-DEFI--T-QHK
Mus pahari-Alpha-5K	XP_021052610.1	EL---RPD---Y--CSE-LI-LSN SGA -GSL-YV-S-DEFI--T-QHK
Mus caroli-Alpha-5K	XP_021013845.1	EL---RPD---Y--CSE-LI-LSN SGA -GSL-YV-S-DEFI--T-QHK
Rattus norvegicus-Alpha-5K	XP_008759537.1	EL---RPD---Y--CSE-LI-LSN SGA -GSL-YV-S-DEFI--T-QHK
Chinchilla lanigera-Alpha-5K	XP_013365384.1	EL---RPD---Y--CSE-LI-LSN SGA -GSL-YV-S-DEFI--T-QHK
Microtus ochrogaster-Alpha-5K	XP_005357052.1	EL---RPD---Y--CSE-LI-LSN SGA -GSL-YV-S-DEFI--T-QHK
Myotis davidii-Alpha-5K	ELK36257.1	EL---RPD---Y--CSE-LI-LCN SGA -GSL-YV-S-DEFI--T-QHK
Peromyscus maniculatus bairdii-Alpha-5K	XP_015856564.1	EL---RPD---Y--CSE-LI-LSN SGA -GSL-YV-S-DEFI--T-QHK
Neotoma lepida-Alpha-5K	OBS79227.1	EL---RPD---Y--CNE-LI-LSN SGA -GSL-YV-S-DEFI--T-QHK
Ochotona princeps-Alpha-5K	XP_012783501.1	EL---RPD---Y--CSE-LI-LCN SGA -GSL-YV-S-DEFI--T-QHK
Mesocricetus auratus-Alpha-5K	XP_012979239.2	EL---RPD---Y--CNE-LI-LSN SGA -GSL-YV-S-DEFI--T-QHK
Pan troglodytes-Alpha-5K	PNI61986.1	EL---KPD---Y-ICSE-LI-LSN PGA -GSL-FVTS-DEFI--T-QHK
Pteropus vampyrus-Alpha-5K	XP_023382396.1	EL---RPD---Y--CNE-LI-LSN PGA -GSL-YVTS-DEFI--T-MHK
Meriones unguiculatus-Alpha-5K	XP_021512505.1	EL---RPD---Y--CSE-LI-LCN SGA -GSL-YV-S-DEFI--T-QHK
Enhydra lutris kenyoni-Alpha-5K	XP_022381744.1	EL---RPD---Y--CSE-LI-LCN SGA -GSL-YV-S-DEFI--T-QHK
Ailuropoda melanoleuca-Alpha-5K	XP_011223430.1	EL---RPD---Y--CSE-LI-LCN SGA -GSL-YV-S-DEFI--T-QHK
Sarcophilus harrisii-Alpha-5K	XP_012405199.1	EL---RPD---Y--CSE-LI-LCN SGA -GSL-YV-S-DEFI--T-QHK
Ursus maritimus-Alpha-5K	XP_008693896.1	EL---RPD---Y--CSE-LI-LCN SGA -GSL-YV-S-DEFI--T-QHK
Echinops telfairi-Alpha-5K	XP_012863748.1	EL---RPD---Y--CSE-LI-LCN SGA -GSL-YV-S-DEFI--T-QHK
Oryctolagus cuniculus-Alpha-5K	ACJ74014.1	EL---RPD---Y--CSE-LI-LCN SGA -GSL-YV-S-DEFI--T-QHK
Leptonychotes weddellii-Alpha-5K	XP_006728426.1	EL---RPD---Y--CSE-LI-LCN SGA -GSL-YV-S-DEFI--T-QHK
Octodon degus-Alpha-5K	XP_023572206.1	EL---RPD---Y--CSE-LI-LCN SGA -GSL-YV-S-DEFI--T-QHK
Neomonachus schauinslandi-Alpha-5K	XP_021543791.1	EL---RPD---Y--CSE-LI-LCN SGA -GSL-YV-S-DEFI--T-QHK
Odobenus rosmarus divergens-Alpha-5K	XP_004404309.1	EL---RPD---Y--CSE-LI-LCN SGA -GSL-YV-S-DEFI--T-QHK
Heterocephalus glaber-Alpha-5K	XP_012931038.1	EL---RPD---Y--CSE-LI-LCN SGA -GSL-YV-S-DEFI--T-QHK
Hipposideros armiger-Alpha-5K	XP_019480853.1	EL---RPD---Y--CSE-LI-LCN SGA -GSL-YV-S-DEFI--T-QHK
Fukomys damarensis-Alpha-5K	XP_010614785.1	EL---RPD---Y--CSE-LI-LCN SGA -GSL-YV-S-DEFI--T-QHK
Mustela putorius furo-Alpha-5K	XP_004776794.1	EL---RPD---Y--CSE-LI-LCN SGA -GSL-YV-S-DEFI--T-QHK
Myotis brandtii-Alpha-5K	XP_014384117.1	EL---RPD---Y--CSE-LI-LCN SGA -GSL-YV-S-DEFI--T-QHK
Sus scrofa-Alpha-5K	NP_001231380.1	EL---RPD---Y--CSE-LI-LCN SGA -GSL-YV-S-DEFI--T-QHK
Camelus ferus-Alpha-5K	XP_006186704.1	EL---RPD---Y--CSE-LI-LCN SGA -GSL-YV-S-DEFI--T-QHK
Myotis lucifugus-Alpha-5K	XP_014316802.1	EL---RPD---Y--CSE-LI-LCN SGA -GSL-YV-S-DEFI--T-QHK
Tupaia chinensis-Alpha-5K	ELV10744.1	EL---RPD---Y--CSE-LI-LCN SGA -GSL-YV-S-DEFI--T-QHK
Callithrix jacchus-Alpha-5K	ABY82090.1	EL---RPD---Y--CSE-LI-LCN SGA -GSL-YV-S-DEFI--T-QHK
Rhinolophus ferrumequinum-Alpha-5K	ACC64588.1	EL---RPD---Y--CSE-LI-LCN SGA -GSL-YV-S-DEFI--T-QHK
Camelus bactrianus-Alpha-5K	XP_010956742.1	EL---RPD---Y--CSE-LI-LCN SGA -GSL-YV-S-DEFI--T-QHK
Eptesicus fuscus-Alpha-5K	XP_008154086.1	EL---RPD---Y--CSE-LI-LCN SGA -GSL-YV-S-DEFI--T-QHK
Camelus dromedarius-Alpha-5K	XP_010995069.1	EL---RPD---Y--CSE-LI-LCN SGA -GSL-YV-S-DEFI--T-QHK
Jaculus jaculus-Alpha-5K	XP_012806279.1	EL---RPD---Y--CSE-LI-LCN SGA -GSL-YV-S-DEFI--T-QHK
Ictidomys tridecemlineatus-Alpha-5K	XP_013216632.1	EL---RPD---Y--CSE-LI-LCN SGA -GSL-YV-S-DEFI--T-QHK
Marmota marmota marmota-Alpha-5K	XP_015358432.1	EL---RPD---Y--CSE-LI-LCN SGA -GSL-YV-S-DEFI--T-QHK

Birds PIP5Kα

Geospiza fortis-Alpha-5K	XP_014167056.1	EL---RPD---Y--CSE-LI-LSN SGA -GSL-YV-S-DEFI--T-QHK
Cathartes aura-Alpha-5K	KFP49378.1	EL---RPD---Y--CSE-LI-LSN SGA -GSL-YV-S-DEFI--T-QHK
Cuculus canorus-Alpha-5K	KFO73190.1	EL---RPD---Y--CSE-LI-LSN SGA -GSL-YV-S-DEFI--T-QHK
Phoenicopterus ruber ruber-Alpha-5K	KFQ81007.1	EL---RPD---Y--CSE-LI-LSN SGA -GSL-YV-S-DEFI--T-QHK
Amazona aestiva-Alpha-5K	KQL59296.1	EL---RPD---Y--CSE-LI-LSN SGA -GSL-YV-S-DEFI--T-QHK

Phaethon lepturus-Alpha-5K	KFQ69620.1	EL---RPD---Y--CSE-LI-LSN SGA -GSL-YV-S-DEFI--T-QHK
Melopsittacus undulatus-Alpha-5K	XP_012985591.1	EL---RPD---Y--CSE-LI-LSN SGA -GSL-YV-S-DEFI--T-QHK
Numida meleagris-Alpha-5K	XP_021232499.1	EL---RPD---Y--CSE-LI-LSN SGA -GSL-YV-S-DEFI--T-QHK
Coturnix japonica-Alpha-5K	XP_015740163.1	EL---RPD---Y--CSE-LI-LSN SGA -GSL-YV-S-DEFI--T-QHK
Meleagris gallopavo-Alpha-5K	XP_010722285.1	EL---RPD---Y--CSE-LI-LSN SGA -GSL-YV-S-DEFI--T-QHK
Anas platyrhynchos-Alpha-5K	XP_021132788.1	EL---RPD---Y--CSE-LI-LSN SGA -GSL-YV-S-DEFI--T-QHK
Antrostomus carolinensis-Alpha-5K	XP_010163162.1	EL---RPD---Y--CSE-LI-LSN SGA -GSL-YV-S-DEFI--T-QHK
Anser cygnoides domesticus-Alpha-5K	XP_013056952.1	EL---RPD---Y--CSE-LI-LSN SGA -GSL-YV-S-DEFI--T-QHK
Picoides pubescens-Alpha-5K	KFV76585.1	EL---RPD---Y--CNE-LI-LSN SGA -GSL-YV-S-DEFI--T-QHK
Podiceps cristatus-Alpha-5K	KFZ67268.1	EL---RPD---Y--CNE-LI-LSN SGA -GSL-YV-S-DEFI--T-QHK
Cariama cristata-Alpha-5K	KFP65666.1	EL---RPD---Y--CNE-LI-LSN SGA -GSL-YV-S-DEFI--T-QHK
Gallus gallus-Alpha-5K	NP_001135912.2	EL---RPD---Y--CSE-LI-LSN SGA -GSL-YV-S-DEFI--T-QHK
Colinus virginianus-Alpha-5K	OXB72319.1	EL---RPD---Y--CSE-LI-LSN SGA -GSL-YV-S-DEFI--T-QHK
Meleagris gallopavo-Alpha-5K	XP_010722283.1	EL---RPD---Y--CSE-LI-LSN SGA -GSL-YV-S-DEFI--T-QHK
Aptenodytes forsteri-Alpha-5K	KFM08822.1	EL---RPD---Y--CNE-LI-LSN SGA -GSL-YV-S-DEFI--T-QHK
Callipepla squamata-Alpha-5K	OXB55834.1	EL---RPD---Y--CSE-LI-LSN SGA -GSL-YV-S-DEFI--T-QHK
Nipponia nippon-Alpha-5K	XP_009465378.1	EL---RPD---Y--CSE-LI-LSN SGA -GSL-YV-S-DEFI--T-RHK
Charadrius vociferus-Alpha-5K	KGL86760.1	EL---RPD---Y--CSE-LI-LSN SGA -GSL-YV-S-DEFI--TAQHK
Columba livia-Alpha-5K	XP_021136061.1	EL---RPD---Y--CNE-LI-LSN SGA -GSL-YV-S-DEFI--T-QHK
Serinus canaria-Alpha-5K	XP_018781091.1	EL---RPD---Y--CSE-LI-LSN SGA -GSL-YV-S-DEFI--T-QHK
Aquila chrysaetos canadensis-Alpha-5K	XP_011596815.1	EL---RPD---Y--CNE-LI-LSN SGA -GSL-YV-S-DEFI--T-QHK
Calypte anna-Alpha-5K	KFO96662.1	EL---RPD---Y--CNE-LI-LSN SGA -GSL-YV-G-DEFI--T-QHK
Haliaeetus leucocephalus-Alpha-5K	XP_010563784.1	EL---RPD---Y--CNE-LI-LSN SGA -GSL-YV-S-DEFI--T-QHK
Chaetura pelagica-Alpha-5K	XP_010001104.1	EL---RPD---Y--CNE-LI-LSN SGA -GSL-YV-G-DEFI--T-QHK
Manacus vitellinus-Alpha-5K	KFW74318.1	EL---RPD---Y--CSE-LI-LSN SGA -GSL-YV-G-DEFI--T-QHK
Balearica regulorum gibbericep-Alpha-5K	KFO15204.1	EL---RPD---Y--CNE-LI-LSN SGA -GSL-YV-S-DKFI--T-QHK
Eurypyga helias-Alpha-5K	KFW07847.1	EL---RPD---Y--CND-LI-LSN SGA -GSL-YV-S-DEFI--T-QHK
Acanthisitta chloris-Alpha-5K	KFP88795.1	EL---RPD---Y--CNE-LI-LSN SGA -GSL-YV-G-DEFI--T-QHK
Struthio camelus australis-Alpha-5K	KFV72556.1	EL---RPD---Y--CND-LI-LSN SGA -GSL-YV-S-DEFI--T-QHK
Nestor notabilis-Alpha-5K	KFQ47794.1	EL---RPD---Y--CNE-LI-LSN SGA -GSL-YV-S-DEFI--K-QHK
Gavia stellata-Alpha-5K	KFV53654.1	EL---RPD---Y--CNE-LI-LSN SGA -GSL-YV-S-DEFI--K-QHK
Pseudopodoces humilis-Alpha-5K	XP_005534317.1	EL---RPD---Y--CND-LI-LSN SGA -GSL-YV-S-DEFI--T-QHK
Apteryx australis mantelli-Alpha-5K	XP_013798562.1	EL---RPD---Y--CND-LI-LSN SGA -GSL-YV-S-DEFI--T-QHK
Chlamydotis macqueenii-Alpha-5K	KFP45876.1	EL---RPD---Y--CNE-LI-LSN SGA -GSL-YV-S-DEFI--T-PHK
Mesitornis unicolor-Alpha-5K	KFQ32940.1	EL---RPD---Y--CNE-LI-LSN SGA -GSL-YV-S-DEFI--TGQHK

Amphibians PIP5Kα

Xenopus laevis-Alpha-5K	NP_001082731.1	EL---KPD---Y--CSE-LI-LSN PGA -GSI-FVTS-DEFI--T-QHK
Nanorana parkeri-Alpha-5K	XP_018421916.1	EL---RPD---Y--CNE-LF-LCN SGA -GSL-YV-G-DEFI--T-QHK
Xenopus tropicalis-Alpha-5K	NP_001006899.1	EL-S-RPD---Y--CNE-LI-LSN PGA -GSV-YV-G-DEFI--T-QHK

Reptiles PIP5Kα

Protobothrops mucrosquamatus-Alpha-5K	XP_015680374.1	EL---RPD---C--CSE-LI-LSN SGA -GSI-YV-S-DEFI--T-QHK
Python bivittatus-Alpha-5K	XP_015744819.1	EL---RPD---C--CSE-LI-LSN SGA -GSI-YV-S-DEFI--T-QHK
Gekko japonicus-Alpha-5K	XP_015279998.1	EL---RPD---C--CSE-LI-LSN SGA -GSI-YV-S-DEFI--T-QHK
Anolis carolinensis-Alpha-5K	XP_008120464.1	EL---RPD---C--CSE-LI-LSN SGA -GSI-YV-S-DEFI--T-QHK
Pogona vitticeps-Alpha-5K	XP_020653819.1	EL---RPD---C--CSE-LI-LSN SGA -GSI-YV-S-DEFI--T-QHK

Fishes PIP5Kα

Maylandia zebra-Alpha-5K	XP_004541373.2	EL---RPD---Y--CNE-LI-LSN PGA -GSL-YV-S-DEFI--T-QHK
Cynoglossus semilaevis-Alpha-5K	XP_008321275.1	EL---RPD---Y--CNE-LI-LSN PGA -GSL-YV-S-DEFI--T-QHK
Boleophthalmus pectinirostris-Alpha-5K	XP_020783608.1	EL---RPD---Y--CNE-LI-LSN PGA -GSL-YV-S-DEFI--T-QHK
Oreochromis niloticus-Alpha-5K	XP_005472748.1	EL---RPD---Y--CNE-LI-LSN PGA -GSL-YV-S-DEFI--T-QHK
Lates calcarifer-Alpha-5K	XP_018541718.1	EL---RPD---Y--CNE-LI-LSN PGA -GSL-YV-S-DEFI--T-QHK
Neolamprologus brichardi-Alpha-5K	XP_006804986.1	EL---RPD---Y--CNE-LI-LSN PGA -GSL-YV-S-DEFI--T-QHK
Pundamilia nyererei-Alpha-5K	XP_005732196.1	EL---RPD---Y--CNE-LI-LSN PGA -GSL-YV-S-DEFI--T-QHK
Stegastes partitus-Alpha-5K	XP_008285964.1	EL---RPD---Y--CNE-LI-LSN PGA -GSL-YV-S-DEFI--T-QHK
Acanthochromis polyacanthus-Alpha-5K	XP_022051707.1	EL---RPD---Y--CNE-LI-LSN PGA -GSL-YV-S-DEFI--T-QHK
Labrus bergylta-Alpha-5K	XP_020497624.1	EL---RPD---Y--CNE-LI-LSN PGA -GSL-YV-S-DEFI--T-QHK
Amphiprion ocellaris-Alpha-5K	XP_023117624.1	EL---RPD---Y--CNE-LI-LSN PGA -GSL-YV-S-DEFI--T-QHK
Monopterus albus-Alpha-5K	XP_020462945.1	EL---RPD---Y--CNE-LI-LSN PGA -GSL-YV-S-DEFI--T-QHK
Seriola dumerili-Alpha-5K	XP_022621881.1	EL---RPD---Y--CNE-LI-LSN PGA -GSL-YV-S-DEFI--T-QHK
Hippocampus comes-Alpha-5K	XP_019747686.1	EL---RPD---Y--CNE-LI-LSN PGA -GSL-YV-S-DEFI--T-QHK
Cyprinodon variegatus-Alpha-5K	XP_015248386.1	EM---RPD---Y--CNE-LI-LSN PGA -GSL-YV-S-DEFI--T-QHK
Fundulus heteroclitus-Alpha-5K	XP_012721150.1	EM---RPD---Y--CNE-LI-LSN PGA -GSL-YV-S-DEFI--T-QHK
Paralichthys olivaceus-Alpha-5K	XP_019948859.1	EL---RPD---Y--CNE-LI-LSN PGA -GSI-YV-S-DEFI--T-QHK
Poecilia mexicana-Alpha-5K	XP_014839941.1	EM---RPD---Y--CNE-LI-LSN PGA -GSI-YV-S-DEFI--T-QHK
Nothobranchius furzeri-Alpha-5K	XP_015806121.1	EM---RPD---Y--CNE-LI-LSN PGA -GSI-YV-S-DEFI--T-QHK
Kryptolebias marmoratus-Alpha-5K	XP_017280565.1	EM---RPD---Y--CNE-LI-LSN PGA -GSI-YV-S-DEFI--T-QHK
Tetraodon nigroviridis-Alpha-5K	CAG11890.1	EL---RPD---Y--CNE-LI-LSN PGA -GSI-YV-S-DEFI--T-QHK
Xiphophorus maculatus-Alpha-5K	XP_023186755.1	EM---RPD---Y--CNE-LI-LSN PGA -GSI-YV-S-DEFI--T-QHK
Austrofundulus limnaeus-Alpha-5K	XP_013885096.1	EM---RPD---Y--CNE-LI-LSN PGA -GSI-YV-S-DEFI--T-QHK
Oryzias latipes-Alpha-5K	XP_004078182.1	EM---RPD--MY--CNE-LI-LSN PGA -GSI-YV-S-DEFI--T-QHK
Pygocentrus nattereri-Alpha-5K	XP_017555944.1	EL---RPD---Y--CND-LI-LSN PGA -GSI-YV-S-DEFI--T-QHK
Clupea harengus-Alpha-5K	XP_012684664.1	EL---RPD---Y--CNDALI-LSN PGA -GSI-YV-S-DEFI--T-QHK
Takifugu rubripes-Alpha-5K	XP_011603926.1	EL---RPD---Y--CNERLI-LSN PGA -GSI-YV-S-DEFI--T-QHK
Astyanax mexicanus-Alpha-5K	XP_022529206.1	EL---RPD---Y--CND-LI-LSN SGA -GSI-YV-S-DEFI--T-QHK

Salvelinus alpinus-Alpha-5K	XP_024001797.1	EL---RPD---Y--CNDGLI-LSN SGA -GSL-YV-S-DEFI--T-QHK
Oncorhynchus kisutch-Alpha-5K	XP_020358970.1	EL---RPD---Y--CNDGLI-LSN SGA -GSL-YV-S-DEFI--T-QHK
Salmo salar-Alpha-5K	XP_014057413.1	EL---RPD---Y--CNDGLI-LSN SGA -GSL-YV-S-DEFI--T-QHK
Esox lucius-Alpha-5K	XP_012994913.1	EL---RPD---Y--CNEDLI-LSN SGA -GSV-YV-S-DEFI--T-QHK

Mammals PIP5Kβ

Pan troglodytes-Beta-5K	PNI61986.1	EL---KPD---Y-ICSE-LI-LSN PGA -GSL-FVTS-DEFI--T-QHK
Pongo abelii-Beta-5K	PNJ81439.1	EL---KPD---Y-ICSE-LI-LSN PGA -GSL-FVTS-DEFI--T-QHK
Carlito syrichta-Beta-5K	XP_008064264.1	EL---KPD---Y-ICSE-LI-LSN PGA -GSL-FVTS-DEFI--T-QHK
Heterocephalus glaber-Beta-5K	XP_021108403.1	EL---KPD---Y-ICSE-LI-LSN PGA -GSL-FVTS-DEFI--T-QHK
Leptonychotes weddellii-Beta-5K	XP_006748303.1	EL---KPD---Y-ICSE-LI-LSN PGA -GSL-FVTS-DEFI--T-QHK
Ictidomys tridecemlineatus-Beta-5K	XP_021581313.1	EL---KPD---Y-ICSE-LI-LSN PGA -GSL-FVTS-DEFI--T-QHK
Ailuropoda melanoleuca-Beta-5K	XP_019664190.1	EL---KPD---Y-ICSE-LI-LSN PGA -GSL-FVTS-DEFI--T-QHK
Fukomys damarensis-Beta-5K	KF022886.1	EL---KPD---Y-ICSE-LI-LSN PGA -GSL-FVTS-DEFI--T-QHK
Chinchilla lanigera-Beta-5K	XP_013371800.1	EL---KPD---Y-ICSE-LI-LSN PGA -GSL-FVTS-DEFI--T-QHK
Equus przewalskii-Beta-5K	XP_008510987.1	EL---KPD---Y-ICSE-LI-LSN PGA -GSL-FVTS-DEFI--T-QHK
Manis javanica-Beta-5K	XP_017509139.1	EL---KPD---Y-ICSE-LI-LSN PGA -GSL-FVTS-DEFI--T-QHK
Lipotes vexillifer-Beta-5K	XP_007466832.1	EL---KPD---Y-ICSE-LI-LSN PGA -GSL-FVTS-DEFI--T-QHK
Nomascus leucogenys-Beta-5K	XP_003273915.1	EL---KPD---Y-ICSE-LI-LSN PGA -GSL-FVTS-DEFI--T-QHK
Delphinapterus leucas-Beta-5K	XP_022429264.1	EL---KPD---Y-ICSE-LI-LSN PGA -GSL-FVTS-DEFI--T-QHK
Homo sapiens-Beta-5K	EAW62466.1	EL---KPD---Y-ICSE-LI-LSN PGA -GSL-FVTS-DEFI--T-QHK
Odobenus rosmarus divergens-Beta-5K	XP_004397583.1	EL---KPD---Y-ICSE-LI-LSN PGA -GSL-FVTS-DEFI--T-QHK
Orcinus orca-Beta-5K	XP_004276469.1	EL---KPD---Y-ICSE-LI-LSN PGA -GSL-FVTS-DEFI--T-QHK
Elephantulus edwardii-Beta-5K	XP_006902442.1	EL---KPD---Y-ICSE-LI-LSN PGA -GSL-FVTS-DEFI--T-QHK
Panthera tigris altaica-Beta-5K	XP_007077533.2	EL---KPD---Y-ICSE-LI-LSN PGA -GSL-FVTS-DEFI--T-QHK
Acinonyx jubatus-Beta-5K	XP_014919991.1	EL---KPD---Y-ICSE-LI-LSN PGA -GSL-FVTS-DEFI--T-QHK
Gorilla gorilla gorilla-Beta-5K	XP_018889556.1	EL---KPD---Y-ICSE-LI-LSN PGA -GSL-FVTS-DEFI--T-QHK
Physeter catodon-Beta-5K	XP_023982630.1	EL---KPD---Y-ICSE-LI-LSN PGA -GSL-FVTS-DEFI--T-QHK
Balaenoptera acutorostrata sca-Beta-5K	XP_007182303.1	EL---KPD---Y-ICSE-LI-LSN PGA -GSL-FVTS-DEFI--T-QHK
Loxodonta africana-Beta-5K	XP_023395212.1	EL---KPD---Y-ICSE-LI-LSN PGA -GSL-FVTS-DEFI--T-QHK
Mandrillus leucophaeus-Beta-5K	XP_011825535.1	EL---KPD---Y-ICSE-LI-LSN PGA -GSL-FVTS-DEFI--T-QHK
Phascolarctos cinereus-Beta-5K	XP_020857461.1	EL---KPD---Y-ICSE-LI-LSN PGA -GSL-FVTS-DEFI--T-QHK
Aotus nancymaae-Beta-5K	XP_012309035.1	EL---KPD---Y-ICSE-LI-LSN PGA -GSL-FVTS-DEFI--T-QHK
Monodelphis domestica-Beta-5K	XP_007498641.1	EL---KPD---Y-ICSE-LI-LSN PGA -GSL-FVTS-DEFI--T-QHK
Equus caballus-Beta-5K	XP_023483021.1	EL---KPD---Y-ICSE-LI-LSN PGA -GSL-FVTS-DEFI--T-QHK
Pan paniscus-Beta-5K	XP_003824805.1	EL---KPD---Y-ICSE-LI-LSN PGA -GSL-FVTS-DEFI--T-QHK
Panthera pardus-Beta-5K	XP_019319198.1	EL---KPD---Y-ICSE-LI-LSN PGA -GSL-FVTS-DEFI--T-QHK
Colobus angolensis palliatus-Beta-5K	XP_011804275.1	EL---KPD---Y-ICSE-LI-LSN PGA -GSL-FVTS-DEFI--T-QHK
Hipposideros armiger-Beta-5K	XP_019516639.1	EL---KPD---Y-ICSE-LI-LSN PGA -GSL-FVTS-DEFI--T-QHK
Equus asinus-Beta-5K	XP_014702264.1	EL---KPD---Y-ICSE-LI-LSN PGA -GSL-FVTS-DEFI--T-QHK
Sus scrofa-Beta-5K	XP_020920288.1	EL---KPD---Y-ICSE-LI-LSN PGA -GSL-FVTS-DEFI--T-QHK
Tupaia chinensis-Beta-5K	XP_006152528.1	EL---KPD---Y-ICSE-LI-LSN PGA -GSL-FVTS-DEFI--T-QHK
Pteropus alecto-Beta-5K	XP_015450016.1	EL---KPD---Y-ICSE-LI-LSN PGA -GSL-FVTS-DEFI--T-QHK
Octodon degus-Beta-5K	XP_023577654.1	EL---KPD---Y-ICSE-LI-LSN PGA -GSL-FVTS-DEFI--T-QHK
Cercocebus atys-Beta-5K	XP_011912030.1	EL---KPD---Y-ICSE-LI-LSN PGA -GSL-FVTS-DEFI--T-QHK
Papio anubis-Beta-5K	XP_003911826.1	EL---KPD---Y-ICSE-LI-LSN PGA -GSL-FVTS-DEFI--T-QHK
Dipodomys ordii-Beta-5K	XP_012873938.1	EL---KPD---Y-ICSE-LI-LSN PGA -GSL-FVTS-DEFI--T-QHK
Tursiops truncatus-Beta-5K	XP_019790298.1	EL---KPD---Y-ICSE-LI-LSN PGA -GSL-FVTS-DEFI--T-QHK
Rhinolophus sinicus-Beta-5K	XP_019585928.1	EL---KPD---Y-ICSE-LI-LSN PGA -GSL-FVTS-DEFI--T-QHK
Galeopterus variegatus-Beta-5K	XP_008578885.1	EL---KPD---Y-ICSE-LI-LSN PGA -GSL-FVTS-DEFI--T-QHK
Erinaceus europaeus-Beta-5K	XP_016046043.1	EL---KPD---Y-ICSE-LI-LSN PGA -GSL-FVTS-DEFI--T-QHK
Rousettus aegyptiacus-Beta-5K	XP_015991032.1	EL---KPD---Y-ICSE-LI-LSN PGA -GSL-FVTS-DEFI--T-QHK
Microcebus murinus-Beta-5K	XP_012592396.1	EL---KPD---Y-ICSE-LI-LSN PGA -GSL-FVTS-DEFI--T-QHK
Bos indicus-Beta-5K	XP_019821104.1	EL---KPD---Y-ICSE-LI-LSN PGA -GSL-FVTS-DEFI--T-QHK
Rhinopithecus roxellana-Beta-5K	XP_010361273.1	EL---KPD---Y-ICSE-LI-LSN PGA -GSL-FVTS-DEFI--T-QHK
Bos mutus-Beta-5K	XP_005900082.1	EL---KPD---Y-ICSE-LI-LSN PGA -GSL-FVTS-DEFI--T-QHK
Canis lupus familiaris-Beta-5K	XP_013973148.1	EL---KPD---Y-ICSE-LI-LSN PGA -GSL-FVTS-DEFI--T-QHK
Odocoileus virginianus texanus-Beta-5K	XP_020767573.1	EL---KPD---Y-ICSE-LI-LSN PGA -GSL-FVTS-DEFI--T-QHK
Ceratotherium simum simum-Beta-5K	XP_004440696.1	EL---KPD---Y-ICSE-LI-LSN PGA -GSL-FVTS-DEFI--T-QHK
Pantholops hodgsonii-Beta-5K	XP_005976403.1	EL---KPD---Y-ICSE-LI-LSN PGA -GSL-FVTS-DEFI--T-QHK
Cavia porcellus-Beta-5K	XP_003472322.2	EL---KPD---Y-ICSE-LI-LSN PGA -GSL-FVTS-DEFI--T-QHK
Chlorocebus sabaeus-Beta-5K	XP_007967618.1	EL---KPD---Y-ICSE-LI-LSN PGA -GSL-FVTS-DEFI--T-QHK
Cebus capucinus imitator-Beta-5K	XP_017398912.1	EL---KPD---Y-ICSE-LI-LSN PGA -GSL-FVTS-DEFI--T-QHK
Bison bison bison-Beta-5K	XP_010858968.1	EL---KPD---Y-ICSE-LI-LSN PGA -GSL-FVTS-DEFI--T-QHK
Propithecus coquereli-Beta-5K	XP_012497079.1	EL---KPD---Y-ICSE-LI-LSN PGA -GSL-FVTS-DEFI--T-QHK
Mustela putorius furo-Beta-5K	XP_004769301.2	EL---KPD---Y-ICSE-LI-LSN PGA -GSL-FVTS-DEFI--T-QHK

Birds PIP5Kβ

Bambusicola thoracicus-Beta-5K	POI34043.1	EL---KPD---Y-ICSE-LI-LSN PGA -GSL-FVTS-DEFI--T-QHK
Apaloderma vittatum-Beta-5K	KFP74394.1	EL---KPD---Y-ICSE-LI-LSN PGA -GSL-FVTS-DEFI--T-QHK
Colius striatus-Beta-5K	KFP25871.1	EL---KPD---Y-ICSE-LI-LSN PGA -GSL-FVTS-DEFI--T-QHK
Eurypyga helias-Beta-5K	KFV95544.1	EL---KPD---Y-ICSE-LI-LSN PGA -GSL-FVTS-DEFI--T-QHK
Tyto alba-Beta-5K	KFV58308.1	EL---KPD---Y-ICSE-LI-LSN PGA -GSL-FVTS-DEFI--T-QHK
Phoenicopterus ruber ruber-Beta-5K	KFQ88387.1	EL---KPD---Y-ICSE-LI-LSN PGA -GSL-FVTS-DEFI--T-QHK
Acanthisitta chloris-Beta-5K	XP_009075316.1	EL---KPD---Y-ICSE-LI-LSN PGA -GSL-FVTS-DEFI--T-QHK
Fulmarus glacialis-Beta-5K	XP_009574388.1	EL---KPD---Y-ICSE-LI-LSN PGA -GSL-FVTS-DEFI--T-QHK
Manacus vitellinus-Beta-5K	XP_017936401.1	EL---KPD---Y-ICSE-LI-LSN PGA -GSL-FVTR-DEFI--T-QHK

Numida meleagris-Beta-5K	XP_021236396.1	EL---KPD---Y-ICSE-LI-LSN PGA -GSL-FVTS-DEFI--T-QHK
Gallus gallus-Beta-5K	NP_001026593.1	EL---KPD---Y-ICSE-LI-LSN PGA -GSL-FVTS-DEFI--T-QHK
Chlamydotis macqueenii-Beta-5K	KFP44763.1	EL---KPD---Y-ICSE-LI-LSN PGA -GSL-FVTS-DEFI--T-QHK
Tinamus guttatus-Beta-5K	XP_010224625.1	EL---KPD---Y-ICSE-LI-LSN PGA -GSL-FVTS-DEFI--T-QHK
Phaethon lepturus-Beta-5K	KFQ71669.1	EL---KPD---Y-ICSE-LI-LSN PGA -GSL-FVTS-DEFI--T-QHK
Struthio camelus australis-Beta-5K	XP_009670362.1	EL---KPD---Y-ICSE-LI-LSN PGA -GSL-FVTS-DEFI--T-QHK
Anas platyrhynchos-Beta-5K	EOB05878.1	EL---KPD---Y-ICSE-LI-LSN PGA -GSL-FVTS-DEFI--T-QHK
Calidris pugnax-Beta-5K	XP_014807148.1	EL---KPD---Y-ICSE-LI-LSN PGA -GSL-FVTS-DEFI--T-QHK
Anser cygnoides domesticus-Beta-5K	XP_013049494.1	EL---KPD---Y-ICSE-LI-LSN PGA -GSL-FVTS-DEFI--T-QHK
Aquila chrysaetos canadensis-Beta-5K	XP_011576030.1	EL---KPD---Y-ICSE-LI-LSN PGA -GSL-FVTS-DEFI--T-QHK
Pygoscelis adeliae-Beta-5K	XP_009326352.1	EL---KPD---Y-ICSE-LI-LSN PGA -GSL-FVTS-DEFI--T-QHK
Tauraco erythrolophus-Beta-5K	KFV06911.1	EL---KPD---Y-ICSE-LI-LSN PGA -GSL-FVTS-DEFI--T-QHK
Falco peregrinus-Beta-5K	XP_013158485.1	EL---KPD---Y-ICSE-LI-LSN PGA -GSL-FVTS-DEFI--T-QHK
Merops nubicus-Beta-5K	XP_008938298.1	EL---KPD---Y-ICSE-LI-LSN PGA -GSL-FVTS-DEFI--T-QHK
Antrostomus carolinensis-Beta-5K	XP_010161257.1	EL---KPD---Y-ICSE-LI-LSN PGA -GSL-FVTS-DEFI--T-QHK
Aptenodytes forsteri-Beta-5K	KFM11626.1	EL---KPD---Y-ICSE-LI-LSN PGA -GSL-FVTS-DEFI--T-QHK
Haliaeetus leucocephalus-Beta-5K	XP_010566885.1	EL---KPD---Y-ICSE-LI-LSN PGA -GSL-FVTS-DEFI--T-QHK
Buceros rhinoceros silvestris-Beta-5K	XP_010138959.1	EL---KPD---Y-ICSE-LI-LSN PGA -GSL-FVTS-DEFI--T-QHK
Mesitornis unicolor-Beta-5K	KFQ36696.1	EL---KPD---Y-ICSE-LI-LSN PGA -GSL-FVTS-DEFI--T-QHK
Coturnix japonica-Beta-5K	XP_015704586.1	EL---KPD---Y-ICSE-LI-LSN PGA -GSL-FVTS-DEFI--T-QHK
Egretta garzetta-Beta-5K	XP_009634406.1	EL---KPD---Y-ICSE-LI-LSN PGA -GSL-FVTS-DEFI--T-QHK
Nipponia nippon-Beta-5K	XP_009458847.1	EL---KPD---Y-ICSE-LI-LSN PGA -GSL-FVTS-DEFI--T-QHK
Phalacrocorax carbo-Beta-5K	KFW89852.1	EL---KPG---Y-ICSE-LI-LSN PGA -GSL-FVTS-DEFI--T-QHK
Columba livia-Beta-5K	XP_005515418.1	EL---KPD---Y-ICSE-LI-LSN PGA -GSL-FVTS-DEFI--T-QHK
Limosa lapponica baueri-Beta-5K	PKU45027.1	EL---KPD---Y-ICSE-LI-LSN PGA -GSL-FVTS-DEFI--T-QHK
Callipepla squamata-Beta-5K	OXB62686.1	EL---KPD---Y-ICSE-LI-LSN PGA -GSL-FVTS-DEFI--T-QHK
Opisthocomus hoazin-Beta-5K	XP_009937189.1	EL---KPD---Y-ICSE-LI-LSN PGA -GSL-FVTS-DEFI--T-QHK
Gavia stellata-Beta-5K	XP_009806849.1	EL---KPD---Y-ICNE-LI-LSN PGA -GSL-FVTS-DEFI--T-QHK
Nestor notabilis-Beta-5K	XP_010015056.1	EL---KPD---Y--CSE-LI-LSN PGA -GSL-FVTS-DEFI--T-QHK
Chaetura pelagica-Beta-5K	XP_010006792.1	EL---KPD---Y-VCSE-LI-LSN PGA -GSL-FVTS-DEFI--T-QHK
Amazona aestiva-Beta-5K	KQK73386.1	EL---KPD---Y--CSE-LI-LSN PGA -GSL-FVTS-DEFI--T-QHK
Cariama cristata-Beta-5K	KFP66862.1	EL---KPD---Y-ICSE-LI-LSN PGA -GSL-FVTG-DEFI--T-QHK
Melopsittacus undulatus-Beta-5K	XP_005155024.1	EL---KPD---Y--CSE-LI-LSN PGA -GSL-FVTS-DEFI--T-QHK
Lepidothrix coronata-Beta-5K	XP_017661573.1	EL---KPD---Y-ICSE-LI-LSN PGA -GSL-FVTR-DEFI--T-QHK
Picoides pubescens-Beta-5K	KFV72101.1	EL---KPD---Y-ICSD-LI-LSN PGA -GSL-FVTS-DEFI--T-QHK
Pelecanus crispus-Beta-5K	XP_009480865.1	EL---KPG---Y-ICSE-LI-LSN PGA -GSL-FVTS-DEFI--T-QHK
Patagioenas fasciata monilis-Beta-5K	OPJ83314.1	EL---KPD---Y-ICSE-LI-LCN PGA -GSL-FVTS-DEFI--T-QHK
Balearica regulorum gibbericep-Beta-5K	KFO12738.1	EL---KAD---Y-ICSE-LI-LSN PGA -GSL-FVTS-DEFI--T-QHK
Corvus brachyrhynchos-Beta-5K	XP_017586099.1	EL---KPD---Y-ICSE-LI-LSN PGA -GSL-FVTSGDEFI--T-QHK
Lonchura striata domestica-Beta-5K	XP_021386187.1	EL---KPD---Y-ICSE-LI-LSN PGA -GSL-FVTSGDEFI--T-QHK
Charadrius vociferus-Beta-5K	XP_009886015.1	EL---KPG---Y-ICSE-LI-LSN PGA -GSL-FVTS-DEFI--T-QHK

Amphibians PIP5Kβ

Xenopus laevis-Beta-5K	NP_001082731.1	EL---KPD---Y--CSE-LI-LSN PGA -GSI-FVTS-DEFI--T-QHK
Xenopus tropicalis-Beta-5K	XP_012827258.1	EL---KPD---Y--CSE-LI-LSN PGA -GSI-FVTS-DEFI--T-QHK

Reptiles PIP5Kβ

Anolis carolinensis-Beta-5K	XP_008101520.1	EL---KPD---Y-ICSE-LI-LSN PGA -GSL-FVTS-DEFI--T-QHK
Gekko japonicus-Beta-5K	XP_015261333.1	EL---KPD---Y-ICSE-LI-LSN PGA -GSL-FVTS-DEFI--T-QHK
Pogona vitticeps-Beta-5K	XP_020661323.1	EL---KPD---Y-ICSE-LI-LSN PGA -GSL-FVTS-DEFI--T-QHK
Thamnophis sirtalis-Beta-5K	XP_013907811.1	EL---KPD---Y-ICSE-LI-LSN PGA -GSL-FVTS-DEFI--T-QHK
Ophiophagus hannah-Beta-5K	ETE59205.1	EL---KPD---Y-ICSE-LI-LSN PGA -GSL-FVTS-DEFI--T-QHK
Protobothrops mucrosquamatus-Beta-5K	XP_015679138.1	EL---KPD---Y-ICSE-LI-LSN PGA -GSL-FVTS-DEFI--T-QHK

Fishes PIP5Kβ

Danio rerio-Beta-5K	NP_001004579.1	EL---KPD---Y-ICKE-LI-LSN PGA -GSL-YLTS-DEFI--T-QHK
Seriola lalandi dorsalis-Beta-5K	XP_023255674.1	EL---KPD---Y-ICNE-LI-LSN PGA -SSW-YLTS-DEFI--T-QHK
Notothenia coriiceps-Beta-5K	XP_010787650.1	EL---KPD---Y-ICNE-LI-LSN PGA -SSW-YLTS-DEFI--T-QHK
Stegastes partitus-Beta-5K	XP_008274617.1	EL---KPD---Y-ICNE-LI-LSN PGA -SSW-YLTS-DEFI--T-QHK
Amphiprion ocellaris-Beta-5K	XP_023138083.1	EL---KPD---Y-ICNE-LI-LSN PGA -SSW-YLTS-DEFI--T-QHK
Lates calcarifer-Beta-5K	XP_018558033.1	EL---KPD---Y-ICNE-LI-LSN PGA -SSW-YLTS-DEFI--T-QHK
Acanthochromis polyacanthus-Beta-5K	XP_022072780.1	EL---KPD---Y-ICNE-LI-LSN PGA -SSW-YLTS-DEFI--T-QHK
Fundulus heteroclitus-Beta-5K	XP_021179699.1	EL---KPD---Y-ICKE-LI-LSN PGA -SSW-YLTS-DEFI--T-QHK
Cyprinodon variegatus-Beta-5K	XP_015228657.1	EL---KPD---Y-ICKE-LI-LSN PGA -SSW-YLTS-DEFI--T-QHK
Monopterus albus-Beta-5K	XP_020470796.1	EL---KPD---Y-ICKE-LI-LSN PGA -SSW-YLTS-DEFI--T-QHK
Poecilia formosa-Beta-5K	XP_007575909.1	EL---KPD---Y-ICKE-LI-LSN PGA -SSW-YLTS-DEFI--T-QHK
Oryzias latipes-Beta-5K	XP_011480348.1	EL---KPD---Y-MCNE-LI-LSN PGA -SSW-YLTS-DEFI--T-QHK
Xiphophorus maculatus-Beta-5K	XP_023193673.1	EL---KPD---Y-ICKE-LI-LSN PGA -SSW-YLTS-DEFI--T-QHK
Labrus bergylta-Beta-5K	XP_020491677.1	EL---KPD---Y-ICNE-LI-LTN PGA -SSW-YLTS-DEFI--T-QHK
Cynoglossus semilaevis-Beta-5K	XP_016894343.1	EL---KPD---Y-ICKE-LI-LTN PGA -SSW-YLTS-DEFI--T-QHK
Paralichthys olivaceus-Beta-5K	XP_019960688.1	EL---KPD---Y-ICNE-LI-LTN PGA -SSW-YLTS-DEFI--T-QHK
Oryzias melastigma-Beta-5K	XP_024154386.1	EL---KPD---Y-MCNE-LI-LSN PGA -SSW-YLTS-DEFI--T-QHK
Kryptolebias marmoratus-Beta-5K	XP_017288436.1	EL---KPD---Y-FCNE-LI-LTN PGA -SSW-YLTS-DEFI--T-QHK
Maylandia zebra-Beta-5K	XP_004538536.1	EL---KPD---Y-ICNE-LI-LSN PGA -SSW-YLTS-DEFI--T-QPK
Oreochromis niloticus-Beta-5K	XP_003440259.2	EL---KPD---Y-ICNE-LI-LSN PGA -SSW-YLTS-DEFI--T-QPK
Pundamilia nyererei-Beta-5K	XP_005725614.1	EL---KPD---Y-ICNE-LI-LSN PGA -SSW-YLTS-DEFI--T-QPK
Haplochromis burtoni-Beta-5K	XP_005921766.1	EL---KPD---Y-ICNE-LI-LSN PGA -SSW-YLTS-DEFI--T-QPK

Neolamprologus brichardi-Beta-5K	XP_006803535.1	EL---KPD---Y-ICNE-LI-LSN PGA -SSW-YLTS-DEFI--T-QPK
Larimichthys crocea-Beta-5K	XP_010734153.1	EL---KPD---Y-ICNE-LI-LSN PGA -SSW-YLTS-DEFI--T-QPK
Austrofundulus limnaeus-Beta-5K	XP_013878032.1	EL---KPD---Y-FCNE-LI-LSN PGA -SSW-YLTS-DEFI--T-QPK

Mammals PIP5Kγ

Pteropus vampyrus-Gamma-5K	XP_023382396.1	EL---RPD---Y--CNE-LI-LSN PGA -GSL-YVTS-DEFI--T-MHK
Homo sapiens-Gamma-5K	BAH14283.1	EL---RPD---Y--CNE-LI-LSN PGA -GSL-YVTS-DEFI--T-MHK
Cricetulus griseus-Gamma-5K	ERE72321.1	EL---RPD---Y--CNE-LI-LSN PGA -GSV-YVTS-DEFI--T-MHK
Microtus ochrogaster-Gamma-5K	XP_013206152.1	EL---RPD---Y--CNE-LI-LSN PGA -GSV-YVTS-DEFI--T-MHK
Rhinopithecus bieti-Gamma-5K	XP_017702716.1	EL---RPD---Y--CNE-LI-LSN PGA -GSL-YVTS-DEFI--T-MHK
Rattus norvegicus-Gamma-5K	Q5I6B8.1	EL---RPD---Y--CNE-LI-LSN PGA -GSV-YVTS-DEFI--T-MHK
Mus pahari-Gamma-5K	XP_021060104.1	EL---RPD---Y--CNE-LI-LSN PGA -GSV-YVTS-DEFI--T-MHK
Mus caroli-Gamma-5K	XP_021029451.1	EL---RPD---Y--CNE-LI-LSN PGA -GSV-YVTS-DEFI--T-MHK
Mus musculus-Gamma-5K	NP_001280575.1	EL---RPD---Y--CNE-LI-LSN PGA -GSV-YVTS-DEFI--T-MHK
Equus asinus-Gamma-5K	XP_014698530.1	EL---RPD---Y--CNE-LI-LSN PGA -GSL-YVTS-DEFI--T-MHK
Meriones unguiculatus-Gamma-5K	XP_021492517.1	EL---RPD---Y--CNE-LI-LSN PGA -GSV-YVTS-DEFI--T-MHK
Mesocricetus auratus-Gamma-5K	XP_021090570.1	EL---RPD---Y--CNE-LI-LSN PGA -GSV-YVTS-DEFI--T-MHK
Peromyscus maniculatus bairdii-Gamma-5K	XP_015852362.1	EL---RPD---Y--CNE-LI-LSN PGA -GSV-YVTS-DEFI--T-MHK
Nomascus leucogenys-Gamma-5K	XP_012358835.1	EL---RPD---Y--CNE-LI-LSN PGA -GSL-YVTS-DEFI--T-MHK
Marmota marmota marmota-Gamma-5K	XP_015359305.1	EL---RPD---Y--CNE-LI-LSN PGA -GSL-YVTS-DEFI--T-MHK
Microcebus murinus-Gamma-5K	XP_012624768.1	EL---RPD---Y--CNE-LI-LSN PGA -GSL-YVTS-DEFI--T-MHK
Ictidomys tridecemlineatus-Gamma-5K	XP_021586552.1	EL---RPD---Y--CNE-LI-LSN PGA -GSL-YVTS-DEFI--T-MHK
Bison bison bison-Gamma-5K	XP_010826423.1	EL---RPD---Y--CNE-LI-LSN PGA -GSL-YVTS-DEFI--T-MHK
Dipodomys ordii-Gamma-5K	XP_012878297.1	EL---RPD---Y--CNE-LI-LSN PGA -GSL-YVTS-DEFI--T-MHK
Carlito syrichta-Gamma-5K	XP_021564757.1	EL---RPD---Y--CNE-LI-LSN PGA -GSL-YVTS-DEFI--T-MHK
Panthera pardus-Gamma-5K	XP_019286318.1	EL---RPD---Y--CNE-LI-LSN PGA -GSL-YVTS-DEFI--T-MHK
Cercocebus atys-Gamma-5K	XP_011928453.1	EL---RPD---Y--CNE-LI-LSN PGA -GSL-YVTS-DEFI--T-MHK
Ovis aries musimon-Gamma-5K	XP_011994873.1	EL---RPD---Y--CNE-LI-LSN PGA -GSL-YVTS-DEFI--T-MHK
Delphinapterus leucas-Gamma-5K	XP_022412663.1	EL---RPD---Y--CNE-LI-LSN PGA -GSL-YVTS-DEFI--T-MHK
Macaca fascicularis-Gamma-5K	XP_005587579.1	EL---RPD---Y--CNE-LI-LSN PGA -GSL-YVTS-DEFI--T-MHK
Octodon degus-Gamma-5K	XP_023572647.1	EL---RPD---Y--CNE-LI-LSN PGA -GSL-YVTS-DEFI--T-MHK
Bos indicus-Gamma-5K	XP_019820038.1	EL---RPD---Y--CNE-LI-LSN PGA -GSL-YVTS-DEFI--T-MHK
Felis catus-Gamma-5K	XP_023099540.1	EL---RPD---Y--CNE-LI-LSN PGA -GSL-YVTS-DEFI--T-MHK
Papio anubis-Gamma-5K	XP_009191416.1	EL---RPD---Y--CNE-LI-LSN PGA -GSL-YVTS-DEFI--T-MHK
Condylura cristata-Gamma-5K	XP_012585106.1	EL---RPD---Y--CNE-LI-LSN PGA -GSL-YVTS-DEFI--T-MHK
Vicugna pacos-Gamma-5K	XP_015096640.1	EL---RPD---Y--CNE-LI-LSN PGA -GSL-YVTS-DEFI--T-MHK
Odobenus rosmarus divergens-Gamma-5K	XP_004395402.1	EL---RPD---Y--CNE-LI-LSN PGA -GSL-YVTS-DEFI--T-MHK
Capra hircus-Gamma-5K	XP_017906326.1	EL---RPD---Y--CNE-LI-LSN PGA -GSL-YVTS-DEFI--T-MHK
Cavia porcellus-Gamma-5K	XP_013000232.1	EL---RPD---Y--CNE-LI-LSN PGA -GSL-YVTS-DEFI--T-MHK
Piliocolobus tephrosceles-Gamma-5K	XP_023039376.1	EL---RPD---Y--CNE-LI-LSN PGA -GSL-YVTS-DEFI--T-MHK
Rhinopithecus roxellana-Gamma-5K	XP_010365584.1	EL---RPD---Y--CNE-LI-LSN PGA -GSL-YVTS-DEFI--T-MHK
Otolemur garnettii-Gamma-5K	XP_012659946.2	EL---RPD---Y--CNE-LI-LSN PGA -GSL-YVTS-DEFI--T-MHK
Equus caballus-Gamma-5K	XP_023500173.1	EL---RPD---Y--CNE-LI-LSN PGA -GSL-YVTS-DEFI--T-MHK
Ursus maritimus-Gamma-5K	XP_008709629.1	EL---RPD---Y--CNE-LI-LSN PGA -GSL-YVTS-DEFI--T-MHK
Bubalus bubalis-Gamma-5K	XP_006047953.1	EL---RPD---Y--CNE-LI-LSN PGA -GSL-YVTS-DEFI--T-MHK
Sus scrofa-Gamma-5K	XP_020939762.1	EL---RPD---Y--CNE-LI-LSN PGA -GSL-YVTS-DEFI--T-MHK
Cebus capucinus imitator-Gamma-5K	XP_017362897.1	EL---RPD---Y--CNE-LI-LSN PGA -GSL-YVTS-DEFI--T-MHK
Rhinolophus sinicus-Gamma-5K	XP_019599697.1	EL---RPD---Y--CNE-LI-LSN PGA -GSL-YVTS-DEFI--T-MHK
Miniopterus natalensis-Gamma-5K	XP_016053317.1	EL---RPD---Y--CNE-LI-LSN PGA -GSL-YVTS-DEFI--T-MHK
Myotis lucifugus-Gamma-5K	XP_006105997.1	EL---RPD---Y--CNE-LI-LSN PGA -GSL-YVTS-DEFI--T-MHK
Castor canadensis-Gamma-5K	XP_020036848.1	EL---RPD---Y--CNE-LI-LSN PGA -GSL-YVTS-DEFI--T-MHK
Gorilla gorilla gorilla-Gamma-5K	XP_018871823.1	EL---RPD---Y--CNE-LI-LSN PGA -GSL-YVTS-DEFI--T-MHK
Bos taurus-Gamma-5K	XP_015327551.1	EL---RPD---Y--CNE-LI-LSN PGA -GSL-YVTS-DEFI--T-MHK
Pan troglodytes-Gamma-5K	XP_016792552.1	EL---RPD---Y--CNE-LI-LSN PGA -GSL-YVTS-DEFI--T-MHK
Nannospalax galili-Gamma-5K	XP_008835270.1	EL---RPD---Y--CNE-LI-LSN PGA -GSL-YVTS-DEFI--T-MHK
Odocoileus virginianus texanus-Gamma-5K	XP_020751005.1	EL---RPD---Y--CNE-LI-LSN PGA -GSL-YVTS-DEFI--T-MHK
Manis javanica-Gamma-5K	XP_017509454.1	EL---RPD---Y--CNE-LI-LSN PGA -GSL-YVTS-DEFI--T-MHK
Dasypus novemcinctus-Gamma-5K	XP_012380544.1	EL---RPD---Y--CNE-LI-LSN PGA -GSL-YVTS-DEFI--T-MHK

Birds PIP5K γ

Opisthocomus hoazin-Gamma-5K	XP_009940139.1	EL---RPD---Y--CNE-LI-LSN PGA -GSL-YVTSNDEFI--T-MHK
Lepidothrix coronata-Gamma-5K	XP_017692513.1	EL---RPD---Y--CNE-LI-LSN PGA -GSL-YVTS-DEFI--T-MHK
Chlamydotis macqueenii-Gamma-5K	XP_010124972.1	EL---RPD---Y--CNE-LI-LSN PGA -GSL-YVTS-DEFI--T-MHK
Eurypyga helias-Gamma-5K	XP_010151218.1	EL---RPD---Y--CNE-LI-LSN PGA -GSL-YVTS-DEFI--T-MHK
Parus major-Gamma-5K	XP_015507018.1	EL---RPD---Y--CNE-LI-LSN PGA -GSL-YVTS-DEFI--T-MHK
Taeniopygia guttata-Gamma-5K	XP_012426629.1	EL---RPD---Y--CNE-LI-LSN PGA -GSL-YVTS-DEFI--T-MHK
Numida meleagris-Gamma-5K	XP_021234277.1	EL---RPD---Y--CNE-LI-LSN PGA -GSL-YVTS-DEFI--T-MHK
Colinus virginianus-Gamma-5K	OXB69719.1	EL---RPD---Y--CNE-LI-LSN PGA -GSL-YVTS-DEFI--T-MHK
Manacus vitellinus-Gamma-5K	XP_017939015.1	EL---RPD---Y--CNE-LI-LSN PGA -GSL-YVTS-DEFI--T-MHK
Serinus canaria-Gamma-5K	XP_018777310.1	EL---RPD---Y--CNE-LI-LSN PGA -GSL-YVTS-DEFI--T-MHK
Haliaeetus albicilla-Gamma-5K	XP_009926191.1	EL---RPD---Y--CNE-LI-LSN PGA -GSL-YVTS-DEFI--T-MHK
Pseudopodoces humilis-Gamma-5K	XP_014115569.1	EL---RPD---Y--CNE-LI-LSN PGA -GSL-YVTS-DEFI--T-MHK
Cuculus canorus-Gamma-5K	KF078532.1	EL---RPD---Y--CNE-LI-LSN PGA -GSL-YVTS-DEFI--T-MHK
Amazona aestiva-Gamma-5K	KQK82211.1	EL---RPD---Y--CNE-LI-LSN PGA -GSL-YVTS-DEFI--T-MHK
Columba livia-Gamma-5K	XP_021137288.1	EL---RPD---Y--CNE-LI-LSN PGA -GSL-YVTS-DEFI--T-MHK
Pygoscelis adeliae-Gamma-5K	XP_009321283.1	EL---RPD---Y--CNE-LI-LSN PGA -GSL-YVTS-DEFI--T-MHK
Sturnus vulgaris-Gamma-5K	XP_014739865.1	EL---RPD---Y--CNE-LI-LSN PGA -GSL-YVTS-DEFI--T-MHK

Ficedula albicollis-Gamma-5K	XP_016159970.1	EL---RPD---Y--CNE-LI-LSN PGA -GSL-YVTS-DEFI--T-MHK
Lonchura striata domestica-Gamma-5K	XP_021383894.1	EL---RPD---Y--CNE-LI-LSN PGA -GSL-YVTS-DEFI--T-MHK
Gallus gallus-Gamma-5K	NP_001305950.1	EL---RPD---Y--CNE-LI-LSN PGA -GSL-YVTS-DEFI--T-MHK
Calypte anna-Gamma-5K	XP_008496647.1	EL---RPD---Y--CNE-LI-LSN PGA -GSL-YVTS-DEFI--T-MHK
Cyanistes caeruleus-Gamma-5K	XP_023798643.1	EL---RPD---Y--CNE-LI-LSN PGA -GSL-YVTS-DEFI--T-MHK
Podiceps cristatus-Gamma-5K	KFZ64041.1	EL---RPD---Y--CNE-LI-LSN PGA -GSL-YVTS-DEFI--T-MHK
Falco cherrug-Gamma-5K	XP_014143069.1	EL---RPD---Y--CNE-LI-LSN PGA -GSL-YVTS-DEFI--T-MHK
Gavia stellata-Gamma-5K	KFV49688.1	EL---RPD---Y--CNE-LI-LSN PGA -GSL-YVTS-DEFI--T-MHK
Mesitornis unicolor-Gamma-5K	KFQ21874.1	EL---RPD---Y--CNE-LI-LSN PGA -GSL-YVTS-DEFI--T-MHK
Aptenodytes forsteri-Gamma-5K	KFM06020.1	EL---RPD---Y--CNE-LI-LSN PGA -GSL-YVTS-DEFI--T-MHK
Aquila chrysaetos canadensis-Gamma-5K	XP_011591930.1	EL---RPD---Y--CNE-LI-LSN PGA -GSL-YVTS-DEFI--T-MHK
Apteryx australis mantelli-Gamma-5K	XP_013798587.1	EL---RPD---Y--CNE-LI-LSN PGA -GSL-YVTS-DEFI--T-MHK
Phalacrocorax carbo-Gamma-5K	KFW78608.1	EL---RPD---Y--CNE-LI-LSN PGA -GSL-YVTS-DEFI--T-MHK
Chaetura pelagica-Gamma-5K	KFU93981.1	EL---RPD---Y--CNE-LI-LSN PGA -GSL-YVTS-DEFI--T-MHK
Balearica regulorum gibbericep-Gamma-5K	KF006958.1	EL---RPD---Y--CNE-LI-LSN PGA -GSL-YVTS-DEFI--T-MHK
Patagioenas fasciata monilis-Gamma-5K	OPJ77564.1	EL---RPD---Y--CNE-LI-LSN PGA -GSL-YVTS-DEFI--T-MHK
Apaloderma vittatum-Gamma-5K	KFP77981.1	EL---RPD---Y--CNE-LI-LSN PGA -GSL-YVTS-DEFI--T-MHK
Colius striatus-Gamma-5K	KFP30926.1	EL---RPD---Y--CNE-LI-LSN PGA -GSL-YVTS-DEFI--T-MHK
Acanthisitta chloris-Gamma-5K	XP_009079275.1	EL---RPD---Y--CNE-LI-LSN PGA -GSL-YVTS-DEFI--T-MHK
Corvus brachyrhynchos-Gamma-5K	KF053565.1	EL---RPD---Y--CNE-LI-LSN PGA -GSL-YVTS-DEFI--T-MHK
Melopsittacus undulatus-Gamma-5K	XP_005140981.2	EL---RPD---Y--CNE-LI-LSN PGA -GSL-YVTS-DEFI--T-MHK
Geospiza fortis-Gamma-5K	XP_014165085.1	EL---RPD---Y--CNE-LI-LSN PGA -GSL-YVTS-DEFI--T-MHK
Leptosomus discolor-Gamma-5K	XP_009951577.1	EL---RPD---Y--CNE-LI-LSN PGA -GSL-YVTS-DEFI--T-MHK
Antrostomus carolinensis-Gamma-5K	XP_010166918.1	EL---RPD---Y--CNE-LI-LSN PGA -GSL-YVTS-DEFI--T-MHK
Anas platyrhynchos-Gamma-5K	EOB01100.1	EL---RPD---Y--CNE-LI-LSN PGA -GSL-YVTS-DEFI--T-MHK

Amphibians PIP5K γ

Xenopus laevis-Gamma-5K	AAH55973.1	EL---KPD---Y--CSE-LI-LSN PGA -GSI-FVTS-DEFI--T-QHK
Xenopus tropicalis-Gamma-5K	NP_001120474.1	EL---RPD---Y--CNE-LI-LSN PGA -GSL-YVTS-DEFI--T-MHK
Nanorana parkeri-Gamma-5K	XP_018421916.1	EL---RPD---Y--CNE-LF-LCN SGA -GSL-YV-G-DEFI--T-QHK

Reptiles PIP5K γ

Gekko japonicus-Gamma-5K	XP_015276073.1	EL---RPD---Y--CNE-LI-LSN PGA -GSL-YVTS-DEFI--T-MHK
Python bivittatus-Gamma-5K	XP_007441380.1	EL---RPD---Y--CNE-LI-LSN PGA -GSL-YVTS-DEFI--T-MHK
Protobothrops mucrosquamatus-Gamma-5K	XP_015666168.1	EL---RPD---Y--CNE-LI-LSN PGA -GSL-YVTS-DEFI--T-MHK

Fishes PIP5K γ

Poecilia latipinna-Gamma-5K	XP_014911115.1	EL---RPD---Y--CNE-LI-LSN PGA -GSI-YVTS-DEFI--T-LHK
Xiphophorus maculatus-Gamma-5K	XP_005797142.2	EL---RPD---Y--CNE-LI-LSN PGA -GSI-YVTS-DEFI--T-LHK
Fundulus heteroclitus-Gamma-5K	XP_012729576.1	EL---RPD---Y--CNE-LI-LSN PGA -GSI-YVTS-DEFI--T-LHK
Cyprinodon variegatus-Gamma-5K	XP_015241514.1	EL---RPD---Y--CNE-LI-LSN PGA -GSI-YVTS-DEFI--T-LHK
Poecilia mexicana-Gamma-5K	XP_014856633.1	EL---RPD---Y--CNE-LI-LSN PGA -GSI-YVTS-DEFI--T-LHK
Poecilia formosa-Gamma-5K	XP_007571215.1	EL---RPD---Y--CNE-LI-LSN PGA -GSI-YVTS-DEFI--T-LHK
Stegastes partitus-Gamma-5K	XP_008304116.1	EL---RPD---Y--CNE-LI-LTN PGA -GSI-YVTR-DEFI--T-MHK
Neolamprologus brichardi-Gamma-5K	XP_006786750.1	EL---RPD---Y--CNE-LI-LSN PGA -GSI-YVTR-DEFI--T-LHK
Haplochromis burtoni-Gamma-5K	XP_005920620.1	EL---RPD---Y--CNE-LI-LSN PGA -GSI-YVTR-DEFI--T-LHK
Pundamilia nyererei-Gamma-5K	XP_005721179.1	EL---RPD---Y--CNE-LI-LSN PGA -GSI-YVTR-DEFI--T-LHK
Maylandia zebra-Gamma-5K	XP_012772289.1	EL---RPD---Y--CNE-LI-LSN PGA -GSI-YVTR-DEFI--T-LHK
Seriola lalandi dorsalis-Gamma-5K	XP_023267354.1	EL---RPD---Y--CNE-LI-LTN PGA -GSI-YVTR-DEFI--T-LHK
Seriola dumerili-Gamma-5K	XP_022610606.1	EL---RPD---Y--CNE-LI-LTN PGA -GSI-YVTR-DEFI--T-LHK
Cynoglossus semilaevis-Gamma-5K	XP_016897956.1	EL---RPD---Y--CNE-LI-LTN PGA -GSI-YVTR-DEFI--T-QHK
Lates calcarifer-Gamma-5K	XP_018532494.1	EL---RPD---Y--CNE-LI-LTN PGA -GSI-YVTR-DEFI--T-QHK
Paralichthys olivaceus-Gamma-5K	XP_019956130.1	EL---RPD---Y--CNE-LI-LTN PGA -GSI-YVTR-DEFI--T-QHK
Kryptolebias marmoratus-Gamma-5K	XP_017277796.1	EL---RPD---Y--CNE-LI-LAN PGA -GSI-YVTR-DEFI--T-QHK
Austrofundulus limnaeus-Gamma-5K	XP_013876251.1	EL---RPD---Y--CNE-LI-LAN PGA -GSI-YVTR-DEFI--T-QHK
Amphiprion ocellaris-Gamma-5K	XP_023121344.1	EL---RPD---Y--CNE-LI-LTN PGA -GSI-YVTR-DEFI--T-QHK
Notothenia coriiceps-Gamma-5K	XP_010784186.1	EL---RPD---Y-ICNE-LI-LTN PGA -GSI-YVTR-DEFI--T-QHK
Acanthochromis polyacanthus-Gamma-5K	XP_022046423.1	EL---RPD---Y--CNE-LI-LTN PGA -GSI-YVTR-DEFI--T-QHK
Nothobranchius furzeri-Gamma-5K	XP_015812680.1	EL---RPD---F--CNE-LI-LAN PGA -GSI-YVTR-DEFI--T-LHK
Oryzias latipes-Gamma-5K	XP_004079345.2	EL---RPD---Y--CNEALI-LTN PGA -GSI-YVTR-DEFI--T-MHK
Oryzias melastigma-Gamma-5K	XP_024129977.1	EL---RPD---Y--CNEALI-LTN PGA -GSI-YVTR-DEFI--T-QHK

Other Eukaryotes

Ciona intestinalis-5K	XP_018673474.1	RL---RPD--ML-IS-L-LR-LSN PGA -GSL-FVTH-DEFI--T-QHK
Ciona intestinalis-4K	XP_002119441.3	E----A-K--VS--CV-Q-FRVDD KG- -GS---H-F-HKY---TLNG-
Oikopleura dioica-5K	CBY09966.1	-I-N-STE-F-H-IGARSLIPIGN PGA -GSC-W-TH-DEFIV-T-QQK
Oikopleura dioica-4K	CBY32977.1	-I-D-SVEG-IH-IIHE-LTAIGN PGA -GSL-W-TH-DEFI--T-DNK
Branchiostoma floridae-5K	XP_002591361.1	QL---QPD-F-I--CSE-LR-LSN PGA -GSL-YLTA-DEFIV-T-QQK
Branchiostoma floridae -4K	XP_002599487.1	E--N---V--MN----SQ-VNTDS PG- -GA---M---KRY---TIE--
Apostichopus japonicus-5K	PIK60516.1	-L---QPD---I--VKD-LR-LSN PGA -GSI-YL-N-DEFI--TC-IK
Apostichopus japonicus-4K	PIK54083.1	S--TVAETE-RN-F-FG--EYDNS SGK -GAK-MKTH--RF---TITR-
Saccoglossus kowalevskii-5K	XP_006821157.1	EL---QPD-F-L--CNDLLR-LSN PGA -GSV-YLTS-DEFI--T-QHK
Saccoglossus kowalevskii-4K	XP_002732674.1	EK---N-TE-MN-M-SAE-VYNDS PG- -GA-----A-KKY---TI-R-
Apis mellifera-5K	XP_006571145.1	EL---QPD-F-M-MCSA-LR-LSN PGA -GSI-YLTD-DEFI--T-QHK
Drosophila melanogaster-5K	NP_611729.2	EL---QPD-FMM-MCTS-LR-LSN PGA -GSI-YLTT-DEFI--T-QHK
Apis mellifera-4K	XP_392797.3	E------L--KE-M--SQ-ILEDS SGK -GAK-YQ---KLFI--TLTG-
Drosophila melanogaster-4K	NP_001033805.1	E---V--V--RE----SQ-IQIDS SGK -GAQ-YQ---KFFI--SLT--
Caenorhabditis elegans-5K	NP_491576.2	NL-H-KPA-F-R-ICTE-LK-LSN AGA -GSI-YV-Q-DQFI--T-QHK

Caenorhabditis brenneri-5K	EGT37979.1	NL-S-KPA-F-R-ICTE-LK-LSN AGA -GSI-YV-Q-DQFI--T-QHK
Wuchereria bancrofti-5K	EJW88844.1	-L-A-KTA-F-R-VCMF-LK-LSN AGA -GSI-YV-H-DQFI--T-Q-K
Trichinella britovi	KRY55394.1	EL-N-KPE-FMA--CGV-LR-LSN PGA -GSI-Y--S-DKFIV-T-QHR
Trichinella spiralis	KRY35256.1	EL-N-KPE-FMA--CGV-LR-LSN PGA -GSI-Y--S-DKFIV-T-QHR
Brugia malayi-5K	CDP97235.1	-L-A-KTA-F-R-VCMF-LK-LSN AGA -GSI-YV-H-DQFI--T-Q-K
Loa loa-5K	XP_020303847.1	-L-A-KTA-F-R-ICMF-LK-LSN AGA -GSI-YV-H-DQFI--T-Q-K
Strongyloides ratti-5K	CEF65455.1	EM-N-KPA-F-R--CTQ-LR-LSN PGA -GSV-YV-S-DKFI--T-QYK
Toxocara canis-5K	KHN83910.1	LGHRRI-RQGER-ICTE-LK-LSN AGA -GSI-YV-S-DQFI--T-QHK
Trichinella papuae-4K	KRZ69085.1	EL-N-KPE-FMA--CGV-LR-LSN PGA -GSI-Y--S-DKFIV-T-QHK
Biomphalaria glabrata-beta-5K	XP_013074799.1	EL---QPD-FML--CDE-LK-LSN PGA -GSI-YLTQ-DEFI--T-QHR
Octopus bimaculoides-alpha-5K	XP_014789196.1	EL---HAD-FML--CTE-LQ-LCN PGA -GSI-Y-TD-DEFI--T-QHK
Crassostrea virginica-5K	XP_022306935.1	EL-S-QTD-F-L--CDE-LK-LSN PGA -GSI-YL-Q-DEFI--T-QHK
Mizuhopecten yessoensis-5K	XP_021350909.1	EL---QPD-F-L--CDD-LK-LSN PGA -GSI-YLTS-DEFI--T-QHK
Crassostrea virginica-4K	XP_022305822.1	E---------MN-- TK--EDIDS PG- -GA-MMM-H-KKYF--TLV--
Mizuhopecten yessoensis-4K	XP_021377781.1	E---------MN-- TKQAVDVDS PG- -GA-M-M-Q-KKFFV-TLV--
Octopus bimaculoides-alpha-4K	XP_014773204.1	E--SV--I--MN-- TKQ-V-MDS PG- -GA-M-L-Q-KKYF--TLV--
Biomphalaria glabrata-beta-4K	XP_013081123.1	E---L--N--MN--SKQQ-ISCDS PG- -GA-M-M-R-KRFF--TLV--
Helobdella robusta-5K	XP_009026309.1	-L---QPE-F-L--CNE-LK-LSN PGA -GSI-YL-N-DEFI--T-QHK
Capitella teleta-5K	ELT87243.1	EL---QPD-F-L--CNE-LQ-LSN PGA -GSI-Y-TN-DEFI--T-QRK
Helobdella robusta-4K	XP_009023323.1	E--SV-EDC-MN--VKHC-TDIDS PGK -GA-----H--KYIL-T
Capitella teleta-4K	ELU08768.1	E--D--EDT-GN---KFC-YDCDS SG- -GA---H-W-KKF-V-TIL--
Macrostomum lignano-5K	PAA67906.1	KLYN--ISQF-A-ICGEELE-LSN PGA -GSI-YRTA-DNFI--T-QHK
Clonorchis sinensis-5K	GAA56497.1	T-YKL-IR-F-N-ICSRELR-LSN PGA -GSI-Y-TQ-DEFI--T-QHR
Macrostomum lignano-4K	PAA91900.1	SK---ERTE--D-FIKRQ-QYDAS -G- -GSK--CT-N-HY---TIG--
Clonorchis sinensis-4K	GAA55841.1	E---VNKL--WDAF-GYQ-LWDSA RGK -GSK--VT-N-QF-A-AI---
Hypsibius dujardini-5K	OQV12309.1	EL---QPD--ML--CDRAMI-ISN PGA -GSI-YLTE-DEFIL-T-MYR
Ramazzottius varieornatus-5K	GAU91082.1	EV-D-SPE--MM-ICVEGLI-ISN PGA -GSL-YLTE-EMFI--T-DHS
Hypsibius dujardini-4K	OQV16814.1	E--KVT-SQ-ML---SSE-IIKDT HGG QGSTYYLTA-KRFI--TLTR-
Ramazzottius varieornatus-4K	GAV02130.1	E--KVT-SQ-ML---ASE-IMKDV HD- QGSNYYLTA-KRFI--TLTR-
Trichoplax adhaerens-5K	XP_002108154.1	EL---KP--FMI-MCDKRLK-IRN PGA -GSL-FLTN-DRFI--T-QKK
Trichoplax adhaerens-4K	XP_002111279.1	EC-D---EQFKQ-IAFS-LM Q YGD -G K-FV-R-KQY-V-TID-Y
Amphimedon queenslandica-5K	XP_019849193.1	EA-Q-KAE---LA-CHQSLR-LSN PGA -GSL-YL-A-DEFI--T-QKK
Amphimedon queenslandica-4K	XP_019863884.1	K--K---Y--MS---QHAHLAMDN PG- -GST-FVTS-KK-I--SL---
Hydra vulgaris-5K	XP_012564577.1	-L---QPS-F-L--ANE-IK-ISN PGA -GSL-FV-N-DMFIV-T-THK
Nematostella vectensis-5K	XP_001633067.1	EL-SMRPD-FMMA-CNE-LV-LSN PGA -GSL-YVTC-DEFI--T-QKK
Exaiptasia pallida-5K	XP_020899131.1	EL-SMKAD-FMM--CND-LQ-LSN PGA -GSL-YVTC-NQFI--T-DHK
Hydra vulgaris-4K	XP_002161268.1	E--N-EE-L-AR-FLIQ -CD-NA SGN -GAK---TKNKMFY--TIER-
Exaiptasia pallida-4K	XP_020903371.1	E---VN--ELAK-FLES -LA-SS -G- -GAK-FT-RNKKFY--TIE--
Nematostella vectensis-4K	XP_001647531.1	E----E-K-LAEAFQVP -IS-DS PG- -GAK-FL-QNKRFYV-VIE--
Salpingoeca rosetta-5K	XP_004997164.1	-I-N--TA-F-L-MCHK-LR-LSN PGA -GSL-WL-H-DRFIV-TIQKG
Salpingoeca rosetta-4K	XP_004998565.1	-I-N--TA-F-L-MCHK-LR-LSN PGA -GSL-WL-H-DRFIV-TIQKG
Monosiga brevicollis-5K	EDQ89244.1	EK-S--TG-F-M-MCDS-LR-LSN AGA -GSL-WL-H-DLFIV-T-QKG
Monosiga brevicollis-4K	EDQ89588.1	EK-S--TG-F-M-MCDS-LR-LSN AGA -GSL-WL-H-DLFIV-T-QKG
Capsaspora owczarzaki-5K	XP_004348939.1	EA---KAE-FML--CNE-LR-LSN PGA -GSL-YM-HNDHFI--T-QRR
Capsaspora owczarzaki-4K	XP_004364933.1	E---V-ADQ--Y--AGAE-IPV-A NGK -GAS-YMTH-KRFIV-SM-KI
